# A new wide-scope, multi-biomarker wastewater-based epidemiology analytical method to monitor the health and well-being of inhabitants at a metropolitan scale

**DOI:** 10.1007/s00216-025-06097-3

**Published:** 2025-09-13

**Authors:** Harry Elliss, Kit Proctor, Megan Robertson, John Bagnall, Barbara Kasprzyk-Hordern

**Affiliations:** 1 Department of Chemistry, University of Bath, Claverton Down, Bath, BA2 7AY UK; 2 Centre of Excellence in Water-Based Early-Warning Systems for Health Protection, University of Bath, Claverton Down, Bath, BA2 7AY UK; 3 Institute of Sustainability and Climate Change, University of Bath, Claverton Down, Bath, BA2 7AY UK; 4 Chemical Characterisation Facility, University of Bath, Claverton Down, Bath, BA2 7AY UK; 5 Wessex Water Service Ltd, Claverton Down, Bath, BA2 7WW UK

**Keywords:** Community health, Wastewater-based epidemiology (WBE), Public health

## Abstract

This manuscript establishes a new, comprehensive biomarker list and a multiresidue trace quantification method for community-wide health and well-being assessment at a metropolitan scale using wastewater-based epidemiology (WBE) and mass spectrometry pipelines. This method enables the quantification of 204 biochemical indicators (BCIs) across a range of biomarker classes within influent wastewater and includes illicit drug BCIs, pharmaceuticals as proxies for disease, health markers (hormones, oxidative stress, lipid peroxidation, etc.), Lifestyle chemicals, food BCIs, and hazardous chemicals in personal care products. This method facilitates the combined assessment of community exposure to chemicals and the effects of this exposure in the same framework. The method enables full quantification of 141 BCIs with method detection Limits varying from 0.01 ng/L for amlodipine to 23.8 ng/L for stachydrine. Total average method accuracies were 102.7% whereas precision was 10.4%. During an initial assessment of this method to test its suitability, 62% of all targets were detected and quantified during a week-long feasibility study of a large city with weekly average Daily BCI loads ranging from 40.0 ± 20.0 mg/day for salbutamol to 5836.5 ± 1697.1 g/day for creatinine. The inclusion of new endogenous markers such as advanced glycation end products, detected in wastewater for the first time, enables more accurate determination of community-level health and lifestyle habits. Alongside an unbiased and comprehensive health assessment through endogenous markers, health is further assessed via the use of pharmaceuticals, acting as a proxy for health and disease status whilst additionally providing insights into community lifestyle habits through the monitoring of licit/illicit drug use and food consumption. The analysis of all biomarker classes combined aims to provide insights to exposure and health effect outcomes at the community level.

**Graphical Abstract:**

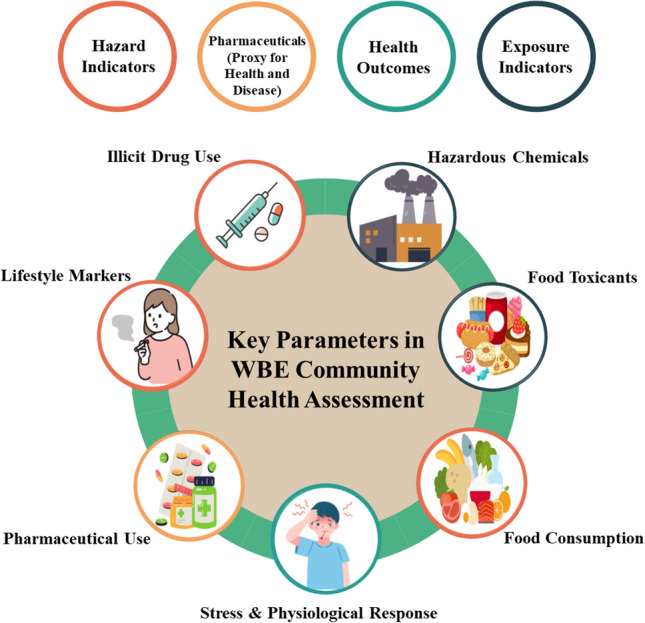

**Supplementary Information:**

The online version contains supplementary material available at 10.1007/s00216-025-06097-3.

## Introduction

Over the past decade, rapid global urbanisation has led to projections that 68% of the world’s population will Live in urban areas by 2050, increasing from 55% in 2018 [1]. Giles-Corti et al. highlight that the liveability of cities and the “health and well-being of the residents” are intrinsically linked; this projected population increase will not only strain the city’s infrastructure but also place more significant stress on the given community [2]. Recent studies have indicated that leading an unhealthy Lifestyle can lead to a 78% increased chance of early death [3], whereas making health-conscious decisions, such as with diet, can increase Life expectancy by up to 10 years [4]. Due to various lifestyle decisions alongside external stressors [5], urgent community-wide public health assessments are required. Wastewater-based epidemiology (WBE) is well poised as a non-invasive, anonymous surveillance technique, operating on a metropolitan scale, to apply the required interventions.

The health of communities is intertwined with their lifestyle, something WBE has been extensively applied to – the consumption of illicit drugs, e.g. traditional drugs such as cocaine or marijuana [6, 7]. More recently, WBE has reported the emergence of new psychoactive substances where their unknown human effect can have a critical impact on the health of a community [8, 9]. Alongside illicit drugs, licit substances such as caffeine and nicotine were extensively studied [10] and applied at both the local [11] and continental [12] levels. A popular approach within community health assessments in WBE is utilising pharmaceutical use as a proxy for disease status and health. For example, via the analysis of pharmaceuticals, population-level assessments of the following diseases have been made possible, such as pain [13], depression [14], gout [15], hypertension [16] and diabetes [17]. These assessments are made possible by utilising parent and metabolite drug combinations alongside excretion data to estimate the population’s normalised daily intake of certain pharmaceuticals [18].

A potential pitfall of WBE pharmaceutical analysis in the context of Health assessments is the inability to distinguish between pharmaceutical dosages. For example, four people ingesting one 50 mg dose will appear equal to one person ingesting one 200 mg dose [19]. It is important to note that only in certain countries, prescription data will be able to predict usage and assist with these challenges [20, 21]. To circumnavigate this issue, expanding the breadth of endogenously formed compounds which can be detected in wastewater is critical to provide a truly unbiased understanding of physiological response to external stressors. Various health markers have been applied in WBE such as C-Reactive Protein [22], cortisol [23, 24], prostaglandin E2 [25] and 8-iso-prostaglandin F2_α_ [26]. A major challenge encountered during the analysis of the targets is the low concentration levels in community wastewater, leading to feasibility studies often being conducted to a smaller population size such as a university campus [26, 27]. Despite this, certain endogenous biomarkers have also displayed good wastewater stability [28] which are critical to daily assessments of stress marker production [29], indicating their strong potential to assess community health via WBE. Alongside, stress The analysis of hormones is critical to understanding community health as they underpin physiological condition and variable load of these biomarkers could indicate endocrine disruption or other potential external stressors [30]. It is important to increase the breadth of available endogenous markers to provide a holistic understanding of health. Due to the inseparable link between food and health – food consumption estimates via WBE have been used to assess healthy eating habits, from artificial sweeteners [31] to phytoestrogens [32] and vitamins [33]. Critical for WBE food analysis is the use of gut metabolites, such as equol, to distinguish food waste and food consumption [23], due to the complex nature of gut metabolism – to further understand community-level food consumption, it is important to expand on this sub-class. Furthermore, clearer understanding in elucidating trends within WBE to further understand the health of a region is using additional information such as the socio-economic status, which can further identify BCIs that could indicate other factors such as income or housing [33–36].

**Fig. 1:**
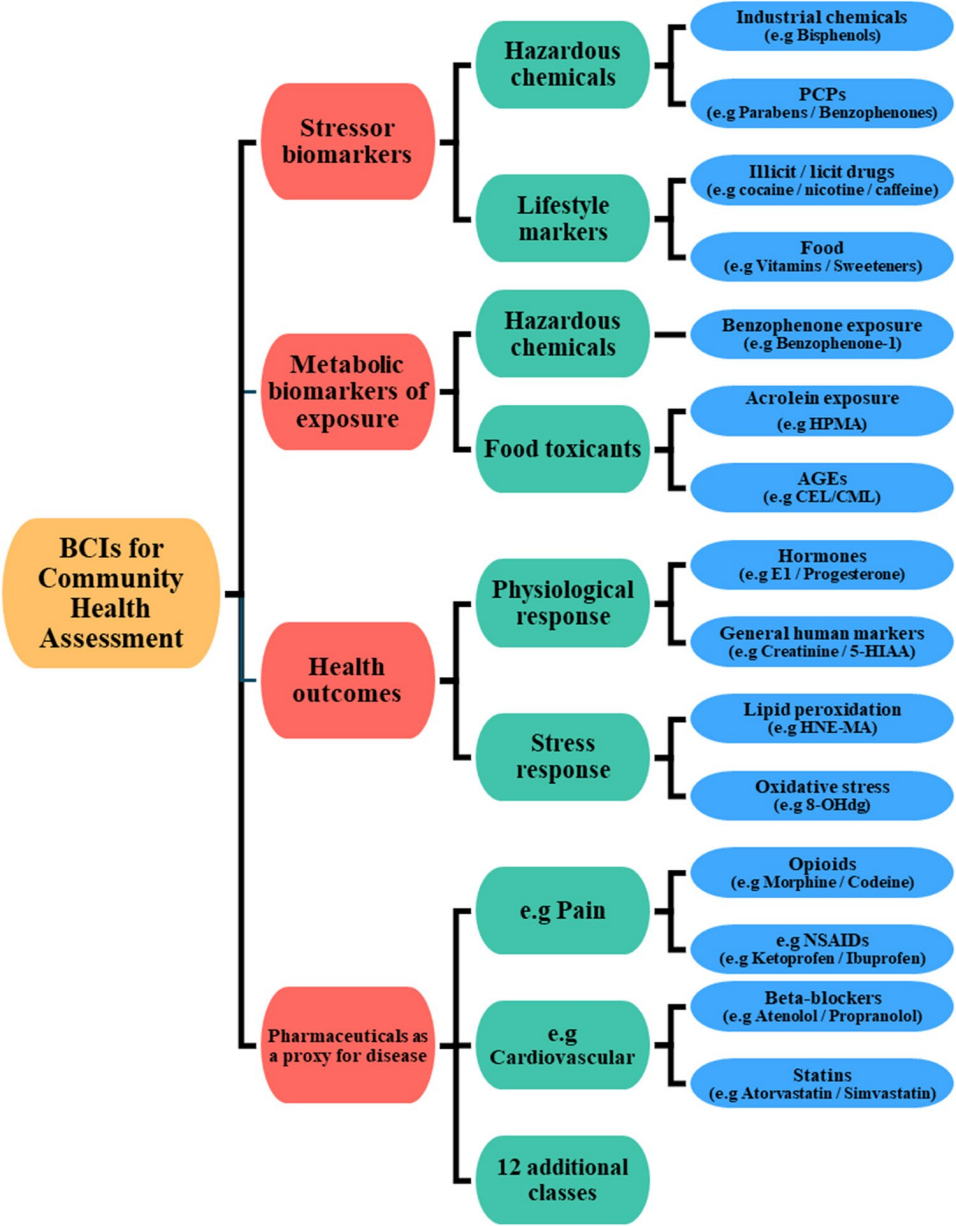
An overview of some BCI classes under study within this manuscript and how they can be collated to allow WBE to provide a comprehensive understanding of community health, at the community scale

Besides biochemical indicators (BCIs), it is also important to monitor links between lifestyle and exposure to hazardous chemicals, not intended for human consumption. Parabens and benzophenones are widely used in personal care products such as sunscreens and cosmetics; however, both have or are suspected to be endocrine-disrupting chemicals [37–39]. They have both been detected in wastewater due to high community use [40, 41]; however, the latter also exhibits seasonal usage peaks [42] and, therefore, potentially seasonal exposure routes, such as the consumption of certain foods, something which can be assessed in tandem with the other BCIs within this manuscript. An overview of some of the BCI classes included within this method is highlighted below in Fig. 1.

Figure 1, above, provides initial insight to how different BCIs can be analysed to provide indicators of the health of a community. Table S1 expands on Fig. 1, detailing the specific sub-groups of compounds, alongside their abbreviations, which are included within this analytical method. This manuscript aims to develop a new wide-scope analytical method to interpret the well-being of inhabitants at a metropolitan scale. The objectives of the study are:

1. Development and validation of an analytical LC–MS/MS method for over 200 compounds spanning stressors (e.g. hazardous chemicals), metabolic biomarkers of exposure, biomarkers of physiological response, and pharmaceuticals as proxies for disease.
2. Application of the method in a weeklong city monitoring.

## Materials and methods

### Reagents and materials

The standards, alongside isotopically labelled standards, of suitable analytical grade were purchased from Sigma-Aldrich (Gillingham, UK), TRC (Toronto, Canada), Cambridge Biosciences (Cambridge, UK) and LGC (Teddington, UK). Details of the supplier for all analytical standards, alongside CAS number, are detailed in Table S2. All standards were purchased either in 0.1–1 mg/mL solutions or as powders and subsequently dissolved in MeOH to 1.0 mg/mL. In certain scenarios, alternative solvents such as water or dimethyl sulfoxide were used in small ratios (respective to methanol) to boost solubility. Due to poor solubility in MS suitable solvents, 3-nitro-L-tyrosine, 4-pyridoxic acid, 8-hydroxyguanine, nicorandil, neopterin, pterin and riboflavin were made into solution with a final concentration x, in the concentration range: 0.05 mg/mL < x < 0.15 mg/mL. All stock solutions were stored in the freezer (< −20 °C). Multi-analyte class mixes were prepared at a lower concentration, nominally 5 µg/mL.

All solvents used for mobile phase or sample preparation were LC–MS grade (Supelco, Sigma-Aldrich). Mobile phase additives, formic acid, ammonium formate, and ammonium fluoride were also purchased from Sigma-Aldrich.

### Sample collection

Composite (24-h, time proportional) samples of wastewater influent were taken after physical screening, with an ISCO 5800 autosampler (15 mL every 15 min). The daily flow rate is detailed in Table S3. During the 24-h period where sampling was taking place, the wastewater was maintained at ~ 4 °C by a refrigeration unit, installed within the autosampler. Following sample collection, wastewater was transported, on ice, to the laboratory for sample extraction and preparation.

### Sample extraction and preparation

Influent wastewater was aliquoted into two sets of 50 mL duplicates, one for extraction following an ion-exchange solid-phase extraction (SPE) protocol, the other a neutral SPE protocol. Following method development, two SPE protocols were deployed to enable the quantification of a broad range of analytes; mixed-mode cation exchange was used due to the wider appearance of amines within the low concentration analytes reported within the method. Other cartridges were tested, Oasis PRiME HLB and Oasis MAX (Waters, Manchester) however, they were not taken for further consideration due to the added time and cost it took for only a minimal improvement in detection rate. Precision was improved for a limited number of analytes with MAX cartridges; however, this was not significant, especially when compared with MCX cartridges.

#### Neutral SPE

One set of the duplicates was taken and each replicate was spiked with 50 µL of an internal standard mix (Table S4), as described above, and left to equilibrate for 30 min. Following equilibration, each replicate was filtered using a 0.7 µm GF/F filter (Whatman, Fisher Scientific) and then prepared for extraction using 60 mg (3 cc), Hydrophilic-Lipophilic Balance (HLB) cartridges (Waters, Manchester). Prior to sample loading, each SPE cartridge was conditioned with 2 mL MeOH (LC–MS grade) and equilibrated with 2 mL H_2_O (LC–MS grade); both steps were performed under gravity. The sample was loaded onto the cartridge under a Light vacuum at approximately 5 mL/min; after this, the cartridge was dried under vacuum for approximately one hour.

Once the cartridge had dried, it was eluted with 4 mL MeOH (LC–MS grade) into silanised glass tubes (Fisher, Loughborough) and dried down using a stream of nitrogen (~ 5 psi) at 40 °C within a Turbovap LV concentration workstation (Caliper, UK). Following complete evaporation, dried extractions were reconstituted using 100 µL of methanol, vortex mixed, and further diluted with 400 µL water before transferring to LC polypropylene vials (Waters, Manchester) for LC–MS/MS analysis.

#### Ion exchange SPE

The other duplicate set was spiked with 25µL of internal standard mix, as described above and pH adjusted to pH 2.0 with 5 M HCl and left to equilibrate for 30 min. Ion exchange SPE cartridges were filtered via the same method as discussed in Sect."Neutral SPE"; however, the cartridge used this time was a 60 mg (3 cc), Mixed-Mode Cation Exchange (MCX) cartridge (Waters, Manchester). Prior to sample loading, each SPE cartridge was conditioned with 2 mL MeOH (LC–MS grade) and equilibrated with 2 mL H_2_O (LC–MS grade) + 0.5% (v/v) formic acid. Much Like above, both steps were performed under gravity. The sample was loaded under a Light vacuum at approximately 5 mL/min; after this, the cartridge was washed with 2 mL H_2_O (LC–MS grade) + 2.0% (v/v) formic acid and dried under vacuum for approximately 30 min.

The cartridge was eluted in a stepwise manner, both into silanised glass tubes, firstly with 3 mL methanol (LC–MS grade), followed by the second elution where 3 mL methanol (LC–MS grade) + 5% (v/v) ammonium hydroxide (purchased as 1 M NH_3_ in H_2_O, Sigma-Aldrich). Both eluents were evaporated, following the method detailed above in 2.3.1. However, each vial was reconstituted using 50 µL and 200 µL of methanol and water, respectively. Figure S1 demonstrates a summary of the workflow applied for sample preparation using HLB and MCX cartridges.

Despite additional steps required to prepare cartridges via ion exchange SPE, the additional selectivity coupled with the broad scope of the HLB cartridge means that when they work together, the broad range of analytes under study in the method can be quantified in influent wastewater.

### Ultra-high performance liquid chromatography

Liquid chromatography was performed using an Acquity UPLC system manufactured by Waters (Manchester, UK).

#### Column selection

Prior to method validation, four different column stationary phases were trialled to assess key parameters such as suitable separation and peak intensity during standard injections, focusing on key analytes with a range of physicochemical properties. BEH C_18_, HSS T3, pentafluorophenyl, and biphenyl columns were compared, with C_18_ and biphenyl stationary phases providing the best performance. Despite improved separation with the biphenyl column, the C_18_ performed best across the range of analytes required to be analysed within this method and therefore was the selected stationary phase moving forward (data not shown). Following stationary phase assessments, a comparison of method performance between two column dimensions was evaluated: 1 × 150 mm vs 2.1 × 50 mm. Both showed similar performance, with the latter having a shorter run time and therefore improved sample throughput; however, improved resolution and reduced solvent use with the long column meant the Waters BEH C18, 1.7 µm, 1.0 mm × 150 mm was selected to be used for this method.

#### Mobile phase and gradient selection

A mobile phase composition of 95:5 H_2_O:MeOH was used as the starting point for method development with varying assessments of aqueous:organic composition; it was determined that mirroring the composition of the injected sample (80:20, H_2_O:MeOH – see Sect."Sample extraction and preparation".) gives the most reproducible peak shape and prevents the formation of organic microenvironments leading to poor peak shape. This was particularly noticeable with the increasingly polar ‘health marker’ BCIs. Two UHPLC methods were developed to allow mass spectrometry analysis under ESI + and ESI − ionisation modes.

The method for ESI + was modified from Ceolotto et al. [18]. Alongside composition, the introduction of an ammonium formate buffer salt, to Limit retention time shifting whilst not coming at a cost of the sensitivity, 2 mM gave this best combination when compared to 5 mM and 10 mM ammonium formate. These comparisons occurred at the same pH to ensure differentiation to performance was due to the buffer concentration. The addition of 0.1% (v/v) formic acid meant the final pH was pH 3, within the buffering capacity of ammonium formate. The final mobile phase A composition was 80:20 H_2_O:MeOH + 2 mM ammonium formate + 0.1% formic acid; mobile phase B (MPB) was 100% methanol. During gradient optimisation, the initial gradient was slowed, which gave improved peak shape and separation of polar analytes. The gradient used was 0 min(0% B), 3.5 min(4% B), 21 min(100% B), 28 min(100% B), 28.1 min(0% B), 34.5 min(0% But the run, a constant flow rate of 0.04 mL/min was used with a column temperature of 25 °C.

The method for ESI- was modified from Petrie et al. [43]. Key novelty and new applications of this method occur via the introduction of over 30 new BCIs, predominantly in the ‘food’ sub-category. Switching from a neutral mobile phase (pH 6.5) to an acidic mobile phase (pH 3.5) saw improvements in polar analytes such as acesulfame K; however, this came at a cost of many other analytes and therefore was not taken forward. The LC conditions for the negative ionisation mode are mobile phase A: 80:20 water:methanol + 1 mM ammonium fluoride and mobile phase B: 95:5 methanol:water + 1 mM ammonium fluoride. The gradient is as follows: 0–0.5 min 0%B, 0.5–2.5 min 60%B, 2.5–8.0 min 100%B, 8.0–14.0 min 100%B, 14.0–14.1 min 100%A, 14.1–22.5 min 100%A. Similar to the ESI + method, a constant flow rate of 0.04 mL/min was used with a column temperature of 25 °C.

Total ion chromatograms of all analytes in the positive and negative ionisation modes can be found in Figures S2 and S3, respectively.

### Mass spectrometry

Detection and subsequent quantification of analytes (target compounds and isotopically labelled standards) was performed using a Xevo TQD (Waters, Manchester) triple quadrupole mass spectrometer equipped with an electrospray ionisation (ESI) source, operated in both positive and negative ionisation mode, depending on the analytes under study. In all cases, nitrogen was used as the desolvation and nebulisation gas, whereas argon was used as the collision gas. The mass spectrometer was operated in MRM mode, regardless of positive or negative ionisation.

In positive ionisation mode, the following mass spectrometry conditions were used: the capillary voltage was 3.20 kV, the desolvation temperature and gas flow were 400 °C and 700 L/hour, respectively. The cone gas flow was 100 L/hour and the source temperature was 150 °C. In negative ionisation mode, the capillary voltage was 3.00 kV; all other conditions were the same as in the positive mode.

All analytes were tuned via direct infusion of a 1.0 µg/mL solution in 80:20 H_2_O:MeOH with the mass spectrometer operating in ‘combined’ mode so that mobile phase was also flowing through the system to provide more realistic values for cone voltage (V) and collision energy (eV). In all cases, the most abundant ion was [M-H]^+^ and [M-H]^−^, ionisation mode depending. All analytes have 2 transitions, a quantification and qualification trace; these transitions alongside cone voltage and collision energy are found in Table S5. Internal standards have one transition; these are reported in Table S6. Minimum dwell time was 8 ms with, in most cases, 6 points above the full-width half-maximum. Mass spectral data processing was conducted with V4.1 MassLynx (Waters, Manchester) and TargetLynx (Waters, Manchester).

### Instrument and method performance

To quantify the analytes under study, 19-point calibration curves from 0.01 ng/mL up to 2000 ng/mL were run, with a constant internal standard concentration of 100 ng/mL. Calibration curves were used to assess the instrument detection limit (IDL) and instrument quantification limit (IQL) using the signal–noise (S/N), S/N ≥ 3: detection, S/N ≥ 10: quantification. A complete evaluation of all method parameters, including retention time, relative retention time (RRT) (Eq. 1), accuracy (Eq. 2) and precision (Eq. 3) is displayed in Table S7 and 1 below. Accuracy and precision were determined throughout the analysis via the injection of 3 quality control concentrations: 20, 200, and 500 ng/mL.

1 $$RRT= \frac{{RT}_{STD}\left(minutes\right)}{{RT}_{ISTD}\left(minutes\right)}$$

where RRT is the relative retention time and RT_STD_ is the retention time of the target analyte and RT_ISTD_ is the retention time (minutes) of the internal standard which has been assigned to quantify the given target analyte.

2 $$Accuracy \left(\%\right)=\left(\frac{{C}_{spiked}- {C}_{unspiked} }{{C}_{theo}}\right)\times 100$$

where C_spiked_ is the experimentally determined concentration of the analyte under study, when added to the sample, C_unspiked_ is the experimentally determined concentration of the analyte under study, native to the sample, and C_theo_ is the theoretical concentration. Throughout this method it is the medium QC which is used to calculate accuracy and precision. This is performed in 80:20 H_2_O:MeOH & influent wastewater, to understand relative recovery, post-SPE.

3 $$Precision= \sqrt{\frac{{\sum }_{i = 1}^{n}{\left(x-\overline{x }\right)}^{2}}{n-1}}$$

where $x$ is the experimentally determined accuracy and $\overline{x }$ is the average accuracy, n is the number of replicates applied. Throughout this validation it was the medium QC which was used to calculate accuracy and precision.

4 $$Daily\;Load\left(\frac{mg}{day}\right)=Concentration\;\left(mg/L\right)\times Flow\;\left(L/day\right)$$

## Results and discussion

The broad range of biomarkers captured by this method enables a wider understanding of community health by analysing various sub-groups covering illicit drugs, lifestyle chemicals, pharmaceuticals, food, human markers, and PCPs. All analytes underwent method validation where Tables S7 and Table 1 dictate the instrument and method validation parameters, respectively.

During validation, of the 204 BCIs 12 had an r^2^ < 0.997; however, all remaining were > 0.990. The IDL varied from 0.0003 ng/mL for metoprolol to 2.4 ng/mL for sucralose. The IQL varied from 0.001 ng/mL for metoprolol to 7.2 ng/mL, again for sucralose. 190 of 204 BCIs had an accuracy (%) of 50 < x < 120, and only 2 BCIs had a precision > 30%, TMAO and imatinib. The compounds which fell outside the range for accuracy and precision are deemed to be semi-quantitative in instrument performance. Poor instrument performance for these 14 analytes could be due to a number of factors varying from co-elution to poor peak shape. Imatinib observed a broad peak with extensive tailing, resulting in challenging integration, a potential cause for this large variability. On the other hand, polar compounds with limited retention in reverse-phase chromatography such as TMAO can often have poor precision, especially when there is no suitable internal standard. Poor retention can also limit accuracy, in instances with poorly matched internal standards; guanyl urea and asymmetric dimethyl arginine both have this same effect. Heroin saw very low accuracy, and structurally related opioid ISTDs such as codeine and morphine were trialled; however, these only saw minor improvements when compared to those with a similar retention time. Additionally, this method is unable to distinguish between isomers — dexamethasone and betamethasone— they are reported as the total sum of both compounds. A complete knowledge of analyte suitability can be understood via the method performance, which is found in Table 1.

**Table 1:** Method performance of all analytes in influent wastewater, following the sample procedure detailed in Sect."Materials and methods", where RT = retention time, RRT = relative retention time, MDL = method detection limit and MQL = method quantification limit. (MDMA = 3,4 – Methylenedioxymethamphetamine, EDDP = 2-ethylidene-1,5-dimethyl-3,3-diphenylpyrrolidine)

Class	Compound	ISTD	ESI mode	Method Performance							
				RT (min)	RRT	SPE cartridge	Recovery (%)	MDL/ngL^−1^	MQL/ngL^−1^	Accuracy (%)	Precision (%)
Illicit drugs	4-methylmethcathinone	Ketamine-d4	ESI +	10.11 ± 0.04	1.18 ± 0.004	MCX (E2)	126.55 ± 4.04	0.4	1.1	90.7	10.0
	Amphetamine	Amphetamine-d5	ESI +	8.81 ± 0.07	1.01 ± 0.01	HLB	103.40 ± 8.80	0.2	0.5	100.4	10.9
	Cocaine	Cocaine-d3	ESI +	11.42 ± 0.03	1.00 ± 0.02	MCX (E2)	94.96 ± 3.53	0.02	0.1	102.6	4.1
	Anhydroecgonine methylester	Cotinine-d3	ESI +	3.33 ± 0.09	1.00 ± 0.02	MCX (E2)	48.02 ± 0.69	0.02	0.1	101.0	1.8
	Benzoylecgonine	Benzoylecgonine-d8	ESI +	10.45 ± 0.20	0.99 ± 0.12	HLB	101.50 ± 3.90	0.1	0.4	95.7	2.9
	Cocaethylene	Cocaethylene-d3	ESI +	12.98 ± 0.02	1.00 ± 0.001	HLB	104.20 ± 1.20	0.01	0.02	99.1	3.6
	Heroin	Codeine-d6	ESI +	11.03 ± 0.05	3.35 ± 0.05	HLB	14.70 ± 0.83	8.9	26.9	104.0	5.1
	6-monoacetylmorphine	Ketamine-d4	ESI +	7.23 ± 0.06	0.7 ± 0.004	MCX (E2)	90.80 ± 5.58	0.1	0.3	104.3	7.7
	Ketamine	Ketamine-d4	ESI +	10.60 ± 0.06	1.02 ± 0.02	HLB	101.70 ± 2.50	0.02	0.1	99.4	4.8
	Norketamine	Norketamine-d4	ESI +	10.63 ± 0.07	1.00 ± 0.01	HLB	91.40 ± 1.20	0.04	0.1	100.2	1.9
	Methamphetamine	Methamphetamine-d5	ESI +	9.04 ± 0.05	1.00 ± 0.002	HLB	92.20 ± 5.40	0.03	0.1	101.8	5.9
	3,4 – Methylenedioxyamphetamine	MDMA-d5	ESI +	8.90 ± 0.04	1.01 ± 0.001	MCX (E2)	115.40 ± 9.00	0.1	0.3	105.5	10.0
	MDMA	MDMA-d5	ESI +	9.18 ± 0.05	1.00 ± 0.002	HLB	85.50 ± 3.50	0.1	0.4	98.7	8.0
	Methylenedioxypyrovalerone	Benzoylecgonine-d8	ESI +	12.06 ± 0.03	1.16 ± 0.002	MCX (E2)	99.74 ± 5.33	0.01	0.0	103.8	6.8
Lifestyle chemicals	Caffeine	Caffeine-d9	ESI +	9.31 ± 0.04	1.01 ± 0.002	HLB	92.60 ± 5.90	0.9	2.8	98.7	7.9
	7-methylxanthine	Caffeine-d9	ESI +	3.64 ± 0.05	0.40 ± 0.01	MCX (E1)	5.36 ± 0.12	5.4	16.4	101.6	1.3
	Paraxanthine	Caffeine-d9	ESI +	6.43 ± 0.07	0.70 ± 0.01	HLB	119.40 ± 1.20	0.7	2.2	99.2	2.4
	Nicotine	Cotinine-d3	ESI +	3.12 ± 0.05	0.91 ± 0.003	HLB	15.00 ± 0.14	4.7	14.2	100.6	1.7
	Cotinine	Cotinine-d3	ESI +	3.37 ± 0.03	1.00 ± 0.01	HLB	90.80 ± 2.10	0.02	0.1	97.4	2.7
Pharmaceuticals	17α-Ethinylestradiol	Estradiol-d4	ESI-	9.53 ± 0.08	0.87 ± 0.36	HLB	79.06 ± 3.14	2.7	8.2	102.8	14.1
	Acetaminophen	Acetaminophen-d4	ESI +	5.13 ± 0.02	1.01 ± 0.004	MCX (E1)	93.26 ± 3.86	0.5	1.4	97.1	18.0
	Amitriptyline	Amitriptyline-d3	ESI +	17.04 ± 0.03	1.00 ± 0.002	HLB	98.60 ± 4.10	0.1	0.2	103.7	4.6
	Amlodipine	Sertraline-d3	ESI +	17.42 ± 0.03	0.96 ± 0.001	MCX(E2)	113.16 ± 5.88	0.01	0.03	96.3	9.9
	O-des[2-aminoethyl]O-carboxymethyl dehydroamlodipine	Carbamazepine 13C6	ESI +	17.56 ± 0.02	1.09 ± 0.001	MCX (E1)	104.99 ± 1.75	0.4	1.1	98.8	3.8
	Atenolol	Atenolol-d7	ESI +	4.02 ± 0.07	1.01 ± 0.02	HLB	90.60 ± 2.70	0.2	0.5	98.1	7.4
	Atorvastatin	Ibuprofen-d3	ESI-	9.66 ± 0.03	0.93 ± 0.01	HLB	116.80 ± 4.20	0.4	1.2	102.6	5.2
	2-OH Atorvastatin	Ibuprofen-d3	ESI-	9.34 ± 0.03	0.89 ± 0.004	HLB	123.80 ± 5.80	0.2	0.6	103.3	7.6
	Beclomethasone	Cortisol-d4	ESI +	17.88 ± 0.20	1.08 ± 0.003	HLB	162.32 ± 9.09	0.1	0.2	96.0	18.0
	Betamethasone/Dexamethasone	Cortisol-d4	ESI +	17.63 ± 0.21	1.07 ± 0.002	HLB	108.25 ± 4.32	0.1	0.2	97.2	7.5
	Bendroflumethiazide	Estrone-d4	ESI-	7.76 ± 0.02	0.82 ± 0.03	HLB	87.60 ± 0.75	1.1	3.3	100.6	11.3
	Bezafibrate	Bezafibrate-d6	ESI-	8.44 ± 0.04	1.00 ± 0.002	HLB	105.20 ± 0.20	0.4	1.1	99.9	4.3
	Bisoprolol	Carbamazepine 13C6	ESI +	13.46 ± 0.03	0.84 ± 0.001	MCX (E2)	66.59 ± 1.77	0.1	0.2	101.9	3.1
	Budesonide	Cortisol-d4	ESI +	20.08 ± 0.14	1.21 ± 0.01	HLB	87.80 ± 3.55	0.1	0.3	97.1	14.9
	Buprenorphine	Cocaine-d3	ESI +	14.70 ± 0.04	1.29 ± 0.001	MCX (E2)	97.55 ± 0.45	0.1	0.3	99.7	1.9
	Capecitabine	Desmethyl Diazepam-d5	ESI +	16.11 ± 0.04	0.87 ± 0.02	HLB	93.40 ± 8.90	0.02	0.1	98.8	12.7
	Carbamazepine	Carbamazepine 13C6	ESI +	16.10 ± 0.08	1.00 ± 0.02	HLB	91.30 ± 2.70	0.03	0.01	98.9	4.8
	Carbamazepine-10,11-epoxide	Carbamazepine 13C6	ESI +	13.82 ± 0.11	1.55 ± 0.02	HLB	196.77 ± 11.8	0.8	2.5	100.5	15.0
	10,11-dihydro-10-hydroxy carbamazepine	Codeine-d6	ESI +	13.82 ± 0.12	2.82 ± 0.04	HLB	85.68 ± 1.04	1.5	4.4	99.1	8.6
	Cimetidine	Atenolol-d7	ESI +	4.41 ± 0.02	1.13 ± 0.004	MCX (E2)	70.81 ± 3.32	0.2	0.7	96.7	7.1
	Citalopram	Citalopram-d6	ESI +	14.67 ± 0.02	1.00 ± 0.01	HLB	86.50 ± 3.70	0.1	0.02	95.3	4.0
	Desmethyl citalopram	Citalopram-d6	ESI +	14.78 ± 0.02	1.01 ± 0.01	HLB	93.00 ± 0.70	0.02	0.01	99.8	3.0
	Clobazam	Carbamazepine 13C6	ESI +	16.95 ± 0.03	1.05 ± 0.001	MCX (E1)	97.48 ± 3.49	0.01	0.03	102.5	7.0
	Codeine	Codeine-d6	ESI +	5.05 ± 0.07	1.02 ± 0.01	HLB	92.00 ± 8.50	0.1	0.4	90.4	8.9
	Norcodeine	Codeine-d6	ESI +	5.50 ± 0.04	1.12 ± 0.01	HLB	94.11 ± 1.91	0.6	1.8	101.4	7.5
	Dihydrocodeine	Codeine-d6	ESI +	4.80 ± 0.07	0.97 ± 0.01	HLB	85.80 ± 8.40	0.1	0.4	100.3	9.9
	Diazepam	Diazepam-d5	ESI +	18.92 ± 0.02	1.00 ± 0.001	MCX (E2)	105.09 ± 5.93	0.02	0.1	104.0	7.3
	Desmethyl diazepam	Diazepam-d5	ESI +	18.48 ± 0.02	0.98 ± 0.002	MCX (E2)	124.26 ± 0.55	0.1	0.2	99.7	2.7
	Diclofenac	Naproxen-d3	ESI-	9.06 ± 0.02	1.14 ± 0.003	HLB	140.25 ± 4.14	0.2	0.6	102.1	13.3
	4-hydroxy diclofenac	Naproxen-d3	ESI-	7.78 ± 0.02	0.98 ± 0.01	HLB	64.90 ± 2.16	1.4	4.3	97.6	18.5
	Diltiazem	Cocaethylene-d3	ESI +	15.58 ± 0.22	1.20 ± 0.01	HLB	69.41 ± 3.22	0.01	0.04	96.7	7.8
	N-desmethyl diltiazem	Quetiapine-d8	ESI +	15.67 ± 0.02	1.03 ± 0.001	HLB	62.90 ± 3.30	0.1	0.2	101.1	5.8
	Donepezil	Methadone-d9	ESI +	13.49 ± 0.03	0.81 ± 0.001	MCX (E2)	53.81 ± 5.75	0.2	0.5	107.6	6.7
	Duloxetine	EDDP-d3	ESI +	17.16 ± 0.43	1.18 ± 0.02	HLB	58.90 ± 7.05	0.1	0.3	108.5	10.2
	Ephedrine	Cocaine-d3	ESI +	6.30 ± 0.06	0.55 ± 0.004	MCX (E2)	85.78 ± 4.28	0.1	0.4	103.5	6.5
	Fexofenadine	Ibuprofen-d3	ESI-	8.52 ± 0.10	0.82 ± 0.01	HLB	67.30 ± 0.60	3.1	9.5	99.4	1.3
	Fluoxetine	Sertraline-d3	ESI +	17.40 ± 0.03	0.96 ± 0.001	HLB	86.60 ± 6.30	0.03	0.01	106.9	12.7
	Norfluoxetine	Sertraline-d3	ESI +	17.53 ± 0.03	0.97 ± 0.001	MCX (E2)	140.32 ± 2.09	0.02	0.1	99.0	3.4
	Gabapentin	Gabapentin-d4	ESI +	8.56 ± 0.12	1.01 ± 0.004	HLB	101.40 ± 7.80	0.2	0.7	105.4	12.8
	Gemfibrozil	Cortisol-d4	ESI +	22.66 ± 0.03	1.37 ± 0.001	MCX (E1)	99.00 ± 16.70	0.02	0.01	100.1	17.2
	Gliclazide	Diazepam-d5	ESI +	17.79 ± 0.02	0.95 ± 0.01	HLB	78.28 ± 9.51	1.1	3.4	91.4	11.6
	OH-Gliclazide	Metoprolol-d7	ESI +	14.69 ± 0.04	1.28 ± 0.003	HLB	105.40 ± 3.60	0.1	0.01	103.8	10.1
	Ibuprofen^‡^	Ibuprofen-d3	ESI-	10.44 ± 0.08	1.00 ± 0.001	HLB	94.80 ± 1.80	0.2	0.5	98.6	4.9
	2-OH ibuprofen^‡^	Estrone-d4	ESI-	7.70 ± 0.06	0.78 ± 0.01	HLB	46.30 ± 13.80	1.6	4.7	121.1	18.6
	Ifosfamide	Carbamazepine 13C6	ESI +	13.21 ± 0.03	0.82 ± 0.001	MCX (E1)	100.02 ± 3.05	0.01	0.02	102.2	4.4
	Imatinib	Amitriptyline-d3	ESI +	14.57 ± 0.80	0.77 ± 0.25	HLB	73.30 ± 0.78	1.5	4.6	100.8	42.1
	Irbesartan	Bezafibrate-d6	ESI-	8.67 ± 0.01	1.08 ± 0.003	HLB	98.57 ± 4.66	0.8	2.5	96.7	8.8
	Ketoprofen	Bezafibrate-d6	ESI-	7.78 ± 0.02	0.99 ± 0.03	HLB	122.69 ± 4.03	1.1	3.5	97.7	12.1
	Dihydroketoprofen	Bezafibrate-d6	ESI-	7.50 ± 0.03	0.88 ± 0.25	HLB	92.03 ± 4.28	1.3	4.1	103.3	11.9
	Lansoprazole	Desmethyl Diazepam-d5	ESI +	16.39 ± 0.19	0.89 ± 0.004	HLB	81.44 ± 0.77	0.2	0.6	100.7	17.2
	5-OH Lansoprazole	Propranolol-d7	ESI +	13.80 ± 0.09	0.94 ± 0.01	HLB	99.50 ± 7.08	0.6	1.8	108.1	11.2
	Lansoprazole Sulfone	Diazepam-d5	ESI +	16.58 ± 0.02	0.88 ± 0.01	MCX (E2)	37.31 ± 1.41	0.1	0.2	102.7	2.7
	Levetiracetam	Ketamine-d4	ESI +	6.21 ± 0.06	0.59 ± 0.07	HLB	91.80 ± 7.50	0.5	1.4	94.2	13.2
	Lisinopril	Quetiapine-d8	ESI +	9.70 ± 0.04	0.86 ± 0.002	MCX (E2)	70.40 ± 6.79	0.1	0.3	93.2	7.7
	Methadone	Methadone-d9	ESI +	16.71 ± 0.02	1.00 ± 0.001	HLB	88.50 ± 2.80	0.01	0.003	98.8	3.6
	EDDP	EDDP-d3	ESI +	14.51 ± 0.02	1.00 ± 0.001	HLB	95.30 ± 2.70	0.01	0.003	101.2	3.9
	Memantine	Amphetamine-d5	ESI +	15.18 ± 0.04	1.74 ± 0.02	HLB	95.40 ± 3.60	0.02	0.10	105.3	10.9
	Metformin*	Methamphetamine-d5	ESI +	2.46 ± 0.01	0.28 ± 0.002	MCX (E2)	100*	1.2	3.6	149.2	24.7
	Guanyl Urea	Amphetamine-d5	ESI +	2.82 ± 0.10	0.82 ± 0.03	MCX (E2)	8.07 ± 0.32	1.0	3.1	102.8	2.8
	Methotrexate	Quetiapine-d8	ESI +	9.36 ± 0.02	0.62 ± 0.002	MCX (E2)	51.54 ± 13.44	0.5	1.5	81.6	14.6
	Methyl prednisolone	Carbamazepine 13C6	ESI +	17.94 ± 0.04	1.11 ± 0.002	MCX (E1)	106.44 ± 0.03	0.2	0.7	100.0	7.1
	Metoprolol	Metoprolol-d7	ESI +	11.34 ± 0.04	1.00 ± 0.001	MCX (E2)	118.34 ± 1.30	0.003	0.01	100.8	2.8
	O-desmethyl metoprolol	Metoprolol-d7	ESI +	7.60 ± 0.08	0.67 ± 0.01	MCX (E2)	128.30 ± 0.99	0.1	0.2	100.6	3.4
	Mirtazapine	Mirtazapine-d3	ESI +	11.55 ± 0.03	1.00 ± 0.001	HLB	91.10 ± 6.20	0.1	0.02	99.5	13.1
	Morphine	Morphine-d3	ESI +	3.28 ± 0.07	0.97 ± 0.17	HLB	102.00 ± 2.90	0.6	1.8	101.2	11.2
	Dihydromorphine	Cotinine-d3	ESI +	3.19 ± 0.03	0.96 ± 0.01	MCX (E2)	89.21 ± 0.02	0.8	2.5	100.0	1.9
	Normorphine	Morphine-d3	ESI +	3.30 ± 0.04	1.02 ± 0.01	MCX (E2)	102.63 ± 4.89	0.7	2.2	103.4	5.7
	Naproxen	Naproxen-d3	ESI-	8.83 ± 0.10	1.00 ± 0.001	HLB	118.50 ± 3.50	1.4	4.1	102.1	3.5
	O-desmethyl naproxen	Estrone-d4	ESI-	7.49 ± 0.10	0.75 ± 0.01	HLB	59.00 ± 0.30	26.1	79.2	99.7	2.7
	Nicorandil	Ketamine-d4	ESI +	7.13 ± 0.03	0.69 ± 0.004	MCX (E2)	96.18 ± 2.61	1.0	2.9	101.9	5.1
	Nitrazepam	Methadone-d9	ESI +	16.43 ± 0.02	0.99 ± 0.002	MCX (E2)	94.92 ± 0.75	0.1	0.2	100.6	3.7
	Norephedrine	Caffeine-d9	ESI +	5.49 ± 0.04	0.59 ± 0.20	MCX (E2)	67.92 ± 1.76	0.04	0.1	101.8	2.8
	Nortriptyline	Nortriptyline-d3	ESI +	17.34 ± 0.06	1.00 ± 0.004	HLB	91.50 ± 2.30	0.1	0.03	97.8	5.6
	10-OH nortriptyline	Citalopram-d6	ESI +	14.89 ± 0.02	1.02 ± 0.001	HLB	129.90 ± 4.50	0.01	0.003	100.2	14.0
	Oseltamivir	Desmethyl Diazepam-d5	ESI +	14.93 ± 0.05	0.81 ± 0.002	MCX (E2)	52.83 ± 2.33	0.02	0.1	103.1	5.3
	Oxazepam	Desmethyl Diazepam-d5	ESI +	17.59 ± 0.21	0.96 ± 0.002	HLB	96.90 ± 2.42	0.1	0.3	101.8	4.6
	Prednisolone	Cortisol-d4	ESI +	16.57 ± 0.21	1.00 ± 0.001	HLB	107.58 ± 10.27	0.1	0.3	93.3	20.6
	Pregabalin	Gabapentin-d4	ESI +	8.45 ± 0.10	0.99 ± 0.01	HLB	70.60 ± 7.50	1.6	4.7	112.2	12.4
	N-methyl pregabalin	Gabapentin-d4	ESI +	8.30 ± 0.07	1.01 ± 0.01	HLB	111.24 ± 6.34	0.4	1.2	104.0	11.9
	Propranolol	Propranolol-d7	ESI +	14.72 ± 0.02	1.01 ± 0.001	HLB	92.00 ± 1.40	0.02	0.01	99.3	12.9
	Quetiapine	Quetiapine-d8	ESI +	15.24 ± 0.02	1.00 ± 0.01	HLB	92.60 ± 3.20	0.02	0.01	99.6	4.9
	7-OH Quetiapine	Quetiapine-d8	ESI +	9.50 ± 0.02	0.63 ± 0.002	MCX (E2)	131.41 ± 8.87	0.01	0.05	104.8	9.8
	Ranitidine	Cotinine-d3	ESI +	4.03 ± 0.08	0.82 ± 0.02	HLB	39.14 ± 0.38	1.2	3.7	100.7	9.4
	Ranitidine N-oxide	Cocaethylene-d3	ESI +	4.49 ± 0.04	0.35 ± 0.01	HLB	119.51 ± 0.66	0.1	0.4	99.6	5.7
	Risperidone	Methadone-d9	ESI +	12.76 ± 0.03	0.77 ± 0.001	MCX (E2)	39.45 ± 0.86	0.1	0.2	101.5	1.3
	Salbutamol	Norketamine-d4	ESI +	4.07 ± 0.02	0.39 ± 0.002	HLB	98.40 ± 6.20	0.001	0.004	93.4	8.5
	Sertraline	Sertraline-d3	ESI +	18.09 ± 0.08	1.00 ± 0.004	HLB	172.27 ± 2.74	0.1	0.4	101.1	11.7
	Norsertraline	Sertraline-d3	ESI +	18.49 ± 0.03	1.02 ± 0.001	MCX (E2)	86.05 ± 15.51	2.7	8.3	112.8	31.8
	Sildenafil	Quetiapine-d8	ESI +	15.60 ± 0.03	1.03 ± 0.002	MCX (E2)	132.69 ± 4.36	0.02	0.04	97.7	7.5
	N-desmethyl sildenafil	Quetiapine-d8	ESI +	15.69 ± 0.03	1.04 ± 0.002	MCX (E2)	116.12 ± 0.35	0.1	0.2	99.8	1.5
	Simvastatin	Carbamazepine 13C6	ESI +	23.45 ± 0.03	1.46 ± 0.001	MCX (E1)	61.34 ± 0.50	0.03	0.1	99.4	4.5
	Sitagliptin	Quetiapine-d8	ESI +	11.97 ± 0.04	0.79 ± 0.01	HLB	135.50 ± 16.90	0.1	0.2	98.0	18.8
	Tamoxifen	Verapamil-d7	ESI +	20.61 ± 0.03	1.34 ± 0.002	MCX (E2)	19.44 ± 4.08	0.2	0.6	85.2	4.4
	Temazepam	Carbamazepine 13C6	ESI +	17.91 ± 0.02	1.11 ± 0.001	MCX (E1)	100.25 ± 3.08	0.02	0.1	102.2	4.4
	Topiramate	Naproxen-d3	ESI-	7.54 ± 0.01	0.94 ± 0.01	HLB	62.28 ± 0.74	0.5	1.4	99.2	10.7
	Tramadol	Ketamine-d4	ESI +	11.15 ± 0.06	1.05 ± 0.002	HLB	96.20 ± 4.80	0.004	0.01	96.4	10.8
	O-desmethyl tramadol	Benzoylecgonine-d8	ESI +	8.99 ± 0.06	0.83 ± 0.003	HLB	118.70 ± 3.40	0.1	0.02	101.5	4.2
	N-desmethyl tramadol	Ketamine-d4	ESI +	12.09 ± 0.03	1.20 ± 0.002	HLB	76.60 ± 0.30	0.03	0.01	100.4	12.0
	Valsartan	Bezafibrate-d6	ESI-	8.39 ± 0.08	1.00 ± 0.01	HLB	137.10 ± 5.70	2.0	6.2	97.1	9.7
	4-hydroxy valsartan	Acetaminophen-d4	ESI-	6.77 ± 0.02	1.35 ± 0.03	HLB	95.45 ± 12.94	1.5	4.7	90.4	19.8
	Venlafaxine	Ketamine-d4	ESI +	13.86 ± 0.04	1.22 ± 0.002	MCX (E2)	97.03 ± 4.27	1.3	4.0	96.9	5.1
	Desvenlafaxine	Mirtazapine-d3	ESI +	10.97 ± 0.03	0.97 ± 0.002	MCX (E2)	92.09 ± 9.83	0.6	1.7	92.5	18.9
	Verapamil	Verapamil-d7	ESI +	15.38 ± 0.03	1.00 ± 0.001	MCX (E2)	104.27 ± 2.43	0.002	0.01	101.7	2.8
	p-O-desmethyl verapamil	Verapamil-d7	ESI +	14.66 ± 0.04	0.96 ± 0.001	MCX (E2)	104.59 ± 3.28	0.02	0.1	102.2	4.5
	Zolpidem	Cocaine-d3	ESI +	12.03 ± 0.05	1.04 ± 0.001	HLB	100.88 ± 2.59	0.01	0.04	101.8	4.8
Human markers	1,4-methylimidazoleacetic acid	Cotinine-d3	ESI +	2.77 ± 0.02	0.83 ± 0.01	MCX (E2)	37.09 ± 0.57	0.6	1.9	101.1	2.0
	2’-deoxyguanosine^*^	Adenosine 13C5	ESI +	3.37 ± 0.10	1.02 ± 0.01	HLB	100*	21.8	65.7	92.9	0.7
	2'-deoxyinosine	Codeine-d6	ESI +	3.46 ± 0.03	0.71 ± 0.004	HLB	80.51 ± 1.58	0.3	0.8	101.4	20.8
	3-nitro-L-tyrosine	Gabapentin-d4	ESI +	5.02 ± 0.05	0.63 ± 0.01	MCX (E2)	116.79 ± 8.12	0.2	0.7	95.1	12.1
	3-chloro-L-tyrosine	Gabapentin-d4	ESI +	4.41 ± 0.05	0.43 ± 0.24	MCX (E2)	104.31 ± 8.45	0.9	2.7	94.3	9.8
	5-Hydroxymethyl-2'-deoxyuridine*	Carbamazepine 13C6	ESI +	3.21 ± 0.22	0.19 ± 0.05	MCX (E1)	100*	48.7	147.2	184.2	2.8
	5-hydroxyindole acetic acid*	Acetaminophen-d4	ESI +	8.30 ± 0.04	1.63 ± 0.01	MCX (E1)	222.95 ± 69.46	0.4	1.1	78.0	227.0
	5-methyl-2'-deoxycytidine	Codeine-d6	ESI +	3.05 ± 0.012	0.63 ± 0.004	MCX(E2)	115.72 ± 17.95	0.8	2.3	89.0	21.6
	8-hydroxyguanosine	Adenosine 13C5	ESI +	3.52 ± 0.18	1.02 ± 0.05	MCX (E1)	13.25 ± 0.02	2.1	6.3	100.0	1.9
	8-oxoguanine	Acetaminophen-d4	ESI +	3.53 ± 0.19	0.62 ± 0.23	MCX (E1)	1.10 ± 0.73	96.7	293.3	146.7	4.1
	Adenosine	Adenosine 13C5	ESI +	3.45 ± 0.02	1.00 ± 0.01	MCX(E2)	153.94 ± 21.33	0.2	0.5	109.8	41.0
	Androstenedione	Cocaethylene-d3	ESI +	18.53 ± 0.12	1.43 ± 0.002	HLB	96.00 ± 8.40	0.03	0.01	99.4	14.0
	Asymmetric dimethyl arginine*	Cotinine-d3	ESI +	2.61 ± 0.16	0.79 ± 0.05	MCX(E2)	100*	2.3	7.0	106.2	1.6
	Cortisol	Cortisol-d4	ESI +	16.55 ± 0.04	1.00 ± 0.001	MCX (E1)	100.60 ± 4.84	0.1	0.2	96.6	15.5
	Cortisone	Cortisol-d4	ESI +	15.85 ± 0.02	0.96 ± 0.001	HLB	79.60 ± 8.00	0.1	0.2	98.1	10.7
	Creatinine	Codeine-d6	ESI +	2.55 ± 0.014	0.53 ± 0.003	MCX (E2)	65.01 ± 11.93	0.04	0.1	113.0	17.6
	Deoxyadenosine	Cotinine-d3	ESI +	3.46 ± 0.02	0.88 ± 0.01	HLB	28.96 ± 1.66	6.9	20.8	95.9	7.4
	Dihydrobiopterin	Cotinine-d3	ESI +	2.98 ± 0.21	0.9 ± 0.06	MCX (E2)	88.08 ± 1.41	0.1	0.3	101.1	8.5
	Dihydrotestosterone	Cortisol-d4	ESI +	20.65 ± 0.03	1.25 ± 0.001	MCX (E1)	14.48 ± 8.20	2.7	8.2	140.1	20.9
	Estradiol	Estradiol-d4	ESI-	9.59 ± 0.03	1.02 ± 0.031	HLB	92.28 ± 0.23	3.8	11.4	100.2	8.8
	Estrone	Estrone-d4	ESI-	9.67 ± 0.03	1.00 ± 0.01	HLB	102.21 ± 5.16	0.6	1.9	103.6	9.7
	Formiminoglutamic acid	Cotinine-d3	ESI +	2.76 ± 0.05	0.83 ± 0.02	MCX (E2)	6.03 ± 3.25	5.8	17.6	138.1	9.0
	Hippuric acid	Gabapentin-d4	ESI +	9.03 ± 0.04	1.06 ± 0.01	HLB	152.30 ± 2.78	0.1	0.2	101.1	4.5
	4-Hydroxy-2-nonenal mercapturic acid	Bezafibrate-d6	ESI-	7.45 ± 0.03	0.88 ± 0.01	HLB	65.90 ± 0.70	2.1	6.5	100.8	3.7
	Hydroxymethyl uracil*	Cotinine-d3	ESI +	2.67 ± 0.08	0.81 ± 0.02	MCX (E2)	100	3.2	9.7	84.9	19.4
	Indoxyl sulfate	Acetaminophen-d4	ESI-	6.01 ± 0.13	1.16 ± 0.03	HLB	89.60 ± 2.10	7.4	22.5	101.6	7.0
	Inosine	Codeine-d6	ESI +	3.37 ± 0.03	0.69 ± 0.01	HLB	25.17 ± 7.37	0.8	2.5	120.7	19.8
	Nε-(1-Carboxyethyl)-L-lysine	Cotinine-d3	ESI +	2.58 ± 0.03	0.78 ± 0.01	MCX (E2)	26.08 ± 5.41	4.6	13.8	85.3	5.9
	Nε-(1-Carboxymethyl)-L-lysine	Cotinine-d3	ESI +	2.71 ± 0.12	0.82 ± 0.04	MCX (E2)	50.88 ± 0.82	0.7	2.0	92.7	1.9
	Neopterin	Cotinine-d3	ESI +	3.19 ± 0.02	0.96 ± 0.01	MCX (E2)	17.50 ± 5.04	6.3	19.0	120.4	14.6
	Phenyl acetyl glutamine	Caffeine-d9	ESI +	9.13 ± 0.03	1.01 ± 0.002	MCX (E1)	74.26 ± 1.54	0.4	1.1	101.5	6.1
	Pterin	Cotinine-d3	ESI +	3.38 ± 0.02	1.02 ± 0.01	MCX (E2)	87.98 ± 3.70	0.4	1.2	97.0	7.0
	Progesterone	Cocaine-d3	ESI +	21.61 ± 1.87	1.87 ± 0.02	HLB	42.30 ± 7.64	0.04	0.10	98.1	9.6
	Pyroglutamic acid	Cotinine-d3	ESI +	2.82 ± 0.02	0.72 ± 0.01	MCX (E2)	17.13 ± 4.11	6.4	19.4	83.0	4.7
	Testosterone	Cortisol-d4	ESI +	19.27 ± 0.02	1.17 ± 0.001	HLB	90.50 ± 6.50	0.1	0.02	94.8	7.3
Food	1-methyl-2-pyridone-5-carboxamide	Cotinine-d3	ESI +	3.63 ± 0.06	1.10 ± 0.23	MCX (E1)	7.93 ± 2.64	5.5	16.6	123.5	4.7
	1-methylhistidine	Amphetamine-d5	ESI +	2.87 ± 0.35	0.33 ± 0.11	MCX (E2)	11.57 ± 2.08	0.4	1.2	87.3	3.8
	3-carboxy-4-methyl-5-propyl-2-furanpropanoic acid	Carbamazepine 13C6	ESI +	18.87 ± 0.03	1.17 ± 0.001	MCX (E1)	13.40 ± 0.17	30.5	92.3	98.5	1.2
	3-methylhistidine	Amphetamine-d5	ESI +	2.47 ± 0.04	0.28 ± 0.012	MCX (E2)	36.44 ± 3.63	0.2	0.7	93.0	5.4
	4-Pyridoxic acid	Cotinine-d3	ESI +	3.71 ± 0.05	1.05 ± 0.03	HLB	92.67 ± 0.86	1.0	3.0	100.7	2.7
	5-(3′,4′-Dihydroxyphenyl)-γ-valerolactone	Caffeine-d9	ESI +	9.66 ± 0.03	1.07 ± 0.001	MCX (E1)	193.49 ± 1.07	0.3	1.0	100.4	5.2
	Acesulfame K	Bezafibrate-d6	ESI-	3.40 ± 0.04	0.40 ± 0.01	HLB	11.80 ± 0.20	9.4	28.3	98.8	1.7
	a-CEHC	Acetaminophen-d4	ESI-	8.67 ± 0.09	1.68 ± 0.02	HLB	108.60 ± 1.30	0.6	1.8	100.9	3.7
	Aspartame	Cocaine-d3	ESI +	11.13 ± 0.03	0.97 ± 0.001	MCX (E2)	103.11 ± 6.49	0.1	0.2	95.6	8.0
	Carnitine	Cotinine-d3	ESI +	2.42 ± 0.01	0.62 ± 0.003	MCX (E2)	1.67 ± 0.17	142.0	429.9	107.4	0.3
	Daidzein	Methylparaben 13C6	ESI-	7.86 ± 0.02	1.03 ± 0.003	HLB	118.47 ± 30.34	2.3	7.0	118.1	45.0
	Enterodiol	Naproxen-d3	ESI-	7.91 ± 0.02	0.90 ± 0.01	HLB	86.00 ± 2.30	4.8	14.7	101.9	6.0
	Enterolactone	Methylparaben 13C6	ESI-	8.17 ± 0.02	1.05 ± 0.002	HLB	91.10 ± 2.60	1.0	2.9	102.0	6.3
	Epicatechin	Estrone-d4	ESI-	6.84 ± 0.18	0.73 ± 0.01	HLB	16.73 ± 6.71	22.8	69.0	128.4	16.4
	Equol	Methylparaben 13C6	ESI-	8.13 ± 0.02	1.07 ± 0.01	HLB	81.79 ± 9.49	2.8	8.6	108.2	11.6
	Ferulic Acid	Ibuprofen-d3	ESI-	6.64 ± 0.02	0.64 ± 0.01	HLB	18.20 ± 1.10	7.6	22.9	95.9	2.5
	Genistein	Bezafibrate-d6	ESI-	8.35 ± 0.03	1.04 ± 0.004	HLB	32.57 ± 4.53	8.6	26.2	90.2	10.5
	Glycitein	Methylparaben 13C6	ESI-	7.88 ± 0.01	1.04 ± 0.002	HLB	86.05 ± 8.72	3.9	11.8	107.2	14.8
	Histidine	Codeine-d6	ESI +	2.54 ± 0.07	0.53 ± 0.01	MCX(E2)	21.40 ± 7.54	1.7	5.2	75.1	8.6
	Lysine	Methamphetamine-d5	ESI +	2.48 ± 0.02	0.28 ± 0.002	MCX(E2)	34.33 ± 9.97	8.0	24.1	34.3	10.0
	Methionine	Metoprolol-d7	ESI +	3.20 ± 0.05	0.28 ± 0.004	MCX(E2)	33.91 ± 3.23	3.5	10.6	106.7	5.4
	N-methylnicotinamide	Cotinine-d3	ESI +	3.71 ± 0.01	0.99 ± 0.001	MCX(E2)	97.71 ± 2.29	0.1	0.2	101.7	8.7
	Pantothenic acid	Acetaminophen-d4	ESI-	3.21 ± 0.02	0.64 ± 0.01	HLB	7.13 ± 3.08	12.7	38.5	130.6	4.4
	Phenylalanine	Gabapentin-d4	ESI +	4.76 ± 0.03	0.60 ± 0.01	MCX (E2)	96.79 ± 11.87	1.6	4.7	108.7	19.0
	Phloretin*	Naproxen-d3	ESI-	9.39 ± 0.36	1.02 ± 0.04	HLB	1.38 ± 0.77	860.8	2608.2	139.8	0.8
	Resveratrol	Estradiol-d4	ESI-	7.84 ± 0.09	0.83 ± 0.02	HLB	55.28 ± 17.18	29.8	90.2	122.0	20.6
	Riboflavin	Caffeine-d9	ESI +	10.52 ± 0.03	1.16 ± 0.001	MCX (E1)	80.14 ± 3.01	0.2	0.6	97.3	12.6
	Saccharin	Bezafibrate-d6	ESI-	4.34 ± 0.06	0.52 ± 0.01	HLB	22.00 ± 2.30	74.3	225.0	106.3	4.2
	Stachydrine	Cotinine-d3	ESI +	2.80 ± 0.05	0.84 ± 0.02	MCX (E2)	3.99 ± 0.47	7.9	23.8	108.3	0.7
	Sucralose	Naproxen-d3	ESI-	6.80 ± 0.01	0.77 ± 0.01	HLB	103.00 ± 9.70	23.4	70.9	106.1	18.1
	D,L-Sulforaphane N-acetyl L-cysteine	Diazepam-d5	ESI +	9.42 ± 0.04	0.50 ± 0.004	HLB	19.80 ± 0.60	0.1	0.4	102.0	3.6
	Trimethylamine N-oxide	Atenolol-d7	ESI +	2.69 ± 0.02	0.69 ± 0.01	MCX (E2)	8.18 ± 0.86	2.4	7.2	92.6	2.5
	Tryptophan	Gabapentin-d4	ESI +	7.19 ± 0.07	0.91 ± 0.01	MCX (E2)	89.78 ± 11.36	0.1	0.2	91.1	15.4
	Urolithin A	Methylparaben 13C6	ESI-	7.93 ± 0.04	1.04 ± 0.01	HLB	112.88 ± 20.96	5.8	17.4	113.1	27.1
	Valine	Amphetamine-d5	ESI +	2.80 ± 0.02	0.31 ± 0.002	MCX (E2)	35.67 ± 14.49	1.3	3.9	128.7	21.5
Personal care products	3-hydroxypropyl mercapturic acid	Codeine-d6	ESI +	4.15 ± 0.06	0.85 ± 0.01	HLB	58.10 ± 4.85	1.1	3.2	105.9	16.2
	Benzophenone-1	Methylparaben 13C6	ESI-	9.33 ± 0.01	1.23 ± 0.004	HLB	84.44 ± 6.79	1.0	3.1	94.3	8.3
	Benzophenone-2	Estrone-d4	ESI-	7.83 ± 0.01	0.82 ± 0.01	HLB	91.93 ± 4.74	13.4	40.6	103.6	9.8
	Benzophenone-4	Naproxen-d3	ESI-	7.25 ± 0.02	0.82 ± 0.01	HLB	95.10 ± 1.30	2.3	7.0	99.0	5.4
	Bisphenol A	Ibuprofen-d3	ESI-	8.96 ± 0.01	0.92 ± 0.003	HLB	79.29 ± 17.97	1.0	3.0	116.0	27.4
	Chloroxylenol	Bisphenol A-d16	ESI-	10.22 ± 0.02	1.12 ± 0.003	HLB	78.80 ± 4.10	7.9	2.6	103.6	10.9
	Butylparaben	Methylparaben 13C6	ESI-	9.77 ± 0.02	1.29 ± 0.02	HLB	88.62 ± 5.83	0.1	0.2	95.4	5.9
	Ethylparaben	Methylparaben 13C6	ESI-	8.23 ± 0.01	1.08 ± 0.004	HLB	107.02 ± 0.46	0.4	1.3	100.3	10.5
	Methylparaben	Methylparaben 13C6	ESI-	7.76 ± 0.02	1.00 ± 0.003	HLB	100.70 ± 1.40	0.9	0.3	99.0	6.2
	Propylparaben	Methylparaben 13C6	ESI-	8.98 ± 0.02	1.18 ± 0.01	HLB	103.90 ± 2.59	0.3	1.0	101.8	13.2

*Poor recovery, assumed at 100%, semi-quantitative

Most compounds (141) had recoveries between 50 and 120%. Due to the unpredictable nature and the complexity of influent wastewater, recoveries of certain analytes were outside this range. The low recovery was especially the case for some of the polar compounds, where this was observed with the cartridges used within this workflow; an additional 40 compounds with consistent recovery (low variability) < 50 were also taken forward. The low recovery could be ascribed to variable matrix suppression or enhancement known to occur with the endogenous (3.4) and food (3.5) markers [23]. Utilising both HLB and MCX cartridges saw improvements in precision; however, improvements may be observed with more specialist cartridges or utilising direct injection sample preparation. Lastly, it is expected that improvements to recovery could be made with directly matched isotopic analogues; however, due to commercial availability and price, this is not possible.

### Illicit drugs as BCIs for drug abuse and lifestyle choices

**Fig. 2:**
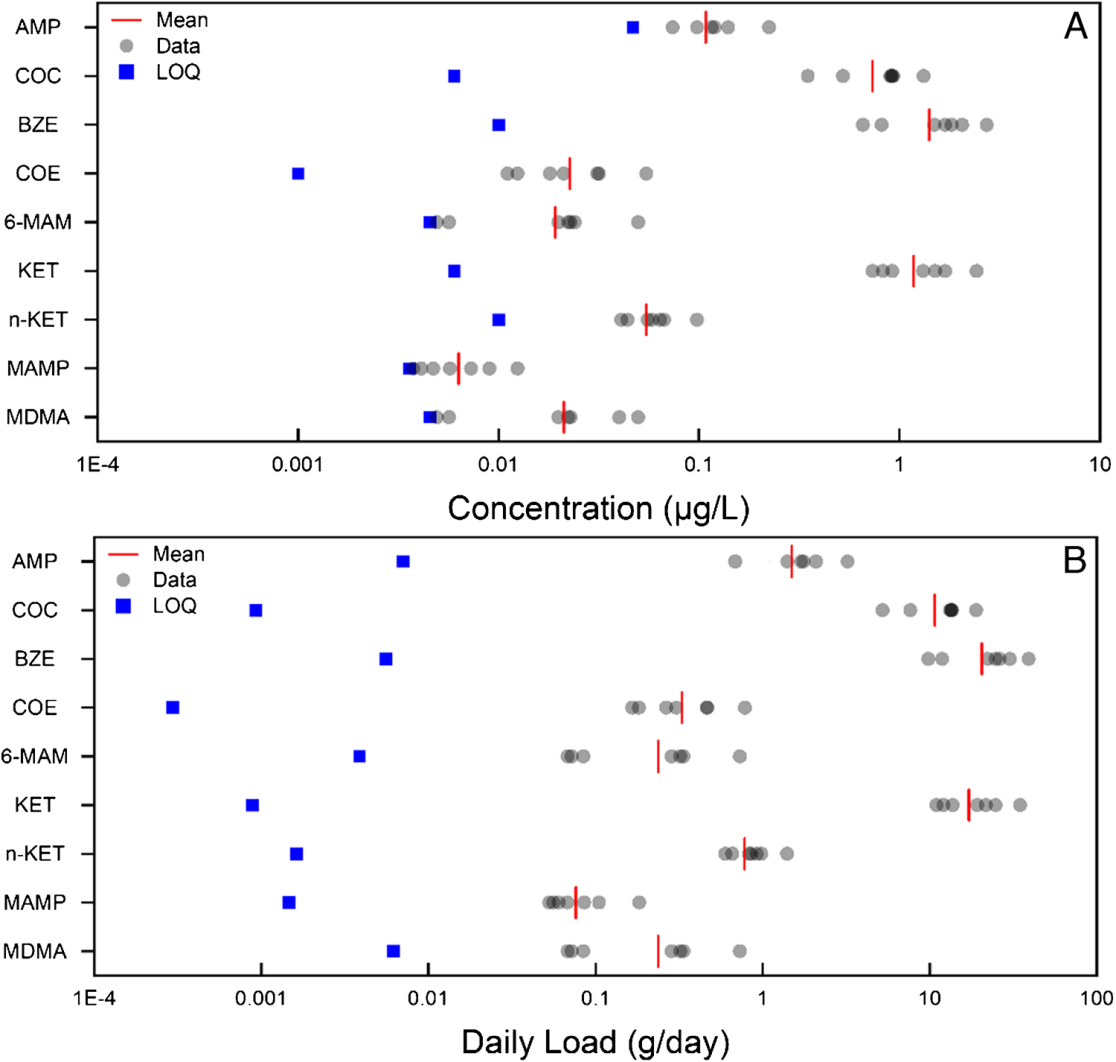
7-day temporal study on the ‘illicit drug’ BCIs, quantified in influent wastewater. Figure A details the wastewater concentration; LOQ is determined via the MQL (Table 1). Figure B is the daily load for the same samples. Here, daily load LOQ was calculated by multiplying the MQL by the average daily flow rate (L/day) across the 7-day sampling week. Abbreviations are defined within Table S1

One of the most commonly analysed small-molecule compound classes is illicit drugs, enabling an understanding of drug use at the community level. WBE has established clear links between drug use and other external factors such as the COVID-19 pandemic, levels of ‘urbanity’ or socioeconomic status. As a result, illicit drug use can have a direct reflection on the community health of a respective area. Incorporation of this compound class enables a possible understanding of substance use and lifestyle habits, providing critical insights towards the psyche of communities. The loads of illicit drugs detected within the week-long sampling campaign are displayed below in Fig. 2 with the respective minimum, median, average, and maximum across the week displayed in Table S8.

Of the 14 illicit drugs included within this method, 9 were detected in the samples, with benzoylecgonine (BZE, metabolite of cocaine (COC)) having the highest (weekly average) concentration of 1605.8 ± 712.2 ng/L, and methamphetamine (MAMP) having the lowest (weekly average) concentration of 5.9 ± 3.1 ng/L. When understanding the Daily load, the same weekly average was observed, where BZE had the largest mass load of 23.45 ± 10.17 g/day, whereas MAMP had the minimum (weekly average) at 0.09 ± 0.05 g/day. This method has been applied to samples where variable drug use occurs and is able to distinguish between the most commonly abused substances, enabling public health practitioners to target their support to specific regions, working towards the betterment of community health. This newly developed method identified similar trends in drug use when compared to previous results within the region; here, the same trend of increased illicit drug load was observed (BZE > KET > AMP > MDMA > MAMP) [11]. This method was also able to include 6-MAM to estimate heroin consumption. The diversification of illicit drugs within this method can increase the potential impact when deployed in future WBE studies.

### Caffeine and nicotine BCIs as indicators of community habits

Alongside illicit drugs, lifestyle chemical BCIs are also commonly analysed in WBE as nicotine (NIC) and caffeine (CAF) use is extremely prevalent. NIC and CAF use is usually understood by looking at a combination of their abundance vs the abundance of their metabolites. In addition to ‘double-authentication’ where the given compound is metabolised, confirming its consumption, in the case of cotinine (a metabolite of NIC) it also provides greater in-situ stability, giving a more representative indication of use [18].

CAF was also detected, alongside 2 of its metabolites, paraxanthine (PAX) and 7-methylxanthine (7-MEX), in line with similar methods in literature [44]. Here 7-MEX was in a marginally higher concentration which could have been due to additional sources of 7-MEX such as cocoa. These compounds can also be used in population assessments via normalisation [45]. A greater understanding of the origin of the metabolites by monitoring the parent:metabolite ratio has also shown great promise when highlighting moments of direct disposal in pharmaceutical analysis [46, 47]. Here, both 7-MEX and PAX had a parent:metabolite ratio of 0.35 ± 0.04 and 2.53 ± 0.70; paraxanthine was in line with previous ratios determined within this catchment [11].

The load of lifestyle chemicals detected within the week-long sampling campaign is displayed in Figure S4 with the respective minimum, median, average, and maximum across the week displayed in Table S8. Of the 5 Lifestyle chemicals under study within this manuscript, 100% were detected in all samples. All in high concentrations; however, 7-MEX had the largest daily load (weekly average) at 849.04 ± 249.02 g/day, whereas NIC had the minimum (weekly average) at 6.83 ± 3.67 g/day.

### Pharmaceuticals as proxies for disease

Understanding pharmaceutical use is critical because it can give an indication towards the human response to certain external stressors, such as pain treatment during the COVID-19 pandemic [13],or where the consumption of pharmaceuticals can act as a proxy for health and disease status within a community. It has also been used to understand various underlying conditions, such as depression [14] or gout [15]. Alongside pharmaceutical consumption, the analysis of pharmaceutical residues can also provide an understanding of community attitudes towards best practice of pharmaceutical disposal, via monitoring direct disposal [47]. This framework for a community health assessment aims to incorporate the ability to assess human response, by virtue of which pharmaceuticals have been administered, to a broad range of pharmaceutical classes.

**Fig. 3:**
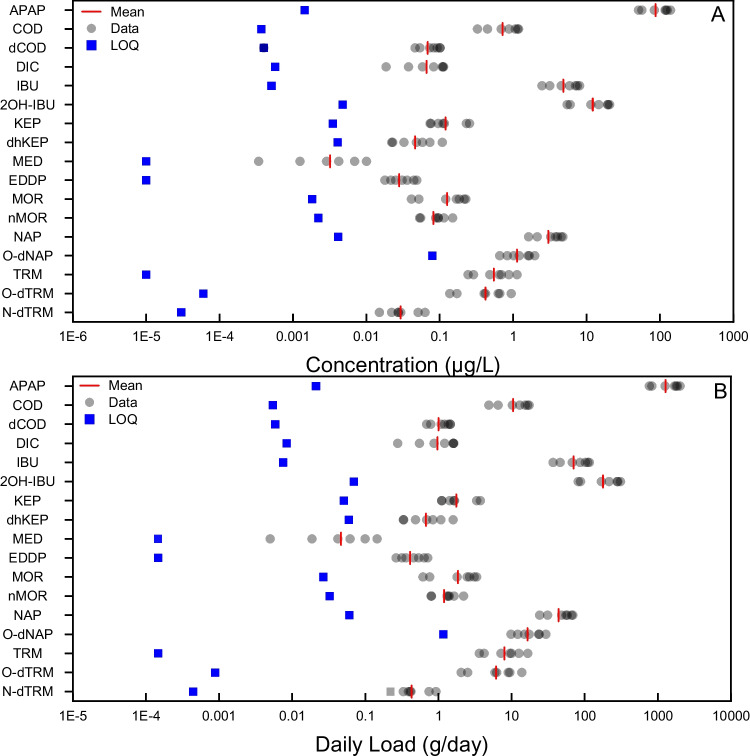
7-day temporal study on the pharmaceutical sub-class ‘pain’ BCIs, quantified in influent wastewater. Figure A details the wastewater concentration; LOQ is determined via the MQL (Table 1). Figure B is the daily load for the same samples. Here, daily load LOQ was calculated by multiplying the MQL by the average daily flow rate (L/day) across the 7-day sampling week. Abbreviations are defined within Table S1

Within this newly established framework, simultaneous determination of 77 different pharmaceuticals, including 29 parent/metabolite combinations, is conducted. Of all pharmaceuticals analysed in the method, 54% were quantified within the samples analysed with variable levels of load, indicated in Table S8. The pharmaceuticals were split into 14 different sub-categories, depending on the pharmaceutical type. Each figure details both the concentration and daily load of each pharmaceutical sub-class, including pain (Fig. 3), cardiovascular drugs (Figure S5), anxiety (Figure S6), cancer (Figure S7), anti-epileptics (Figure S8), ulcer (Figure S9), dementia (Figure S10), diabetes (Figure S11), stimulant (Figure S12), asthma/allergy (Figure S13). Other sub-classes, including anti-viral, GUD/ED, insomnia, and steroid hormones, which were detailed in Table S1, were not detected.

Figure 3 highlights the variable concentration and mass loads which occur across the week for pharmaceuticals within the pain sub-class. These trends can indicate temporal trends in pain or human response to these trends. Across all pharmaceuticals, acetaminophen (APAP) was found with the highest (weekly average) wastewater concentration 99.4 ± 35.4 μg/L and salbutamol (SAL) with the lowest (weekly average) wastewater concentration, 2.8 ± 1.6 ng/L, above the MQL, 0.04 ng/L. When normalising concentration to daily load, the same minimum and maximum were observed, where APAP had the highest daily load (weekly average) at 1453.50 ± 505.31 g/day whereas SAL had the minimum (weekly average) at 0.04 ± 0.02 g/day.

By analysing a broad range of pharmaceutical markers, it could be possible to elucidate population health status via WBE. For example, by simultaneously monitoring both cardiovascular and diabetes pharmaceuticals, insight can be gained towards the trends in cardiovascular and metabolic health within the community. Studying pharmaceutical consumption within WBE requires a clinical diagnosis and therefore may underestimate the true incidence of disease. WBE has the potential to uncover associations between these markers to understand key drivers of public health [49] where the distribution of healthcare is limited. Alongside this, it could alienate certain groups within developed communities who may not immediately seek certain healthcare options. Therefore, a more comprehensive and unbiased health assessment can occur via the monitoring of endogenous chemicals, as discussed in Sect."Endogenous BCIs as markers of health status". By including both pharmaceuticals and endogenous markers, a more holistic insight can be obtained. For example, increases in 1,4-methylimidazole acetic acid, an endogenous marker of histamine burden, have been correlated with increased use of fexofenadine, an antihistamine [49].

### Endogenous BCIs as markers of health status

As previously discussed in the introduction, improving the suite of endogenously formed biomarkers is crucial for the development of WBE as a tool to assess community Health. These compounds have variable MQLs from 0.01 ng/L for androstenedione to 293.3 ng/L for 8-oxoguanine, which can therefore limit potential detection rates (Table 1). New endogenous markers may also be helpful for other key research questions within WBE, such as in situ population normalisation.

During a week of analysis on influent wastewater, 20/35 human BCIs were quantifiable, again with varying daily load (g/day) across the week. The BCIs which have variable daily loads may require further investigation, as this variability could be due to a number of reasons, varying from alternative sources, fluxes in population within the town, variable changes in production due to physiological response or instability in wastewater. These results are displayed in Table S8. Health markers under study in this manuscript can be broadly categorised into 8 sub-groups: DNA-related, advanced glycation end products, stress related, hormones, deficiencies, pteridines, or general human.

Within this method, multiple hormone sub-classes, including androgens, estrogens, progesterone, and serotonin, were included, all of which have been previously detected in wastewater [50–52]. The inclusion of ion-exchange SPE cartridges saw an improvement in the recovery of 23/35 analytes within this sub-section, highlighting the importance of developing orthogonal sample preparation techniques. Stress biomarkers are key indicators to be captured within wastewater analysis due to the information which can be gained following their analysis. Oxidative stress or lipid peroxidation biomarkers are indicative of DNA damage which can highlight exposure to external stressors; increased concentrations are typically found under increased stress) therefore, monitoring both internal human response and exposure will provide comprehensive insight towards community Health. Only 50% of ‘stress’ BCIs were detected, where the low detection rate may limit applicability to future studies (Table S8, Figure S14).

**Fig. 4:**
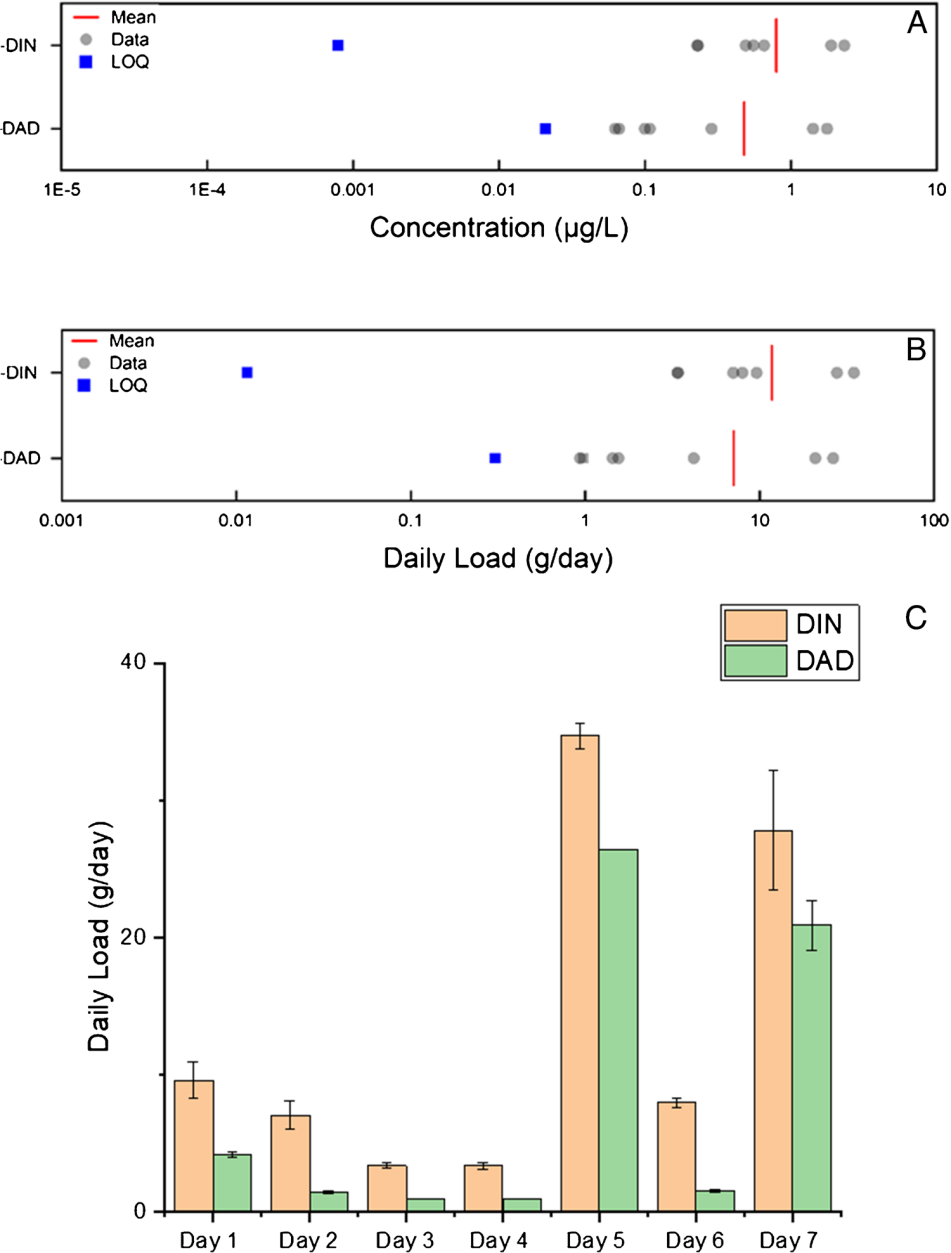
7-day temporal study on the health markers sub-class ‘DNA related’ BCIs, quantified in influent wastewater. Figure A details the wastewater concentration; LOQ is determined via the MQL (Table 1). Figure B is the daily load for the same samples. Here, daily load LOQ was calculated by multiplying the MQL by the average daily flow rate (L/day) across the 7-day sampling week. Abbreviations are defined within Table S1. Figure C details the inter-day variation of both 2-DIN and 2-DAD across one week (Day 1—Day 7, Mon-Sun)

Insights into DNA damage can be gained via the analysis of (modified) nucleosides [53, 54]. Examples of this are the 8-hydroxy-2-deoxyguanosine (oxidative damage) or deoxyinosine (2-DIN) which can be formed following nitrative damage of DNA after the deamination of deoxyadenosine (2-DAD) [55, 56] and during purine metabolism. Simultaneous measurements of both 2-DIN and 2-DAD enable accurate determination of the extent of nitrative damage or insights into other conditions such as acute kidney injury or chronic kidney disease [57]. There are lots of promising modified nucleosides; however, the lack of commercially available standards limits their applicability in this targeted MS approach used here [58]. Alongside modified nucleosides, nucleosides themselves, such as inosine, can also provide great insight into health status and occurrence of cancer [58]. Deoxyadenosine and deoxyinosine could provide valuable insights into adenosine deaminase status, which is linked with oxidative stress [59]. Additionally, it is hypothesised that increased purine nucleosides could be as a result of increased DNA turnover. Figure 4 below highlights the variability across the week, with a strong positive correlation observed between 2-DIN and 2-DAD (ρ = 0.96, p < 0.05).

It is important to note that some targets within this section, due to their nature as being DNA-related molecules, can also have non-human sources (via potential microbial degradation). Whilst it is possible for additional non-human contribution, the daily load data reported within this manuscript aligns closely to that reported in urine (estimated Daily urine volume is 1.57 L [60]) where wastewater load of 2-DIN and 2-DAD were 0.48 ± 0.45 mg/day/person and 0.29 ± 0.39 mg/day/person, respectively compared to 0.50 ± 0.16 mg/day/person and 0.18 ± 0.04 mg/day/person, respectively, in urine [57]. Future work to assess their utility will attempt to identify variable alternative sources and monitor variable loads reported throughout longitudinal monitoring.

Further classes, including pteridines, participate in multiple relevant biological functions that could provide valuable information of general community well-being [61]. Neopterin has associations with inflammation as a result of increased production during infectious diseases [62]; increased excretion of dihydrobiopterin and pterin has been observed in cancer patients [63]. Initially, tetrahydrobiopterin was included due to its critical role in homeostasis and neurotransmitter synthesis; however, due to poor stability, it was not possible to undergo analysis [64]. During the sample analysis, only pterin itself was detected (Figure S15).

Recent successes in WBE have been made possible via multi-biomarker correlation assessments and comparing WBE data to socio-economic metrics such as deprivation [34, 48]. The expansion of currently available endogenous data could improve these correlations. For example, exploring well-established links between food consumption and human health via advanced glycation end-products (AGEs), further possible insights can be gained via monitoring deficiencies, such as folate (measured via formiminoglutamic acid) [65]. AGEs are formed endogenously with proven associations to multiple chronic diseases, including diabetes, but their increased concentration can be associated with the heating of foods and hypercaloric diets [66–68]. Two major AGEs are Nε-(1-Carboxymethyl)-L-lysine (CEL) and Nε-(1-Carboxymethyl)-L-lysine (CML) both of which have been previously detected in urine in high concentrations [69]. Throughout the week, both CEL and CML were detected in high Daily loads, 103.33 ± 39.22 g/day and 94.62 ± 38.93, respectively (Figure S16). Allowing potential critical insight into AGE formation within the body. It is important to note that AGEs can also originate from food; however, this would still provide a great insight into community health via dietary patterns [70]. Beyond food, endogenous markers can enable correlations with pharmaceutical consumption too, such as pyroglutamic acid (Figure S17) also linked to chronic acetaminophen use; therefore, this correlation could also be further explored in WBE [71]. It is important to note that different compounds can cyclize in-source to pyroglutamic acid [72] therefore, it is possible this could also cause elevated mass loads detected in wastewater.

Alongside the seven sub-classes previously discussed, general human markers have also been included, which include creatinine (CRE), indoxyl sulfate (INDs), hippuric acid (HIPa), phenyacetyl glutamine (PhAG) and 1,4-methylimidazoleacetic acid (MIA). These are markers of muscle breakdown, uremic toxin – gut breakdown after consumption of tryptophan [73], uremic toxin – gut breakdown after consumption of phenylalanine [74, 75] and histamine burden [49]. Creatinine, commonly used for normalisation in urine analysis, is included to assist point-source normalisation (schools, hospitals etc.), where short hydraulic retention time limits the inherent instability previously shown in wastewater [76]. INDs, HIPa, and PhAG are dietary markers; however, due to their nature as a uremic toxin which can enable the progression of chronic kidney disease, they have been included here [77], rather than Sect."BCIs of food consumption".

Of the endogenous markers quantified (20/35), estrone was the lowest, 0.48 ± 0.18 g/day, whereas creatinine was the largest at 5836.47 ± 1697.14 g/day. Cortisol was not detected in all samples within the 7-day analysis (5/7), limiting a baseline understanding of wastewater concentrations. Overall, it is recommended that longitudinal monitoring and stability studies will be suitable to determine the suitability of these markers, enabling a greater understanding of community health in WBE.

### BCIs of food consumption

As discussed throughout, there are strong links between food and health; therefore, it is critical to have a greater understanding of food consumption at the population level because this can indicate stressors or changes in physiological response across seasons where different foods are consumed.

A key section is the consumption of artificial sweeteners, something which has been regularly studied in wastewater [31, 78, 79] can give indications of socioeconomic status [80]. However, there are known cancer risks for at least two artificial sweeteners, acesulfame-K and aspartame [81]. Alongside sweeteners, vitamins, lignans, and isoflavones are also under study, many of which have been detected in wastewater before [23] however, not alongside all pharmaceuticals and endogenous markers as previously mentioned. This method does introduce new gut metabolites, 5-(3′,4′-Dihydroxyphenyl)-γ-valerolactone (DHPV, gut metabolite of epicatechin) [82, 83]. A challenge with some gut metabolites is the frequent supplementation; in the case of polyphenols, this is common for urolithin A, also supplemented due to its anti-inflammatory properties [84, 85] Apart from polyphenols, other food compounds such as cruciferous vegetables have anti-cancer properties due to their isothiocyanate content [86]. This can be determined via measurement of sulforaphane N-acetyl cysteine [87].

Alongside food classes such as vitamins, amino acids also have critical importance towards health because of the role they play in metabolism [88]. More focus was placed on the amino acids which cannot be made within the body and therefore, must be consumed via food, often as meat; 3-methylhistidine was used here which drastically increases in urinary concentration following meat consumption [89–92]. Future work will be required to compare expected daily load in a WWTP to the quantified load to monitor increased load due to food waste; for example, therefore, gut metabolites are preferential markers due to the guarantee of consumption. The quantified daily loads of food targets are shown below in Fig. 7, in Figures S17-S24 and the values in Table S8.

**Fig. 5:**
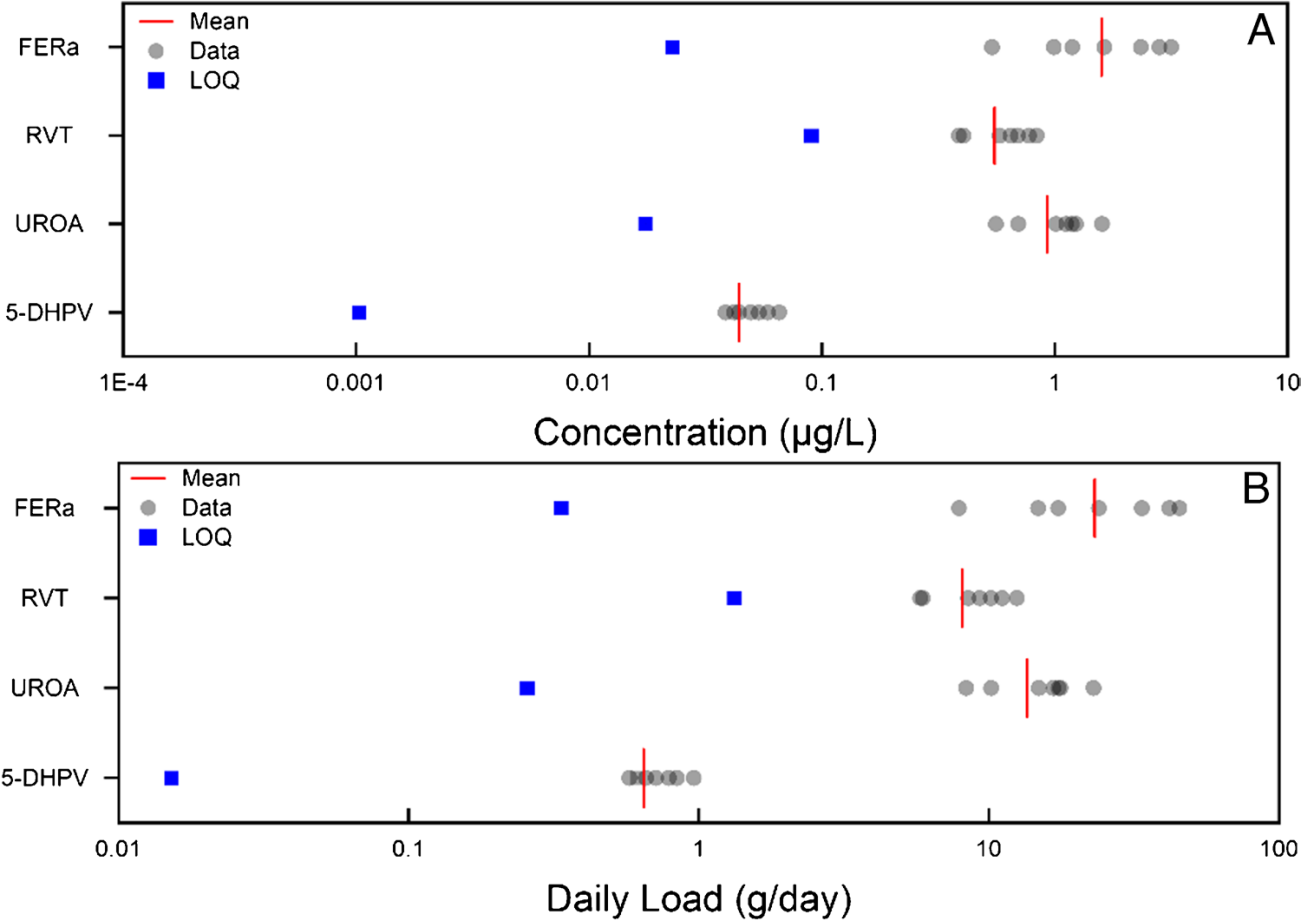
Day temporal study on the food markers sub-class ‘polyphenols BCIs, quantified in influent wastewater. Figure A details the wastewater concentration, LOQ is determined via the MQL (Table 1). Figure B is the daily load for the same samples. Here, daily load LOQ was calculated by multiplying the MQL by the average daily flow rate (L/day) across the 7-day sampling week. Abbreviations are defined within Table S1

Of the 35 food-related compounds which were implemented within this method, 29 were detected and quantified. These can be split into individual sub-categories, where the variability of concentration and Daily load across the 7-day period are displayed in the following figures: polyphenols (Fig. 5), vitamins (Figure S18), lignan (Figure S19), alkaloid (Figure S20) amino acid (Figure S21), fish (Figure S22), isoflavone (Figure S23), vegetables (Figure S24), sweeteners (Figure S25).

. Variable load again was experienced in this class where lysine (LYS) had the largest daily load (weekly average) at 1713.0 ± 1040.0 g/day whereas (5-DHPV) had the minimum (weekly average) at 0.74 ± 0.14 g/day. The food markers selected Here cover a broad range, from artificial sweeteners to vitamins. When compared to previous studies, the magnitude order of these chemicals was different; in this study: 3-methylhistidine > TMAO > stachydrine > 1-methyl-4-pyridone-5-carboxamide > acesulfame > 4-pyridoxic acid > enterolactone > riboflavin > enterodiol. Whereas in a previous study in Australia, the following trend was observed: acesulfame K > stachydrine > 1-methyl-4-pyridone-5-carboxamide > 3-methyl histidine > enterolactone > riboflavin > TMAO > 4-pyridoxic acid > riboflavin > enterodiol [23]. The variable magnitudes between countries highlight a potential perspective for future work, comparing the consumption of certain food groups between nations. It is also expected that food consumption will vary across the year; therefore, WBE is well poised to identify any potential trends in consumption due to seasonality.

### Personal care products and BCIs of exposure

The inclusion of personal care products, such as benzophenones, parabens, and chloroxylenol (CLX) showcases this method’s ability to simultaneously monitor human response to hazardous chemicals [38, 39]. Alongside having a negative impact on human health, parabens and benzophenones have been associated with changes in human lifestyle, such as sunshine or changes in temperature [42]. CLX, a disinfectant, could be associated with other potential external stressors, such as seasonal changes, where increased illness, in winter for example, could require an increased use of disinfectants.

**Fig. 6:**
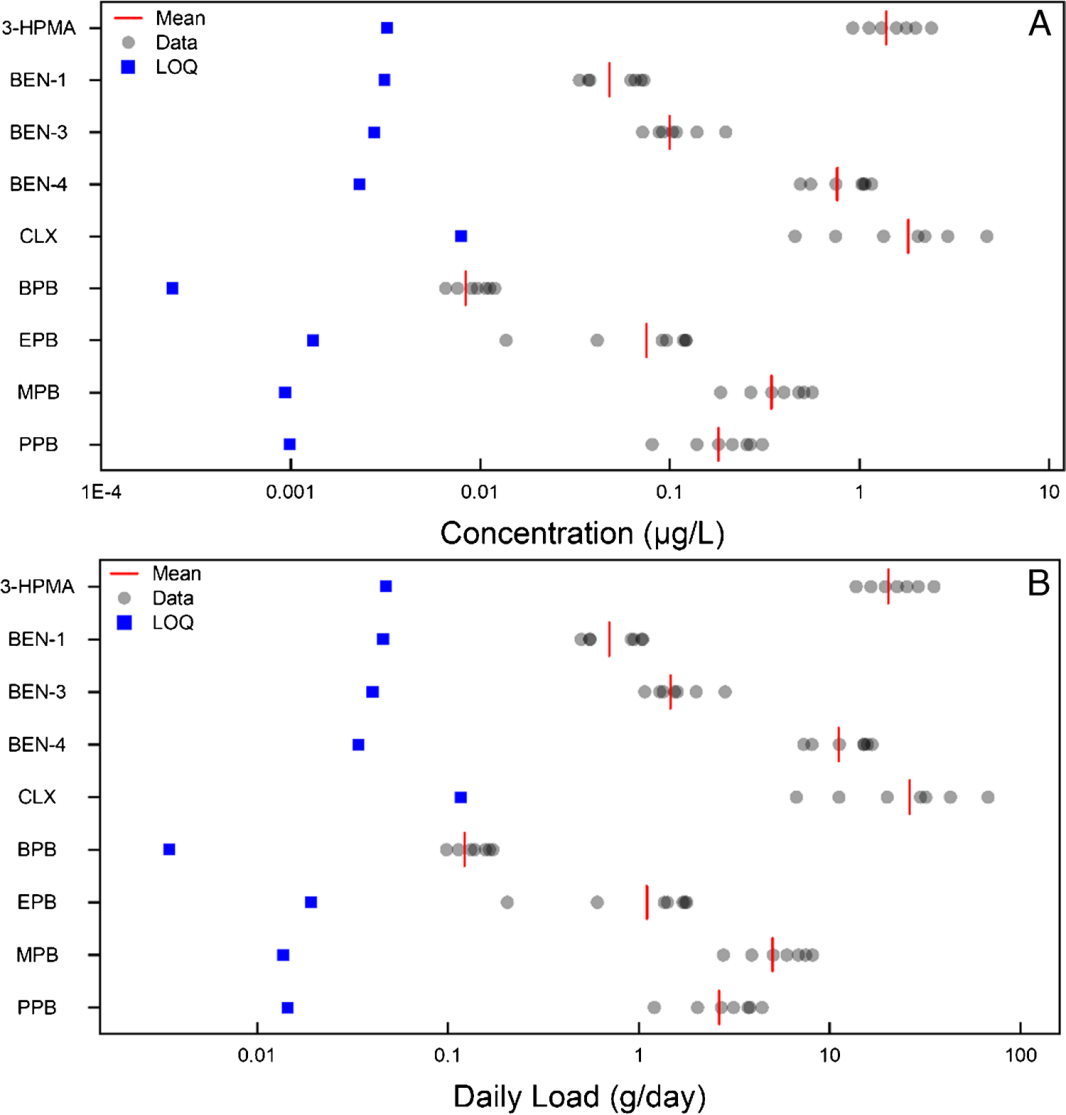
7-day temporal study on the PCP BCIs, quantified in influent wastewater. Figure A details the wastewater concentration, LOQ is determined via the MQL (Table 1). Figure B is the daily load for the same samples. Here, daily load LOQ was calculated by multiplying the MQL by the average daily flow rate (L/day) across the 7-day sampling week. Abbreviations are defined within Table S1

Linking exposure to food and lifestyle habits provides a key understanding of exposure routes, such as acrolein via food or smoking. [93] Increased production of acrolein occurs with frying food, and frequent consumption has been strongly associated with both anxiety and depression [93, 94].Of this sub-category, 9 out of 10 BCIs were detected, all with 100% frequency; it was only benzophenone-2 which was not detected. This is similar to previous trends within the region (average daily loads of PCPs were benzophenone-4 > methylparaben > propylparaben > ethylparaben > benzophenone-1 [42]. The only difference between this work and previous studies is that butylparaben was detected, unlike benzophenone-2, whereas previously this was the inverse; it is expected that following longitudinal monitoring these differences would become negligible [42]. The inclusion of benzophenone-3 and chloroxylenol will improve an understanding of personal care product use and exposure at the community level. Varying loads were detected, from butylparaben (0.14 ± 0.03 g/day, weekly daily average) to CLX (30.07 ± 20.76 g/day, weekly daily average). These results are displayed below in Fig. 6 and Table S8.

Overall, throughout the comprehensive analysis of these six subcategories, key information and insights can be gained about human lifestyle habits. This covers, licit and illicit drug use, food and pharmaceutical consumption, physiological response and personal care products. All areas contribute significantly to an individual’s health and therefore, its analysis within wastewater enables a greater understanding at the metropolitan level.

## Conclusions

A new, comprehensive WBE biomarker method enabling assessment of exposure and health outcomes associations was developed. This multiresidue LC–MS/MS method for the analysis of 204 analytes in wastewater was successfully validated to enable a greater understanding of community health at the metropolitan level via the analysis of community wastewater. Key focus on new endogenous markers allows further study of physiological response to external stressors, alongside the analysis of pharmaceuticals which can be used as a proxy for disease and health status. Future work will focus on the stability of these new analytes and their applicability to temporal (multi-year longitudinal) and spatial analysis across sites, to assess their robustness and subsequent utility in WBE.

Of the 204 BCIs studied, 141 passed a validation with accurate concentration determination in influent wastewater and were deemed to be fully quantitative following method validation. An additional 40 compounds with lower recovery were still quantified (due to low recovery variability) with consistent recovery assessments performed during sample analysis to ensure accuracy. The remaining BCIs are classed as semi-quantitative, as detailed in Table 1. In summary:

1. In the illicit drugs, 11/14 were fully quantitative where 9/14 were quantified in all samples.
2. In the Lifestyle chemicals, 4/5 were fully quantitative, where 100% were quantified in all samples.
3. In the pharmaceutical sub-class, 94/105 were fully quantitative, with 9 having recovery 10 < x % < 50, x % > 120. Within the analysis, 56 pharmaceuticals (including their metabolites) were detected.
4. In the Health sub-class 18/35 were fully quantitative, with 11 having recovery, 10 < x % < 50, x % > 120. In total, 30 were quantified within these samples.
5. In the food sub-class 18/35 were fully quantitative, with 13 having recovery, 10 < x % < 50. In total, 30 were quantified within these samples.
6. In the PCP sub-class, 10/10 were fully quantitative, where 9/10 were quantified in these samples.

This comprehensive multi-biomarker assessment allows for a deeper understanding of community exposure and health outcomes via understanding lifestyle habits through consumption of food, lifestyle, and illicit drugs.

## Supplementary Information

Below is the link to the electronic supplementary material.

Supplementary Material 1 (DOCX 2.26 MB)

## Data Availability

Data will be made available upon request.

## References

[1] United Nations, Department of Economic and Social Affairs, Population Division. World Urbanization Prospects: The 2018 Revision (ST/ESA/SER.A/420). New York: United Nations; 2019. https://www.un-ilibrary.org/content/books/9789210043144. Accessed May 2025.

[2] Giles-Corti BP, Vernez-Moudon AP, Reis RP, Turrell GP, Dannenberg ALP, Badland HP, et al. City planning and population health: a global challenge. Lancet. 2016;388(10062):2912–24. 27671668 10.1016/S0140-6736(16)30066-6

[3] Bian Z, Wang L, Fan R, Sun J, Yu L, Xu M, et al. Genetic predisposition, modifiable lifestyles, and their joint effects on human lifespan: evidence from multiple cohort studies. BMJ evidence-based medicine. 2024;29(4):255–63. 38684374 10.1136/bmjebm-2023-112583

[4] Fadnes LT, Celis-Morales C, Økland J-M, Parra-Soto S, Livingstone KM, Ho FK, et al. Life expectancy can increase by up to 10 years following sustained shifts towards healthier diets in the United Kingdom. Nature food. 2023;4(11):961–5. 37985698 10.1038/s43016-023-00868-w PMC10661734

[5] Oken BS, Chamine I, Wakeland W. A systems approach to stress, stressors and resilience in humans. Behav Brain Res. 2015;282:144–54. 25549855 10.1016/j.bbr.2014.12.047 PMC4323923

[6] Lai FY, O’Brien J, Bruno R, Hall W, Prichard J, Kirkbride P, et al. Spatial variations in the consumption of illicit stimulant drugs across Australia: a nationwide application of wastewater-based epidemiology. Sci Total Environ. 2016;568:810–8. 27267725 10.1016/j.scitotenv.2016.05.207

[7] Burgard DA, Williams J, Westerman D, Rushing R, Carpenter R, LaRock A, et al. Using wastewater-based analysis to monitor the effects of legalized retail sales on cannabis consumption in Washington State, USA. Addiction. 2019;114(9):1582–90. 31211480 10.1111/add.14641 PMC6814135

[8] Jaunay EL, Bade R, Paxton KR, Nadarajan D, Barry DC, Zhai Y, et al. Monitoring the use of novel psychoactive substances in Australia by wastewater-based epidemiology. Sci Total Environ. 2024;919:170473. 38286292 10.1016/j.scitotenv.2024.170473

[9] Salgueiro-Gonzalez N, Béen F, Bijlsma L, Boogaerts T, Covaci A, Baz-Lomba JA, et al. Influent wastewater analysis to investigate emerging trends of new psychoactive substances use in Europe. Water Res. 2024;254:121390. 38430760 10.1016/j.watres.2024.121390

[10] Bilano VM, Gilmour SP, Moffiet TP, d’Espaignet ETP, Stevens GAD, Commar AMA, et al. Global trends and projections for tobacco use, 1990–2025: an analysis of smoking indicators from the WHO Comprehensive Information Systems for Tobacco Control. The Lancet (British edition). 2015;385(9972):966–76. 10.1016/S0140-6736(15)60264-1 25784347

[11] Ceolotto N, Jagadeesan K, Xu L, Standerwick R, Robertson M, Barden R, et al. Assessment of restriction measures implemented during COVID pandemics on community lifestyle choices via wastewater-based epidemiology. J Hazard Mater. 2024;471:134264. 38640675 10.1016/j.jhazmat.2024.134264

[12] Rousis N, Bade R, Gracia-Lor E. Wastewater-based epidemiology as a surveillance tool to assess human consumption of psychotropic substances: Alcohol, nicotine and caffeine as case studies. TrAC Trends Anal Chem. 2023;167:117230.

[13] Ceolotto N, Jagadeesan K, Xu L, Standerwick R, Robertson M, Barden R, et al. Understanding treatment of pain during SARS-CoV-2 pandemic in a two-year intercity longitudinal study using wastewater-based epidemiology. J Hazard Mater. 2024;471:134121. 38636235 10.1016/j.jhazmat.2024.134121

[14] Boogaerts T, Degreef M, Covaci A, van Nuijs ALN. Development and validation of an analytical procedure to detect spatio-temporal differences in antidepressant use through a wastewater-based approach. Talanta. 2019;200:340–9. 31036194 10.1016/j.talanta.2019.03.052

[15] Shao X-T, Zhao Y-T, Jiang B, Li Y-Y, Lin J-G, Wang D-G. Evaluation of three chronic diseases by selected biomarkers in wastewater. ACS ES&T Water. 2023;3(4):943–53.

[16] Hou C, Zhong Y, Zhang L, Liu M, Yan F, Chen M, et al. Estimating the prevalence of hypertension in 164 cities in China by wastewater-based epidemiology. J Hazard Mater. 2023;443:130147. 36283217 10.1016/j.jhazmat.2022.130147

[17] Xiao Y, Shao X-T, Tan D-Q, Yan J-H, Pei W, Wang Z, et al. Assessing the trend of diabetes mellitus by analyzing metformin as a biomarker in wastewater. Sci Total Environ. 2019;688:281–7. 31229825 10.1016/j.scitotenv.2019.06.117

[18] Ceolotto N, Dollamore P, Hold A, Balne B, Jagadeesan KK, Standerwick R, et al. A new wastewater-based epidemiology workflow to estimate community wide non-communicable disease prevalence using pharmaceutical proxy data. J Hazard Mater. 2024;461:132645. 37793253 10.1016/j.jhazmat.2023.132645

[19] Laimou-Geraniou M, Heath D, Heath E. Analytical methods for the determination of antidepressants, antipsychotics, benzodiazepines and their metabolites through wastewater-based epidemiology. Trends Environ Anal Chem. 2023;37:e00192.

[20] Yenet A, Nibret G, Tegegne BA. Challenges to the Availability and Affordability of Essential Medicines in African Countries: A Scoping Review. ClinicoEconomics and outcomes research. 2023;15:443–58. 37332489 10.2147/CEOR.S413546 PMC10276598

[21] Jagadeesan KK, Grant J, Griffin S, Barden R, Kasprzyk-Hordern B. PrAna: an R package to calculate and visualize England NHS primary care prescribing data. BMC Med Inform Decis Mak. 2022;22(1):5. 34991567 10.1186/s12911-021-01727-z PMC8734375

[22] Jagadeesan KK, Elliss H, Standerwick R, Robertson M, Barden R, Kasprzyk-Hordern B. Wastewater-based proteomics: a proof-of-concept for advancing early warning system for infectious diseases and immune response monitoring. J Hazard Mater Lett. 2024;5:100108.

[23] Choi PM, Bowes DA, O’Brien JW, Li J, Halden RU, Jiang G, et al. Do food and stress biomarkers work for wastewater-based epidemiology? A critical evaluation. Sci Total Environ. 2020;736:139654. 32497888 10.1016/j.scitotenv.2020.139654

[24] Shao X-T, Wang Y-S, Gong Z-F, Li Y-Y, Lin J-G, Wang D-G. A feasibility study on cortisol and cortisone as biomarkers for psychological stress in wastewater-based epidemiology. Water Res. 2025;273:123022. 39742636 10.1016/j.watres.2024.123022

[25] Bowers I, Subedi B. Isoprostanes in wastewater as biomarkers of oxidative stress during COVID-19 pandemic. Chemosphere. 2021;271:129489. 33434819 10.1016/j.chemosphere.2020.129489 PMC7778527

[26] Gao Z, Sun H, Xie Y, Ren Y. Assessment of the excretion of oxidative stress biomarkers and anabolic steroids based on sewage: A case study of college students and the general population. Sci Total Environ. 2023;878:163079. 36990235 10.1016/j.scitotenv.2023.163079

[27] Driver EM, Gushgari AJ, Steele JC, Bowes DA, Halden RU. Assessing population-level stress through glucocorticoid hormone monitoring in wastewater. Sci Total Environ. 2022;838(Pt 2):155961. 35588803 10.1016/j.scitotenv.2022.155961

[28] Li J, Choi PM, Gao J, Ren J, O’Brien JW, Thomas KV, et al. In-sewer stability of 31 human health biomarkers and suitability for wastewater-based epidemiology. Water Res. 2024;249:120978. 38071905 10.1016/j.watres.2023.120978

[29] Gracia-Lor E, Castiglioni S, Bade R, Been F, Castrignanò E, Covaci A, et al. Measuring biomarkers in wastewater as a new source of epidemiological information: current state and future perspectives. Environ Int. 2017;99:131–50. 28038971 10.1016/j.envint.2016.12.016

[30] Schug TT, Blawas AM, Gray K, Heindel JJ, Lawler CP. Elucidating the links between endocrine disruptors and neurodevelopment. Endocrinology. 2015;156(6):1941–51. 25714811 10.1210/en.2014-1734 PMC5393340

[31] Haalck I, Székely A, Ramne S, Sonestedt E, von Brömssen C, Eriksson E, et al. Are we using more sugar substitutes? Wastewater analysis reveals differences and rising trends in artificial sweetener usage in Swedish urban catchments. Environ Int. 2024;190:108814. 38917625 10.1016/j.envint.2024.108814

[32] Bowes DA, Driver EM, Savic S, Cheng Q, Whisner CM, Krajmalnik-Brown R, et al. Integrated multiomic wastewater-based epidemiology can elucidate population-level dietary behaviour and inform public health nutrition assessments. Nature food. 2023;4(3):257–66. 37118274 10.1038/s43016-023-00717-w

[33] Choi PM, Tscharke B, Samanipour S, Hall WD, Gartner CE, Mueller JF, et al. Social, demographic, and economic correlates of food and chemical consumption measured by wastewater-based epidemiology. Proc Natl Acad Sci - PNAS. 2019;116(43):21864–73. 31591193 10.1073/pnas.1910242116 PMC6815118

[34] Li J, O’Brien JW, Tscharke BJ, He C, Shimko KM, Shao X, et al. National survey of the occurrence of antimicrobial agents in Australian wastewater and their socioeconomic correlates. Nat Water. 2024;2(12):1166–77.

[35] Rousis NI, Li Z, Bade R, McLachlan MS, Mueller JF, O’Brien JW, et al. Socioeconomic status and public health in Australia: A wastewater-based study. Environ Int. 2022;167:107436. 35914338 10.1016/j.envint.2022.107436

[36] O’Brien JW, Grant S, Banks APW, Bruno R, Carter S, Choi PM, et al. A national wastewater monitoring program for a better understanding of public health: a case study using the Australian Census. Environ Int. 2019;122:400–11. 30554870 10.1016/j.envint.2018.12.003

[37] Wei F, Mortimer M, Cheng H, Sang N, Guo L-H. Parabens as chemicals of emerging concern in the environment and humans: A review. Sci Total Environ. 2021;778:146150. 34030374 10.1016/j.scitotenv.2021.146150

[38] Bledzka D, Gromadzinska J, Wasowicz W. Parabens. From environmental studies to human health. Environ Int. 2014;67:27–42. 24657492 10.1016/j.envint.2014.02.007

[39] Mustieles V, Balogh RK, Axelstad M, Montazeri P, Márquez S, Vrijheid M, et al. Benzophenone-3: Comprehensive review of the toxicological and human evidence with meta-analysis of human biomonitoring studies. Environ Int. 2023;173:107739. 36805158 10.1016/j.envint.2023.107739

[40] O’Malley E, O’Brien JW, Tscharke B, Thomas KV, Mueller JF. Per capita loads of organic UV filters in Australian wastewater influent. Sci Total Environ. 2019;662:134–40. 30690348 10.1016/j.scitotenv.2019.01.140

[41] Karthikraj R, Kannan K. Mass loading and removal of benzotriazoles, benzothiazoles, benzophenones, and bisphenols in Indian sewage treatment plants. Chemosphere. 2017;181:216–23. 28441612 10.1016/j.chemosphere.2017.04.075

[42] Ceolotto N, Jagadeesan K, Xu L, Standerwick R, Robertson M, Barden R, et al. Personal care products use during SARS-CoV-2 pandemic: Environmental and public health impact assessment using wastewater-based epidemiology. Water Res (Oxford). 2025;268(Pt A):122624. 10.1016/j.watres.2024.122624 39490091

[43] Petrie B, Youdan J, Barden R, Kasprzyk-Hordern B. Multi-residue analysis of 90 emerging contaminants in liquid and solid environmental matrices by ultra-high-performance liquid chromatography tandem mass spectrometry. J Chromatogr A. 2016;1431:64–78. 26792447 10.1016/j.chroma.2015.12.036

[44] He K, Echigo S, Asada Y, Itoh S. Determination of caffeine and its metabolites in wastewater treatment plants using solid-phase extraction and liquid chromatography-tandem mass spectrometry. Anal Sci. 2018;34(3):349–54. 29526904 10.2116/analsci.34.349

[45] Boogaerts T, Van Wichelen N, Quireyns M, Burgard D, Bijlsma L, Delputte P, et al. Current state and future perspectives on de facto population markers for normalization in wastewater-based epidemiology: A systematic literature review. Sci Total Environ. 2024;935:173223. 38761943 10.1016/j.scitotenv.2024.173223 PMC11270913

[46] Holton E, Kasprzyk-Hordern B. Multiresidue antibiotic-metabolite quantification method using ultra-performance liquid chromatography coupled with tandem mass spectrometry for environmental and public exposure estimation. Anal Bioanal Chem. 2021;413(23):5901–20. 34498102 10.1007/s00216-021-03573-4 PMC8425450

[47] Kasprzyk-Hordern B, Proctor K, Jagadeesan K, Watkins S, Standerwick R, Barden R, et al. Diagnosing down-the-drain disposal of unused pharmaceuticals at a river catchment level: unrecognized sources of environmental contamination that require nontechnological solutions. Environ Sci Technol. 2021;55(17):11657–66. 34423978 10.1021/acs.est.1c01274 PMC8735766

[48] Sims N, Farkas K, Wade MJ, Kasprzyk-Hordern B, Sims N, Farkas K, et al. Knowledge discovery by analysis of community wastewater reveals factors driving public health risks. Water Res: J Int Assoc Water Pollut Res Contr. 2025;287(Pt A):124370. 10.1016/j.watres.2025.124370 40819445

[49] Choi PM, O’Brien JW, Li J, Jiang G, Thomas KV, Mueller JF. Population histamine burden assessed using wastewater-based epidemiology: The association of 1,4-methylimidazole acetic acid and fexofenadine. Environ Int. 2018;120:172–80. 30096611 10.1016/j.envint.2018.08.009

[50] Chang H, Wan Y, Wu S, Fan Z, Hu J. Occurrence of androgens and progestogens in wastewater treatment plants and receiving river waters: Comparison to estrogens. Water Res (Oxford). 2011;45(2):732–40. 10.1016/j.watres.2010.08.046 20850861

[51] Almazrouei B, Islayem D, Alskafi F, Catacutan MK, Amna R, Nasrat S, et al. Steroid hormones in wastewater: sources, treatments, environmental risks, and regulations. Emerg Contam. 2023;9(2):100210.

[52] Pandopulos AJ, Gerber C, Tscharke BJ, O’Brien J, White JM, Bade R. A sensitive analytical method for the measurement of neurotransmitter metabolites as potential population biomarkers in wastewater. J Chromatogr A. 2020;1612:460623. 31668998 10.1016/j.chroma.2019.460623

[53] Lu Z, Wang Q, Wang M, Fu S, Zhang Q, Zhang Z, et al. Using UHPLC q-trap/MS as a complementary technique to in-depth mine UPLC q-TOF/MS data for identifying modified nucleosides in urine. J Chromatogr B. 2017;1051:108–17. 10.1016/j.jchromb.2017.03.002 28340480

[54] Zhang Y-F, Qi C-B, Yuan B-F, Feng Y-Q. Determination of cytidine modifications in human urine by liquid chromatography - mass spectrometry analysis. Anal Chim Acta. 2019;1081:103–11. 31446947 10.1016/j.aca.2019.07.002

[55] Lim KS, Jenner A, Halliwell B. Quantitative gas chromatography mass spectrometric analysis of 2′-deoxyinosine in tissue DNA. Nat Protoc. 2006;1(4):1995–2002. 17487188 10.1038/nprot.2006.301

[56] Wink DA, Kasprzak KS, Maragos CM, Elespuru RK, Misra M, Dunams TM, et al. DNA Deaminating Ability and Genotoxicity of Nitric Oxide and Its Progenitors. Sci (American Association for the Advancement of Science). 1991;254(5034):1001–3. 10.1126/science.1948068 1948068

[57] Li X, Liu Z, Li Z, Xiong X, Zhang X, Yang C, et al. A simple, rapid and sensitive HILIC LC-MS/MS method for simultaneous determination of 16 purine metabolites in plasma and urine. Talanta. 2024;267:125171. 37696233 10.1016/j.talanta.2023.125171

[58] Patejko M, Struck-Lewicka W, Siluk D, Waszczuk-Jankowska M, Markuszewski MJ. Urinary Nucleosides and Deoxynucleosides. Adv Clin Chem. 2018;83:1–51. 29304899 10.1016/bs.acc.2017.10.001

[59] Garca MF, Demir H, Turan M, Bozan N, Kozan A, Belli ŞB, et al. Assessment of adenosine deaminase (ADA) activity and oxidative stress in patients with chronic tonsillitis. Eur Arch Otorhinolaryngol. 2014;271(6):1797–802. 24305782 10.1007/s00405-013-2843-z

[60] González-Mariño I, Rodil R, Barrio I, Cela R, Quintana JB. Wastewater-based epidemiology as a new tool for estimating population exposure to phthalate plasticizers. Environ Sci Technol. 2017;51(7):3902–10. 28240866 10.1021/acs.est.6b05612

[61] Kappock TJ, Caradonna JP. Pterin-dependent amino acid hydroxylases. Chem Rev. 1996;96(7):2659–756. 11848840 10.1021/cr9402034

[62] Daughton CG. Monitoring wastewater for assessing community health: Sewage Chemical-Information Mining (SCIM). Sci Total Environ. 2018;619–620:748–64. 29161600 10.1016/j.scitotenv.2017.11.102 PMC6091531

[63] Gamagedara S, Gibbons S, Ma Y. Investigation of urinary pteridine levels as potential biomarkers for noninvasive diagnosis of cancer. Clin Chim Acta. 2011;412(1):120–8. 20869359 10.1016/j.cca.2010.09.015

[64] Vasquez-Vivar J, Shi Z, Tan S. Tetrahydrobiopterin in cell function and death mechanisms. Antioxid Redox Signal. 2022;37(1–3):171–83. 34806400 10.1089/ars.2021.0136 PMC9293684

[65] Hilton JF, Christensen KE, Watkins D, Raby BA, Renaud Y, de la Luna S, et al. The molecular basis of glutamate formiminotransferase deficiency. Hum Mutat. 2003;22(5):416. 10.1002/humu.10236 12815595

[66] Singh VP, Bali A, Singh N, Jaggi AS. Advanced glycation end products and diabetic complications. Korean J Physiol Pharmacol. 2014;18(1):1–14. 24634591 10.4196/kjpp.2014.18.1.1 PMC3951818

[67] Ottum MS, Mistry AM. Advanced glycation end-products: modifiable environmental factors profoundly mediate insulin resistance. J Clin Biochem Nutr. 2015;57(1):1–12. 26236094 10.3164/jcbn.15-3 PMC4512899

[68] Khan MI, Ashfaq F, Alsayegh AA, Hamouda A, Khatoon F, Altamimi TN, et al. Advanced glycation end product signaling and metabolic complications: dietary approach. World J Diabetes. 2023;14(7):995–1012. 37547584 10.4239/wjd.v14.i7.995 PMC10401445

[69] Martinez-Moral M-P, Kannan K. Analysis of 19 urinary biomarkers of oxidative stress, nitrative stress, metabolic disorders, and inflammation using liquid chromatography–tandem mass spectrometry. Anal Bioanal Chem. 2022;414(6):2103–16. 35013809 10.1007/s00216-021-03844-0 PMC8747998

[70] Uribarri JMD, Woodruff SRD, Goodman SRD, Cai WMD, Chen XMD, Pyzik RMAMS, et al. Advanced Glycation End Products in Foods and a Practical Guide to Their Reduction in the Diet. J Am Diet Assoc. 2010;110(6):911-6.e12. 20497781 10.1016/j.jada.2010.03.018 PMC3704564

[71] Raibman Spector S, Mayan H, Loebstein R, Markovits N, Priel E, Massalha E, et al. Pyroglutamic acidosis as a cause for high anion gap metabolic acidosis: a prospective study. Sci Rep. 2019;9(1):3554. 30837497 10.1038/s41598-019-39257-4 PMC6400893

[72] Purwaha P, Silva LP, Hawke DH, Weinstein JN, Lorenzi PL. An artifact in LC-MS/MS measurement of glutamine and glutamic acid: in-source cyclization to pyroglutamic acid. Anal Chem. 2014;86(12):5633–7. 24892977 10.1021/ac501451v PMC4063328

[73] Brydges CR, Fiehn O, Mayberg HS, Schreiber H, Dehkordi SM, Bhattacharyya S, et al. Indoxyl sulfate, a gut microbiome-derived uremic toxin, is associated with psychic anxiety and its functional magnetic resonance imaging-based neurologic signature. Sci Rep. 2021;11(1):21011–4. 34697401 10.1038/s41598-021-99845-1 PMC8546034

[74] Saha PP, Gogonea V, Sweet W, Mohan ML, Singh KD, Anderson JT, et al. Gut microbe-generated phenylacetylglutamine is an endogenous allosteric modulator of β2-adrenergic receptors. Nat Commun. 2024;15(1):6696–714. 39107277 10.1038/s41467-024-50855-3 PMC11303761

[75] Ticinesi A, Guerra A, Nouvenne A, Meschi T, Maggi S. Disentangling the complexity of nutrition, frailty and gut microbial pathways during aging: a focus on hippuric acid. Nutrients. 2023;15(5):1138. 36904138 10.3390/nu15051138 PMC10005077

[76] Thai PK, O’Brien J, Jiang G, Gernjak W, Yuan Z, Eaglesham G, et al. Degradability of creatinine under sewer conditions affects its potential to be used as biomarker in sewage epidemiology. Water Res (Oxford). 2014;55:272–9. 10.1016/j.watres.2014.02.035 24631876

[77] Lim YJ, Sidor NA, Tonial NC, Che A, Urquhart BL. Uremic toxins in the progression of chronic kidney disease and cardiovascular disease: mechanisms and therapeutic targets. Toxins. 2021;13(2):142. 33668632 10.3390/toxins13020142 PMC7917723

[78] Li D, O’Brien JW, Tscharke BJ, Choi PM, Ahmed F, Thompson J, et al. Trends in artificial sweetener consumption: a 7-year wastewater-based epidemiology study in Queensland, Australia. Sci Total Environ. 2021;754:142438. 33254907 10.1016/j.scitotenv.2020.142438

[79] Li D, Zheng Q, Thomas KV, Dang AK, Binh VN, Anh NTK, et al. Use of artificial sweeteners and caffeine in a population of Hanoi: An assessment by wastewater-based epidemiology. Sci Total Environ. 2023;868:161515. 36634775 10.1016/j.scitotenv.2023.161515

[80] Choi PM, O’Brien JW, Tscharke BJ, Mueller JF, Thomas KV, Samanipour S. Population socioeconomics predicted using wastewater. Environ Sci Technol Lett. 2020;7(8):567–72.

[81] Debras C, Chazelas E, Srour B, Druesne-Pecollo N, Esseddik Y, Szabo de Edelenyi F, et al. Artificial sweeteners and cancer risk: Results from the NutriNet-Santé population-based cohort study. PLoS Med. 2022;19(3):e1003950. 35324894 10.1371/journal.pmed.1003950 PMC8946744

[82] Feliciano RP, Boeres A, Massacessi L, Istas G, Ventura MR, Nunes dos Santos C, et al. Identification and quantification of novel cranberry-derived plasma and urinary (poly)phenols. Arch Biochem Biophys. 2016;599:31–41. 26836705 10.1016/j.abb.2016.01.014

[83] Lee CC, Kim JH, Kim JS, Oh YS, Han SM, Park JHY, et al. 5-(3′,4′-dihydroxyphenyl-γ-valerolactone), a major microbial metabolite of proanthocyanidin, attenuates THP-1 monocyte-endothelial adhesion. Int J Mol Sci. 2017;18(7):1363. 28672844 10.3390/ijms18071363 PMC5535856

[84] Singh A, D’Amico D, Andreux PA, Dunngalvin G, Kern T, Blanco-Bose W, et al. Direct supplementation with Urolithin A overcomes limitations of dietary exposure and gut microbiome variability in healthy adults to achieve consistent levels across the population. Eur J Clin Nutr. 2022;76(2):297–308. 34117375 10.1038/s41430-021-00950-1 PMC8821002

[85] Singh A, D’Amico D, Andreux PA, Fouassier AM, Blanco-Bose W, Evans M, et al. Urolithin A improves muscle strength, exercise performance, and biomarkers of mitochondrial health in a randomized trial in middle-aged adults. Cell Rep Med. 2022;3(5):100633. 35584623 10.1016/j.xcrm.2022.100633 PMC9133463

[86] Olayanju JB, Bozic D, Naidoo U, Sadik OA. A comparative review of key isothiocyanates and their health benefits. Nutrients. 2024;16(6):757. 38542669 10.3390/nu16060757 PMC10974736

[87] Agrawal S, Winnik B, Buckley B, Mi L, Chung F-L, Cook TJ. Simultaneous determination of sulforaphane and its major metabolites from biological matrices with liquid chromatography–tandem mass spectroscopy. J Chromatogr B. 2006;840(2):99–107. 10.1016/j.jchromb.2006.04.046 16766235

[88] Dai Z, Zheng W, Locasale JW. Amino acid variability, tradeoffs and optimality in human diet. Nat Commun. 2022;13(1):6683. 36335142 10.1038/s41467-022-34486-0 PMC9637229

[89] Reeds PJ. Dispensable and indispensable amino acids for humans. J Nutr. 2000;130(7):1835S-S1840. 10867060 10.1093/jn/130.7.1835S

[90] Górska-Warsewicz H, Laskowski W, Kulykovets O, Kudlińska-Chylak A, Czeczotko M, Rejman K. Food products as sources of protein and amino acids-the case of Poland. Nutrients. 2018;10(12):1977. 30551657 10.3390/nu10121977 PMC6315330

[91] Lloyd AJ, Beckmann M, Haldar S, Seal C, Brandt K, Draper J. Data-driven strategy for the discovery of potential urinary biomarkers of habitual dietary exposure. Am J Clin Nutr. 2013;97(2):377–89. 23269817 10.3945/ajcn.112.048033

[92] Cross AJ, Major JM, Sinha R. Urinary biomarkers of meat consumption. Cancer Epidemiol Biomarkers Prev. 2011;20(6):1107–11. 21527577 10.1158/1055-9965.EPI-11-0048 PMC3111815

[93] Jiang K, Huang C, Liu F, Zheng J, Ou J, Zhao D, et al. Origin and fate of acrolein in foods. Foods. 2022;11(13):1976. 35804791 10.3390/foods11131976 PMC9266280

[94] Wang A, Wan X, Zhuang P, Jia W, Ao Y, Liu X, et al. High fried food consumption impacts anxiety and depression due to lipid metabolism disturbance and neuroinflammation. Proc Natl Acad Sci-PNAS. 2023;120(18):e2221097120-e. 10.1073/pnas.2221097120 PMC10160962 37094155

