# Supplementary material for: A new wide-scope, multi-biomarker wastewater-based epidemiology analytical method to monitor the health and well-being of inhabitants at a metropolitan scale
Source: Anal Bioanal Chem. 2025 Sep 13;417(26):5983–6005. doi: 10.1007/s00216-025-06097-3 (PMC12532643; doi:10.1007/s00216-025-06097-3)
Supplement: Supplementary file 1 — Supplementary Material 1 (DOCX 2.26 MB) [file 216_2025_6097_MOESM1_ESM.docx]

**Electronic Supplementary Material**

**A new wide-scope, multi-biomarker wastewater-based epidemiology analytical method to monitor the health and well-being of inhabitants at a metropolitan scale**

Harry Elliss^a, b, c^, Kit Proctor^d^, Megan Robertson^b, e^, John Bagnall^b, e^, Barbara Kasprzyk-Hordern^a, b, c *^

^a^ *Department of Chemistry, University of Bath, Claverton Down, Bath, BA2 7AY, UK*

^b^ *Centre of Excellence in Water-Based Early-Warning Systems for Health Protection, University of Bath, Claverton Down, Bath, BA2 7AY, UK*

^c^ *Institute of Sustainability and Climate Change, University of Bath, Claverton Down, Bath, BA2 7AY, UK*

^d^ *Chemical Characterisation Facility, University of Bath, Claverton Down, Bath, BA2 7AY, UK*

^e^ *Wessex Water Service Ltd., Claverton Down, Bath, BA2 7WW, UK*

^*^Author for correspondence

*E-mail address*: [bkh20@bath.ac.uk](mailto:bkh20@bath.ac.uk) (Barbara Kasprzyk-Hordern)

**Table S1.** BCIs under study within this analytical method

| Broader Class | Sub-class | Parent BCI* | Abbreviation | Metabolite(s) | Additional information |
| --- | --- | --- | --- | --- | --- |
| Illicit drugs | Stimulant | 4-methylmethcathinone | 4-MMC | - | - |
|  |  | Amphetamine | AMP | - | - |
|  |  | Cocaine | COC | 1) anhydroecgonine methylester (AME)  2) benzoylecgonine (BZE)  3) cocaethylene (COE) | 1) Metabolite of cocaine (smoked crack cocaine)  2) Primary metabolite of cocaine  3) Metabolite of cocaine (during co-consumption with alcohol) |
|  |  | Ketamine | KET | Norketamine (nKET) | - |
|  |  | Methamphetamine | MAMP |  | - |
|  |  | 3,4 – methylenedioxyamphetamine | MDA |  | - |
|  |  | 3,4 – methylenedioxymethamphetamine | MDMA |  | - |
|  |  | Methylenedioxypyrovalerone | MDPV |  | - |
|  | Opiods | Heroin | HER | 6-monoacetylmorphine (6-MAM) | - |
| Lifestyle chemicals | Stimulants | Caffeine | CAF | 1) 7-methylxanthine (7-MEX)  2) Paraxanthine (PAX) | 1) Metabolite of theobromine and caffeine  2) Metabolite of caffeine |
|  |  | Nicotine | NIC | Cotinine (COT) | Metabolite of nicotine |
| Pharmaceuticals | Anti-epileptic | Carbamazepine | CBZ | 1) carbamazepine-10,11-epoxide (CBZe)  2) 10,11-dihydro-10-hydroxy carbamazepine (dhhCBZ) | both 1 and 2 are metabolites of carbamazepine |
|  |  | Gabapentin | GAB |  |  |
|  |  | Levetiracetam | LVT |  | - |
|  |  | Pregabalin | PRG | N-methyl pregabalin (mPRG) | Metabolite of pregabalin |
|  |  | Topiramate | TOP |  |  |
|  | Anti-viral | Oseltamivir | OSE |  | - |
|  | Anxiety | Amitriptyline | AMT |  | - |
|  |  | Citalopram | CIT | Desmethyl citalopram (dCIT) | Metabolite of citalopram |
|  |  | Clobazam | CLO |  | - |
|  |  | Diazepam | DZP | Desmethyl diazepam (dDZP) | Metabolite of diazepam |
|  |  | Duloxetine | DLX |  | - |
|  |  | Fluoxetine | FLX | Norfluoxetine (nFLX | Metabolite of fluoxetine |
|  |  | Mirtazapine | MTZ | N-desmethyl mirtazapine (dMTZ) | Anti-depressant |
|  |  | Nitrazepam | NIT |  | - |
|  |  | Nortriptyline | NTP | 10-OH nortriptyline (OH-NTP) | Metabolite of nortriptyline |
|  |  | Oxazepam | OXZ |  | - |
|  |  | Quetiapine | QUT | 7-OH Quetiapine (7-OH-QUT) | Metabolite of quetiapine |
|  |  | Risperidone | RIS |  | - |
|  |  | Sertraline | SRT | Norsertraline (nSRT) | Metabolite of sertraline |
|  |  | Temazepam | TEM |  | - |
|  |  | Venlafaxine | VEN | Desvenlafaxine (dVEN) | Metabolite of venlafaxine |
|  | Asthma/allergy | Fexofenadine | FEX |  | - |
|  |  | Salbutamol | SAL |  | - |
|  | Cancer | Capecitabine | CAP |  | - |
|  |  | Ifosfamide | IFO |  | - |
|  |  | Imatinib | IMT |  | - |
|  |  | Methotrexate | MTX |  | - |
|  |  | Tamoxifen | TAM |  | - |
|  | Cardiovascular | Amlodipine | AML | O-des[2-aminoethyl]O-carboxymethyl dehydroamlodipine (mAML) | Metabolite of amlodipine |
|  |  | Atenolol | ATN |  | - |
|  |  | Atorvastatin | ATO | 2-OH Atorvastatin (2OH-ATO) | Metabolite of atorvastatin |
|  |  | Bendroflumethiazide | BFM |  | - |
|  |  | Bezafibrate | BEZ |  | - |
|  |  | Bisoprolol | BSP |  | - |
|  |  | Diltiazem | DLT | N-desmethyl diltiazem (N-dDLT) | Metabolite of dlitiazem |
|  |  | Gemfibrozil | GEM |  | - |
|  |  | Irbesartan | IRB |  | - |
|  |  | Lisinopril | LIS |  | - |
|  |  | Metoprolol | MEP | O-desmethyl metoprolol (odMEP) | Metabolite of metoprolol |
|  |  | Nicorandil | NCR |  | - |
|  |  | Propranolol | PRP |  | - |
|  |  | Simvastatin | SIM |  | - |
|  |  | Valsartan | VAL | 4-hydroxy valsartan (4OH-VAL) | Metabolite of valsartan |
|  |  | Verapamil | VER | p-O-desmethyl verapamil (O-dVER) | Metabolite of verapamil |
|  | Dementia | Donepezil | DOP |  | - |
|  |  | Memantine | MMT |  | - |
|  | Diabetes | Gliclazide | GLC | 4-OH-Gliclazide (4OH-GLC) | Metabolite of gliclazide |
|  |  | Metformin | MET | Guanyl Urea (GUR) | Metabolite of metformin |
|  |  | Sitagliptin | SIT |  | GUD/ED |
|  | GUD/ED | Sildenafil | SIL | N-desmethyl sildenafil (dSIL) | Metabolite of sildenafil |
|  | Hormone | 17α-Ethinylestradiol | EE2 |  | - |
|  |  | Beclomethasone | BEC |  | - |
|  |  | Betamethasone | BET |  | - |
|  |  | Dexamethasone | DEX |  | - |
|  |  | Budesonide | BUD |  | - |
|  |  | Methylprednisolone | MPD |  | - |
|  |  | Prednisolone | PDN |  | - |
|  | Insomnia | Zolpidem | ZOL |  | - |
|  | Pain | Acetaminophen | APAP |  | - |
|  |  | Buprenorphine | BUP |  | - |
|  |  | Codeine | COD | Norcodeine (nCOD) | Metabolite of codeine |
|  |  | Dihydrocodeine | dCOD |  | - |
|  |  | Diclofenac | DIC | 4-hydroxy diclofenac (4OH-DIC) | Metabolite of diclofenac |
|  |  | Ibuprofen | IBU | 2-OH ibuprofen (2OH-IBU) | Metabolite of ibuprofen |
|  |  | Ketoprofen | KEP | Dihydroketoprofen (dhKEP) | Metabolite of ketoprofen |
|  |  | Methadone | MED | 2-ethylidene-1,5-dimethyl-3,3-diphenylpyrrolidine (EDDP) | Metabolite of methadone |
|  |  | Morphine | MOR | 1) dihydromorphine (dhMOR) 2) Normorphine (nMOR) | both 1 and 2 are metabolites of morphine |
|  |  | Naproxen | NAP | O-desmethyl naproxen (O-dNAP) | Metabolite of naproxen |
|  |  | Tramadol | TRM | 1) O-desmethyl tramadol (O-dTRM) 2) N-desmethyl tramadol (N-dTRM) | both 1 and 2 are metabolites of tramadol |
|  | Stimulant | Ephedrine | EPH |  | Stimulant |
|  |  | Norephedrine | NEPH |  | - |
|  | Ulcer | Cimetidine | CIM |  | - |
|  |  | Lansoprazole | LAN | 1) 5-OH lansoprazole (5OH-LAN) 2) Lansoprazole sulfone (LANs) | both 1 and 2 are metabolites of lansoprazole |
|  |  | Ranitidine | RAN | Ranitidine N-oxide (RANno) | Metabolite of ranitidine |
| Human markers | Advanced glycation end-product | Nε-(1-Carboxyethyl)-L-lysine | CEL |  | - |
|  |  | Nε-(1-Carboxymethyl)-L-lysine | CML |  |  |
|  | Deficiencies | Formiminoglutamic acid | FIGLU |  | Folate deficiency |
|  |  | Pyroglutamic acid | PYRa |  | Glutathione deficiency |
|  | DNA-related | 2’-deoxyguanosine | 2-DGN |  | Metabolic/DNA related disorder |
|  |  | 5-methyl-2'-deoxycytidine | 5m-2-DCT |  | DNA methylation |
|  |  | Deoxyadenosine | 2-DAD | 2'-deoxyinosine (2-DIN) | Metabolic/DNA related disorder |
|  |  | Hydroxymethyl uracil | hmURC |  | Oxidative stress |
|  | General Human | 1,4‑methylimidazoleacetic acid | MIA |  | Histamine burden |
|  |  | 5-hydroxyindole acetic acid | 5-HIAA |  | Metabolite of serotonin |
|  |  | Adenosine | ADN |  | General human |
|  |  | Creatinine | CRE |  | Muscle breakdown |
|  |  | Hippuric acid | HIPa |  | Uremic toxin (gut derived) |
|  |  | Indoxyl sulfate | INDs |  | Uremic toxin (gut derived) |
|  |  | Inosine | INS |  | - |
|  |  | Phenylacetyl L-glutamine | PhAG |  | - |
|  | Hormones | Androstenedione | ASD |  | - |
|  |  | Cortisol | CSL |  | - |
|  |  | Cortisone | CSN |  | - |
|  |  | Dihydrotestosterone | DHT |  | - |
|  |  | Estradiol | E2 |  | - |
|  |  | Estrone | E1 |  | - |
|  |  | Progesterone | PGS |  | - |
|  |  | Testosterone | PYRa |  | - |
|  | Pteridine | Dihydrobiopterin | DHB |  | - |
|  |  | Neopterin | NPT |  | - |
|  |  | Pterin | PTE |  | - |
|  | Stress | 3-nitro-L-tyrosine | 3-NTY |  | Nitrative stress |
|  |  | 3-chloro-L-tyrosine | 3-CTY |  | Oxidative stress |
|  |  | 5-Hydroxymethyl-2'-deoxyuridine | 5-hm-2-DUD |  | Oxidative stress |
|  |  | 8-hydroxyguanosine | 8OH-GNS |  | Oxidative stress |
|  |  | 8-hydroxyguanine | 8-OH-GNN |  | Oxidative stress |
|  |  | Asymmetric dimethyl arginine | ADMA |  | Oxidative stress |
|  |  | 4-Hydroxy-2-nonenal mercapturic acid | HNE-MA |  | Lipid peroxidation |
| Food | Alkaloid | Stachydrine | STC |  | Meat |
|  | Amino acid | 1-methylhistidine | 1-MeH |  | Meat |
|  |  | 3-methylhistidine | 3-MeH |  | Meat |
|  |  | Carnitine | CAR |  | - |
|  |  | Histidine | HTD |  | - |
|  |  | Lysine | LYS |  | - |
|  |  | Methionine | MNN |  | - |
|  |  | Phenylalanine | PAL |  | - |
|  |  | Tryptophan | TRP |  | - |
|  |  | Valine | VLN |  | - |
|  | Artificial sweetener | Acesulfame K | ACSK |  | - |
|  |  | Aspartame | APT |  | - |
|  |  | Saccharin | SAC |  | - |
|  |  | Sucralose | SUC |  |  |
|  | Cruciferous vegetable | D,L-Sulforaphane N-acetyl L-cysteine | SFPac |  | - |
|  | Fish | 3-carboxy-4-methyl-5-propyl-2-furanpropanoic acid | CMPF |  | - |
|  |  | Trimethylamine N-oxide | TMAO |  | - |
|  | Isoflavone | Daidzein | DDZ | Equol (EQU) | Metabolite of isoflavone |
|  |  | Genistein | GEN | Equol (EQU) | - |
|  |  | Glycitein | GYT |  | - |
|  | Lignan | Enterodiol | ETD |  | - |
|  |  | Enterolactone | ETL |  | Metabolite of polyphenol |
|  | Polyphenol | Epicatechin | ECT | 5-(3′,4′-Dihydroxyphenyl)-γ-valerolactone (5-DHPV) | - |
|  |  | Ferulic Acid | FERa |  | - |
|  |  | Phloretin | PLO |  | - |
|  |  | Resveratrol | RVT |  |  |
|  |  | Urolithin A | UROA |  | Polyphenol metabolite |
|  | Vitamin | 1-methyl-2-pyridone-5-carboxamide | 2-PY |  | Vitamin B3 |
|  |  | 4-Pyridoxic acid | 4-PYa |  | Vitamin B6 |
|  |  | a-carboxyethyl hydroxychroman | a-CEHC |  | Vitamin E |
|  |  | N-methylnicotinamide | nmNTA |  | Vitamin B3 |
|  |  | Pantothenic acid | PANa |  | Vitamin B5 |
|  |  | Riboflavin | RIB |  | Vitamin B2 |
| Personal care products | Food toxicant | 3-hydroxypropyl mercapturic acid | 3-HPMA |  | Metabolite of acrolein |
|  | Benzophenone | Benzophenone-1 | BEN-1 |  | Metabolite of Benzophenone-3 |
|  |  | Benzophenone-2 | BEN-2 |  | - |
|  |  | Benzophenone-4 | BEN-4 |  | - |
|  | Industrial chemical | Bisphenol A | BPA |  | - |
|  | Antimicrobial | Chloroxylenol | CLX |  | Antimicrobial |
|  | Paraben | Butylparaben | BPB |  | - |
|  |  | Ethylparaben | EPB |  | - |
|  |  | Methylparaben | MPB |  | - |
|  |  | Propylparaben | PPB |  | - |

*the parent molecule is the primary target, i.e the parent pharmaceutical or the target human marker, in the case where only an endogenous marker is targeted (food & health subclasses) it remains in column 3. All related metabolites are displayed in column 5.

**Table S2.** Name, CAS-No and supplier for all analytes under study within this manuscript

| Class | Analyte | CAS-No | Supplier |
| --- | --- | --- | --- |
| Illicit drugs | 4-methylmethcathinone | 1189805-46-6 | Sigma Aldrich |
|  | Amphetamine | 300-62-9 | LGC (Cerilliant) |
|  | Cocaine | 50-36-2 | LGC (Cerilliant) |
|  | Anhydroecgonine methylester | 43021-26-7 | Sigma Aldrich (Cerilliant) |
|  | Benzoylecgonine | 519-09-5 | Sigma Aldrich |
|  | Cocaethylene | 529-38-4 | Sigma Aldrich (Cerilliant) |
|  | Heroin | 561-27-3 | Sigma Aldrich (Cerilliant) |
|  | 6-monoacetylmorphine | 2784-73-8 | Sigma Aldrich (Cerilliant) |
|  | Ketamine | 6740-88-1 | Sigma Aldrich |
|  | Norketamine | 35211-10-0 | Sigma Aldrich |
|  | Methamphetamine | 537-46-2 | LGC (Cerilliant) |
|  | 3,4 – methylenedioxyamphetamine | 4764-17-4 | Sigma Aldrich |
|  | 3,4 – methylenedioxymethamphetamine | 42542-10-9 | LGC |
|  | Methylenedioxypyrovalerone | 687603-66-3 | Sigma Aldrich |
| Lifestyle chemicals | Caffeine | 58-08-2 | Sigma Aldrich |
|  | 7-methylxanthine | 552-62-5 | Cambridge Bioscience |
|  | Paraxanthine | 611-59-6 | Sigma Aldrich |
|  | Nicotine | 54-11-5 | Sigma Aldrich |
|  | Cotinine | 486-56-6 | Sigma Aldrich (Cerilliant) |
| Pharmaceuticals | 17α-Ethinylestradiol | 57-63-6 | Sigma Aldrich |
|  | Acetaminophen | 103-90-2 | Sigma Aldrich |
|  | Amitriptyline | 50-48-6 | Sigma-Aldrich |
|  | Amlodipine | 88150-42-9 | Sigma-Aldrich |
|  | O-des[2-aminoethyl]O-carboxymethyl dehydroamlodipine | 113994-45-9 | TRC |
|  | Atenolol | 29122-68-7 | Sigma-Aldrich |
|  | Atorvastatin | 134523-00-5 | LGC |
|  | 2-OH Atorvastatin | 214214-86-4 | TRC |
|  | Beclomethasone | 4419-39-0 | LGC |
|  | Betamethasone | 378-44-9 | LGC |
|  | Dexamethasone | 50-02-2 | LGC |
|  | Bendroflumethiazide | 73-48-3 | Sigma-Aldrich |
|  | Bezafibrate | 41859-67-0 | Sigma-Aldrich |
|  | Bisoprolol | 66722-44-9 | Sigma-Aldrich |
|  | Budesonide | 51333-22-3 | Sigma-Aldrich |
|  | Buprenorphine | 52485-79-7 | LGC |
|  | Capecitabine | 154361-50-9 | Sigma-Aldrich |
|  | Carbamazepine | 298-46-4 | Sigma-Aldrich |
|  | Carbamazepine-10,11-epoxide | 36507-30-9 | LGC |
|  | 10,11-dihydro-10-hydroxy carbamazepine | 29331-92-8 | LGC |
|  | Cimetidine | 51481-61-9 | Sigma-Aldrich |
|  | Citalopram | 59729-33-8 | Sigma-Aldrich |
|  | Desmethyl citalopram | 62498-67-3 | TRC |
|  | Clobazam | 22316-47-8 | LGC |
|  | Codeine | 76-57-3 | Sigma-Aldrich |
|  | Norcodeine | 467-15-2 | Sigma-Aldrich (Cerilliant) |
|  | Dihydrocodeine | 125-28-0 | Sigma-Aldrich |
|  | Diazepam | 439-14-5 | Sigma-Aldrich (Cerilliant) |
|  | Desmethyl diazepam | 1088-11-5 | LGC |
|  | Diclofenac | 1537-86-5 | Sigma-Aldrich |
|  | 4-hydroxy diclofenac | 64118-84-9 | Sigma-Aldrich |
|  | Diltiazem | 42399-41-7 | Sigma-Aldrich |
|  | N-desmethyl diltiazem | 130606-60-9 | Sigma-Aldrich |
|  | Donepezil | 120014-06-4 | LGC |
|  | Duloxetine | 116539-59-4 | LGC |
|  | Ephedrine | 299-42-3 | Sigma-Aldrich |
|  | Fexofenadine | 83799-24-0 | LGC |
|  | Fluoxetine | 54910-89-3 | LGC (Cerilliant) |
|  | Norfluoxetine | 83891-03-6 | LGC (Cerilliant) |
|  | Gabapentin | 60142-96-3 | Sigma-Aldrich |
|  | Gemfibrozil | 25812-30-0 | Sigma-Aldrich |
|  | Gliclazide | 21187-98-4 | LGC |
|  | 4-OH-Gliclazide | 87368-00-1 | Sigma-Aldrich |
|  | Ibuprofen | 15687-27-1 | Sigma-Aldrich |
|  | 2-OH ibuprofen | 51146-55-5 | Sigma-Aldrich |
|  | Ifosfamide | 3778-73-2 | Sigma-Aldrich |
|  | Imatinib | 152459-95-5 | Sigma-Aldrich |
|  | Irbesartan | 138402-116 | LGC |
|  | Ketoprofen | 22071-15-4 | Sigma-Aldrich |
|  | Dihydroketoprofen | 59960-32-6 | Sigma-Aldrich |
|  | Lansoprazole | 103577-45-3 | LGC |
|  | 5-OH Lansoprazole | 131926-96-0 | Sigma-Aldrich |
|  | Lansoprazole Sulfone | 131926-99-3 | LGC |
|  | Levetiracetam | 102767-28-2 | Sigma-Aldrich |
|  | Lisinopril | 76547-98-3 | LGC |
|  | Methadone | 76-99-3 | Sigma-Aldrich (Cerilliant) |
|  | 2-ethylidene-1,5-dimethyl-3,3-diphenylpyrrolidine | 30223-73-5 | LGC (Cerilliant) |
|  | Memantine | 19982-08-2 | Sigma-Aldrich |
|  | Metformin | 657-24-9 | Sigma-Aldrich |
|  | Guanyl Urea | 141-83-3 | Sigma-Aldrich |
|  | Methotrexate | 59-05-2 | LGC |
|  | Methylprednisolone | 83-43-2 | Sigma-Aldrich |
|  | Metoprolol | 51384-51-1 | Sigma-Aldrich |
|  | O-desmethyl metoprolol | 62572-94-5 | Sigma-Aldrich |
|  | Mirtazapine | 85650-52-8 | Sigma-Aldrich |
|  | Morphine | 57-27-2 | Sigma-Aldrich (Cerilliant) |
|  | Dihydromorphine | 509-60-4 | Sigma-Aldrich (Cerilliant) |
|  | Normorphine | 466-97-7 | Sigma-Aldrich (Cerilliant) |
|  | Naproxen | 22204-53-1 | TRC |
|  | O-desmethyl naproxen | 52079-10-4 | Sigma-Aldrich (Cerilliant) |
|  | Nicorandil | 65141-46-0 | Sigma-Aldrich |
|  | Nitrazepam | 146-22-5 | Sigma-Aldrich |
|  | Norephedrine | 492-39-7 | Sigma-Aldrich |
|  | Nortriptyline | 72-69-5 | Sigma-Aldrich |
|  | 10-OH nortriptyline | 47132-16-1 | LGC |
|  | Oseltamivir | 196618-13-0 | TRC |
|  | Oxazepam | 604-75-1 | Sigma-Aldrich (Cerilliant) |
|  | Prednisolone | 50-24-8 | LGC |
|  | Pregabalin | 148553-50-8 | Sigma-Aldrich |
|  | N-methyl pregabalin | 1155843-61-0 | Sigma-Aldrich (Cerilliant) |
|  | Propranolol | 525-66-6 | Sigma-Aldrich |
|  | Quetiapine | 111974-69-7 | LGC |
|  | 7-OH Quetiapine | 139079-39-3 | LGC |
|  | Ranitidine | 66357-35-5 | Sigma-Aldrich |
|  | Ranitidine N-oxide | 73857-20-2 | Sigma-Aldrich |
|  | Risperidone | 106266-06-2 | LGC |
|  | Salbutamol | 18559-94-9 | LGC |
|  | Sertraline | 79617-96-2 | LGC |
|  | Norsertraline | 87857-41-8 | LGC |
|  | Sildenafil | 139755-83-2 | Sigma-Aldrich (Cerilliant) |
|  | N-desmethyl sildenafil | 139755-82-1 | Sigma-Aldrich |
|  | Simvastatin | 79902-63-9 | Sigma-Aldrich |
|  | Sitagliptin | 486460-32-6 | TRC |
|  | Tamoxifen | 10540-29-1 | LGC |
|  | Temazepam | 846-50-4 | Sigma-Aldrich (Cerilliant) |
|  | Topiramate | 97240-79-4 | LGC |
|  | Tramadol | 75377-45-6 | Sigma-Aldrich |
|  | O-desmethyl tramadol | 144830-15-9 | LGC |
|  | N-desmethyl tramadol | 144830-14-8 | LGC |
|  | Valsartan | 137862-53-4 | Sigma-Aldrich |
|  | 4-hydroxy valsartan | 188259-69-0 | Cayman Chemical |
|  | Venlafaxine | 93413-69-5 | Sigma-Aldrich |
|  | Desvenlafaxine | 93413-62-8 | Sigma-Aldrich |
|  | Verapamil | 52-53-9 | LGC |
|  | p-O-desmethyl verapamil | 77326-93-3 | TRC |
|  | Zolpidem | 82626-48-0 | Sigma-Aldrich |
| Human markers | 1,4‑methylimidazoleacetic acid | 2625-49-2 | Cambridge Bioscience |
|  | 2’-deoxyguanosine^*^ | 961-07-9 | Sigma-Aldrich |
|  | 2'-deoxyinosine | 890-38-0 | Sigma-Aldrich |
|  | 3-nitro-L-tyrosine | 621-44-3 | Sigma-Aldrich |
|  | 3-chloro-L-tyrosine | 7423-93-0 | Sigma-Aldrich |
|  | 5-Hydroxymethyl-2'-deoxyuridine | 5116-24-5 | Cambridge Bioscience |
|  | 5-hydroxyindole acetic acid | 54-16-0 | Sigma-Aldrich |
|  | 5-methyl-2'-deoxycytidine | 838-07-3 | Cambridge Bioscience |
|  | 8-hydroxyguanosine | 3868-31-3 | TRC |
|  | 8-hydroxyguanine | 5614-64-2 | Cambridge Bioscience |
|  | Adenosine | 58-61-7 | Cambridge Bioscience |
|  | Androstenedione | 63-05-8 | Sigma-Aldrich |
|  | Asymmetric dimethyl arginine | 30315-93-6 | Sigma-Aldrich |
|  | Cortisol | 50-23-7 | Sigma-Aldrich |
|  | Cortisone | 53-06-5 | Sigma-Aldrich |
|  | Creatinine | 60-27-5 | Sigma-Aldrich |
|  | Deoxyadenosine | 958-09-8 | Sigma-Aldrich |
|  | Dihydrobiopterin | 6779-87-9 | Cambridge Bioscience |
|  | Dihydrotestosterone | 521-18-6 | Sigma-Aldrich |
|  | Estradiol | 50-28-2 | Sigma-Aldrich |
|  | Estrone | 53-16-7 | Sigma-Aldrich |
|  | Formiminoglutamic acid | 816-90-0 | LGC |
|  | Hippuric acid | 495-69-2 | Sigma-Aldrich |
|  | 4-Hydroxy-2-nonenal mercapturic acid | 146764-24-1 | Cayman Chemical |
|  | Hydroxymethyl uracil | 4433-40-3 | LGC |
|  | Indoxyl sulfate | 487-94-5 | Cambridge Bioscience |
|  | Inosine | 58-63-9 | Sigma-Aldrich |
|  | Nε-(1-Carboxyethyl)-L-lysine | 5746-03-2 | Cambridge Bioscience |
|  | Nε-(1-Carboxymethyl)-L-lysine | 5746-04-3 | Cambridge Bioscience |
|  | Neopterin | 2009-64-5 | Sigma-Aldrich |
|  | Phenylacetyl L-glutamine | 28047-15-6 | Cambridge Bioscience |
|  | Pterin | 2236-60-4 | Cambridge Bioscience |
|  | Progesterone | 57-83-0 | Sigma-Aldrich |
|  | Pyroglutamic acid | 98-79-3 | LGC |
|  | Testosterone | 58-22-0 | Sigma-Aldrich |
| Food | 1-methyl-2-pyridone-5-carboxamide | 701-44-0 | Cambridge Bioscience |
|  | 1-methylhistidine | 332-80-9 | Cambridge Bioscience |
|  | 3-carboxy-4-methyl-5-propyl-2-furanpropanoic acid | 86879-39-2 | LGC |
|  | 3-methylhistidine | 368-16-1 | Sigma-Aldrich |
|  | 4-Pyridoxic acid | 82-82-6 | LGC |
|  | 5-(3′,4′-Dihydroxyphenyl)-γ-valerolactone | 21618-92-8 | Cambridge Bioscience |
|  | Acesulfame K | 33665-90-6 | Sigma-Aldrich |
|  | a-carboxyethyl hydroxychroman | 4072-32-6 | Cambridge Bioscience |
|  | Aspartame | 22839-47-0 | Sigma-Aldrich |
|  | Carnitine | 406-76-8 | Cambridge Bioscience |
|  | Daidzein | 486-66-8 | Cambridge Bioscience |
|  | Enterodiol | 80226-00-2 | LGC |
|  | Enterolactone | 78473-71-9 | Sigma-Aldrich |
|  | Epicatechin | 490-46-0 | LGC |
|  | Equol | 531-95-3 | LGC |
|  | Ferulic Acid | 1135-24-6 | Sigma-Aldrich |
|  | Genistein | 446-72-0 | Cambridge Bioscience |
|  | Glycitein | 40957-83-3 | LGC |
|  | Histidine | 71-00-1 | Cambridge Bioscience |
|  | Lysine | 56-87-1 | Sigma-Aldrich |
|  | Methionine | 63-68-3 | Sigma-Aldrich |
|  | N-methylnicotinamide | 114-33-0 | Sigma-Aldrich |
|  | Pantothenic acid | 79-83-4 | Cambridge Bioscience |
|  | Phenylalanine | 63-91-2 | LGC |
|  | Phloretin | 60-82-2 | Cambridge Bioscience |
|  | Resveratrol | 501-36-0 | Sigma-Aldrich |
|  | Riboflavin | 83-88-5 | LGC |
|  | Saccharin | 81-07-2 | Sigma-Aldrich |
|  | Stachydrine | 471-87-4 | Cambridge Bioscience |
|  | Sucralose | 56038-13-2 | Cambridge Bioscience |
|  | D,L-Sulforaphane N-acetyl L-cysteine | 334829-66-2 | LGC |
|  | Trimethylamine N-oxide | 1184-78-7 | Sigma-Aldrich |
|  | Tryptophan | 73-22-3 | LGC |
|  | Urolithin A | 1143-70-0 | Cambridge Bioscience |
|  | Valine | 72-18-4 | Sigma-Aldrich |
| Personal care products | 3-hydroxypropyl mercapturic acid | 23127-40-4 | LGC |
|  | Benzophenone-1 | 131-56-6 | Sigma-Aldrich |
|  | Benzophenone-2 | 131-55-5 | Sigma-Aldrich |
|  | Benzophenone-4 | 4065-45-6 | Sigma-Aldrich |
|  | Bisphenol A | 80-05-7 | Sigma-Aldrich |
|  | Chloroxylenol | 88-04-0 | Sigma-Aldrich |
|  | Butylparaben | 94-26-8 | Sigma-Aldrich |
|  | Ethylparaben | 120-47-8 | Sigma-Aldrich |
|  | Methylparaben | 99-76-3 | Sigma-Aldrich |
|  | Propylparaben | 94-13-3 | Sigma-Aldrich |

**Table S3.** Average daily flow rate for the 1-week study, measured at the wastewater inlet.

| Day | Average daily flow rate (m^3^/day) |
| --- | --- |
| 1 | 14679.23 ± 1185.42 |
| 2 | 14405.13 ± 1359.57 |
| 3 | 14949.96 ± 685.19 |
| 4 | 14625.71 ± 1122.80 |
| 5 | 14927.74 ± 846.15 |
| 6 | 14353.65 ± 1439.13 |
| 7 | 14696.30 ± 1245.29 |

**Table S4.** All isotopically labelled standards added to the internal standard mix, enabling quantification.

| Internal standard | CAS-No | Supplier |
| --- | --- | --- |
| Acetaminophen-d4 | 64315-36-2 | Sigma Aldrich |
| Adenosine 13C5 | 159496-13-6 | LGC |
| Amitriptyline-d3 | 342611-00-1 | Sigma Aldrich |
| Amphetamine-d5 | 65538-33-2 | Sigma Aldrich |
| Atenolol-d7 | 1202865-50-3 | Sigma Aldrich |
| Benzoylecgonine-d8 | 205446-21-5 | Sigma Aldrich |
| Bezafibrate-d6 | 1219802-74-0 | QMX laboratories |
| Bisphenol A-d16 | 96210-87-6 | Sigma Aldrich |
| Caffeine-d9 | 72238-85-8 | Sigma Aldrich |
| Carbamazepine 13C6 | - | Sigma Aldrich |
| Citalopram-d6 | 1190003-26-9 | Sigma Aldrich |
| Cocaethylene-d3 | 36765-30-5 | Sigma Aldrich |
| Cocaine-d3 | 65266-73-1 | Sigma Aldrich |
| Codeine-d6 | 1007844-34-9 | Sigma Aldrich |
| Cortisol-d4 | 73565-87-4 | Sigma Aldrich |
| Cotinine-d3 | 66269-66-7 | Sigma Aldrich |
| Desmethyl diazepam-d5 | 65891-80-7 | Sigma Aldrich |
| Diazepam-d5 | 65854-76-4 | Sigma Aldrich |
| E1-d4 | 53866-34-5 | Sigma Aldrich |
| E2-d4 | 66789-03-5 | Sigma Aldrich |
| EDDP-d3 | 136765-23-6 | Sigma Aldrich |
| Gabapentin-d4 | 1185039-20-6 | Sigma Aldrich |
| Ibuprofen-d3 | 121662-14-4 | Sigma Aldrich |
| Ketamine-d4 | 1246815-97-3 | Sigma Aldrich |
| MDA-d5 | 136765-42-9 | Sigma Aldrich |
| MDMA-d5 | 136765-43-0 | Sigma Aldrich |
| Metformin-d6 | 1185166-01-1 | Sigma Aldrich |
| Methadone-d9 | 1435933-74-6 | Sigma Aldrich |
| Methamphetamine-d5 | 2747917-87-7 | Sigma Aldrich |
| Methylparaben-13C6 | 1581694-95-2 | LGC |
| Metoprolol-d7 | 1219798-61-4 | LGC |
| Mirtazapine-d3 | 1216678-68-0 | Sigma Aldrich |
| Morphine-d3 | 67293-88-3 | Sigma Aldrich |
| Naproxen-d3 | 958293-77-1 | Sigma Aldrich |
| Norketamine-d4 | 1435934-57-8 | Sigma Aldrich |
| Norsertraline 13C6 | 675126-10-0 | Sigma Aldrich |
| Nortriptyline-d3 | 203784-52-5 | Sigma Aldrich |
| Propranolol-d7 | 344298-99-3 | Sigma Aldrich |
| Quetiapine-d8 | 1185247-12-4 | Sigma Aldrich |
| Sertraline-d3 | 1217741-83-7 | Sigma Aldrich |
| Sildenafil-d8 | 951385-68-5 | Sigma Aldrich |
| Verapamil-d7 | 1188265-55-5 | Sigma Aldrich |


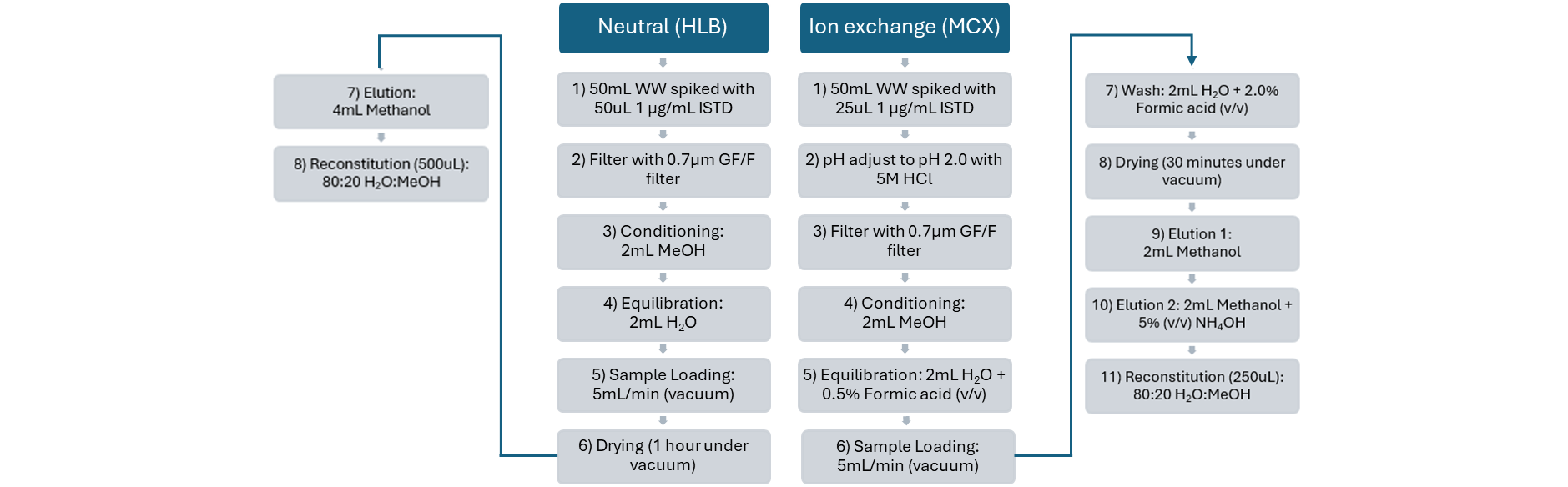


**Figure S1.** Protocols for neutral and ion exchange SPE, used in this manuscript for extraction of BCIs

**Table S5.** MS parameters for all analytes under study within this manuscript, grouped by sub-class

| Class | Analyte | Ionisation Mode | Precursor Ion (m/z) | Cone Voltage (V) | Quantification ion (m/z) | Collision Energy (eV) | Confirmation ion (m/z) | Collision Energy (eV) |
| --- | --- | --- | --- | --- | --- | --- | --- | --- |
| Illicit drugs | 4-methylmethcathinone | ESI^+^ | 178.10 | 10 | 160.10 | 12 | 145.00 | 22 |
|  | Amphetamine | ESI^+^ | 136.10 | 20 | 90.90 | 13 | 119.00 | 9 |
|  | Cocaine | ESI^+^ | 304.30 | 41 | 182.10 | 20 | 82.10 | 28 |
|  | Anhydroecgonine methylester | ESI^+^ | 182.10 | 39 | 118.00 | 23 | 122.10 | 21 |
|  | Benzoylecgonine | ESI^+^ | 290.20 | 38 | 168.10 | 19 | 104.90 | 28 |
|  | Cocaethylene | ESI^+^ | 318.20 | 38 | 196.20 | 20 | 82.10 | 30 |
|  | Heroin | ESI^+^ | 370.20 | 51 | 165.10 | 50 | 268.10 | 29 |
|  | 6-monoacetylmorphine | ESI^+^ | 328.10 | 52 | 165.10 | 39 | 211.10 | 26 |
|  | Ketamine | ESI^+^ | 238.10 | 31 | 125.00 | 27 | 220.10 | 15 |
|  | Norketamine | ESI^+^ | 224.00 | 23 | 125.00 | 27 | 207.10 | 12 |
|  | Methamphetamine | ESI^+^ | 150.10 | 26 | 90.90 | 16 | 119.00 | 10 |
|  | 3,4 – methylenedioxyamphetamine | ESI^+^ | 180.10 | 22 | 163.00 | 10 | 104.90 | 21 |
|  | 3,4 – methylenedioxymethamphetamine | ESI^+^ | 194.10 | 26 | 163.00 | 12 | 104.90 | 24 |
|  | Methylenedioxypyrovalerone | ESI^+^ | 276.10 | 40 | 126.05 | 28 | 135.00 | 25 |
| Lifestyle chemicals | Caffeine | ESI^+^ | 195.10 | 38 | 138.00 | 15 | 110.00 | 23 |
|  | 7-methylxanthine | ESI^+^ | 167.00 | 40 | 124.00 | 18 | 42.00 | 25 |
|  | Paraxanthine | ESI^+^ | 181.00 | 54 | 124.10 | 21 | 69.00 | 35 |
|  | Nicotine | ESI^+^ | 163.10 | 37 | 130.00 | 20 | 117.00 | 24 |
|  | Cotinine | ESI^+^ | 177.10 | 34 | 80.00 | 21 | 98.10 | 22 |
| Pharmaceuticals | 17α-Ethinylestradiol | ESI^-^ | 295.20 | 60 | 144.90 | 45 | 158.80 | 40 |
|  | Acetaminophen | ESI^+^ | 152.10 | 35 | 110.00 | 15 | 92.90 | 21 |
|  | Amitriptyline | ESI^+^ | 278.00 | 40 | 91.10 | 26 | 233.00 | 18 |
|  | Amlodipine | ESI^+^ | 409.00 | 19 | 238.00 | 9 | 294.00 | 7 |
|  | O-des[2-aminoethyl]O-carboxymethyl dehydroamlodipine | ESI^+^ | 422.00 | 45 | 286.00 | 29 | 258.00 | 41 |
|  | Atenolol | ESI^+^ | 267.31 | 38 | 145.10 | 30 | 190.10 | 16 |
|  | Atorvastatin | ESI^-^ | 557.10 | 50 | 278.10 | 45 | 397.05 | 30 |
|  | 2-OH Atorvastatin | ESI^-^ | 573.10 | 51 | 278.00 | 40 | 134.00 | 34 |
|  | Beclomethasone | ESI^+^ | 409.40 | 23 | 391.00 | 10 | 147.00 | 29 |
|  | Betamethasone | ESI^+^ | 393.40 | 22 | 373.30 | 8 | 147.00 | 13 |
|  | Dexamethasone | ESI^+^ | 393.40 | 22 | 373.30 | 8 | 147.00 | 13 |
|  | Bendroflumethiazide | ESI^-^ | 420.00 | 50 | 289.00 | 26 | 328.00 | 25 |
|  | Bezafibrate | ESI^-^ | 360.10 | 30 | 274.00 | 19 | 154.00 | 28 |
|  | Bisoprolol | ESI^+^ | 326.00 | 45 | 116.00 | 18 | 204.00 | 19 |
|  | Budesonide | ESI^+^ | 431.50 | 23 | 413.00 | 10 | 147.00 | 37 |
|  | Buprenorphine | ESI^+^ | 468.40 | 66 | 396.00 | 41 | 414.00 | 35 |
|  | Capecitabine | ESI^+^ | 359.90 | 25 | 243.90 | 11 | 173.80 | 23 |
|  | Carbamazepine | ESI^+^ | 237.00 | 40 | 194.05 | 20 | 179.05 | 38 |
|  | Carbamazepine-10,11-epoxide | ESI^+^ | 253.05 | 39 | 180.10 | 25 | 210.05 | 12 |
|  | 10,11-dihydro-10-hydroxy carbamazepine | ESI^+^ | 255.10 | 20 | 194.05 | 20 | 179.05 | 40 |
|  | Cimetidine | ESI^+^ | 252.86 | 22 | 159.38 | 16 | 211.17 | 10 |
|  | Citalopram | ESI^+^ | 325.30 | 47 | 109.00 | 26 | 262.10 | 18 |
|  | Desmethyl citalopram | ESI^+^ | 311.37 | 46 | 109.00 | 27 | 262.00 | 18 |
|  | Clobazam | ESI^+^ | 301.20 | 45 | 259.20 | 21 | 224.20 | 34 |
|  | Codeine | ESI^+^ | 300.20 | 49 | 215.10 | 25 | 152.10 | 57 |
|  | Norcodeine | ESI^+^ | 286.10 | 46 | 268.20 | 20 | 165.10 | 40 |
|  | Dihydrocodeine | ESI^+^ | 302.10 | 53 | 199.10 | 33 | 128.10 | 60 |
|  | Diazepam | ESI^+^ | 285.00 | 56 | 153.90 | 29 | 221.80 | 27 |
|  | Desmethyl diazepam | ESI^+^ | 271.10 | 54 | 140.00 | 27 | 165.00 | 32 |
|  | Diclofenac | ESI^-^ | 294.00 | 23 | 250.00 | 10 | 214.00 | 20 |
|  | 4-hydroxy diclofenac | ESI^-^ | 310.00 | 30 | 266.00 | 12 | 230.00 | 8 |
|  | Diltiazem | ESI^+^ | 415.10 | 40 | 178.00 | 25 | 310.05 | 25 |
|  | N-desmethyl diltiazem | ESI^+^ | 401.00 | 37 | 178.00 | 21 | 310.00 | 22 |
|  | Donepezil | ESI^+^ | 380.30 | 56 | 362.10 | 23 | 288.00 | 24 |
|  | Duloxetine | ESI^+^ | 298.20 | 13 | 44.00 | 5 | 154.00 | 5 |
|  | Ephedrine | ESI^+^ | 166.10 | 23 | 148.10 | 12 | 133.10 | 21 |
|  | Fexofenadine | ESI^-^ | 500.10 | 33 | 456.10 | 14 | 378.10 | 19 |
|  | Fluoxetine | ESI^+^ | 310.17 | 34 | 44.10 | 10 | 148.11 | 10 |
|  | Norfluoxetine | ESI^+^ | 296.10 | 18 | 134.10 | 6 | 30.00 | 14 |
|  | Gabapentin | ESI^+^ | 172.10 | 30 | 154.10 | 12 | 95.00 | 22 |
|  | Gemfibrozil | ESI^+^ | 251.00 | 21 | 205.00 | 9 | 123.00 | 14 |
|  | Gliclazide | ESI^+^ | 324.05 | 41 | 110.00 | 20 | 127.00 | 20 |
|  | OH-Gliclazide | ESI^+^ | 340.41 | 40 | 110.00 | 26 | 127.00 | 24 |
|  | Ibuprofen | ESI^-^ | 205.10 | 21 | 161.10 | 7 | - | - |
|  | 2-OH ibuprofen | ESI^-^ | 221.00 | 30 | 177.00 | 6 | - | - |
|  | Ifosfamide | ESI^+^ | 261.00 | 40 | 91.95 | 28 | 153.95 | 22 |
|  | Imatinib | ESI^+^ | 494.20 | 57 | 394.00 | 27 | 378.30 | 48 |
|  | Irbesartan | ESI^-^ | 427.05 | 50 | 193.00 | 28 | 121.00 | 65 |
|  | Ketoprofen | ESI^-^ | 253.00 | 15 | 209.00 | 7 | - | - |
|  | Dihydroketoprofen | ESI^-^ | 255.10 | 19 | 211.10 | 8 | 77.00 | 24 |
|  | Lansoprazole | ESI^+^ | 370.20 | 24 | 252.00 | 11 | 119.00 | 17 |
|  | 5-OH Lansoprazole | ESI^+^ | 370.69 | 38 | 236.00 | 27 | 337.00 | 22 |
|  | Lansoprazole Sulfone | ESI^+^ | 386.36 | 36 | 119.00 | 26 | 205.00 | 26 |
|  | Levetiracetam | ESI^+^ | 171.00 | 18 | 126.00 | 16 | 154.00 | 7 |
|  | Lisinopril | ESI^+^ | 406.15 | 38 | 84.00 | 27 | 246.10 | 22 |
|  | Methadone | ESI^+^ | 310.20 | 31 | 265.10 | 15 | 105.10 | 28 |
|  | EDDP | ESI^+^ | 278.20 | 50 | 234.10 | 29 | 249.10 | 24 |
|  | Memantine | ESI^+^ | 180.00 | 35 | 163.00 | 18 | 107.00 | 22 |
|  | Metformin | ESI^+^ | 130.00 | 30 | 60.00 | 15 | 70.95 | 20 |
|  | Guanyl Urea | ESI^+^ | 102.90 | 24 | 60.00 | 10 | 85.90 | 8 |
|  | Methotrexate | ESI^+^ | 455.10 | 40 | 308.10 | 20 | 175.05 | 35 |
|  | Methylprednisolone | ESI^+^ | 375.30 | 23 | 357.20 | 10 | 161.00 | 23 |
|  | Metoprolol | ESI^+^ | 268.33 | 42 | 116.09 | 20 | 121.10 | 22 |
|  | O-desmethyl metoprolol | ESI^+^ | 254.00 | 32 | 116.00 | 19 | 177.00 | 20 |
|  | Mirtazapine | ESI^+^ | 266.13 | 44 | 195.03 | 26 | 71.99 | 18 |
|  | Morphine | ESI^+^ | 286.00 | 51 | 165.00 | 36 | 152.00 | 34 |
|  | Dihydromorphine | ESI^+^ | 288.20 | 28 | 185.00 | 42 | 213.00 | 32 |
|  | Normorphine | ESI^+^ | 272.00 | 50 | 165.00 | 43 | 152.10 | 45 |
|  | Naproxen | ESI^-^ | 229.00 | 20 | 185.00 | 7 | 170.00 | 13 |
|  | O-desmethyl naproxen | ESI^-^ | 215.00 | 30 | 171.00 | 12 | 169.00 | 30 |
|  | Nicorandil | ESI^+^ | 212.00 | 18 | 136.00 | 9 | 79.00 | 22 |
|  | Nitrazepam | ESI^+^ | 282.30 | 42 | 236.10 | 24 | 180.20 | 35 |
|  | Norephedrine | ESI^+^ | 152.21 | 23 | 134.10 | 10 | 117.10 | 16 |
|  | Nortriptyline | ESI^+^ | 264.10 | 33 | 91.00 | 23 | 233.10 | 16 |
|  | 10-OH nortriptyline | ESI^+^ | 280.30 | 26 | 262.20 | 13 | 216.20 | 21 |
|  | Oseltamivir | ESI^+^ | 313.40 | 23 | 166.00 | 19 | 208.00 | 13 |
|  | Oxazepam | ESI^+^ | 286.90 | 38 | 240.80 | 22 | 268.80 | 14 |
|  | Prednisolone | ESI^+^ | 361.00 | 23 | 147.00 | 24 | 121.00 | 10 |
|  | Pregabalin | ESI^+^ | 160.00 | 32 | 142.00 | 11 | 124.50 | 14 |
|  | N-methyl pregabalin | ESI^+^ | 174.25 | 25 | 97.00 | 17 | 138.00 | 17 |
|  | Propranolol | ESI^+^ | 260.18 | 42 | 116.09 | 16 | 183.09 | 18 |
|  | Quetiapine | ESI^+^ | 384.10 | 50 | 253.05 | 21 | 221.10 | 40 |
|  | 7-OH Quetiapine | ESI^+^ | 400.00 | 39 | 269.00 | 26 | 237.00 | 37 |
|  | Ranitidine | ESI^+^ | 315.90 | 26 | 176.00 | 17 | 123.90 | 24 |
|  | Ranitidine N-oxide | ESI^+^ | 331.00 | 24 | 176.00 | 18 | 270.00 | 10 |
|  | Risperidone | ESI^+^ | 411.00 | 49 | 190.90 | 30 | 109.90 | 51 |
|  | Salbutamol | ESI^+^ | 240.09 | 30 | 148.00 | 18 | 160.01 | 12 |
|  | Sertraline | ESI^+^ | 306.00 | 23 | 159.00 | 27 | 275.00 | 10 |
|  | Norsertraline | ESI^+^ | 291.80 | 25 | 158.60 | 23 | 128.80 | 22 |
|  | Sildenafil | ESI^+^ | 475.30 | 60 | 99.90 | 31 | 282.90 | 43 |
|  | N-desmethyl sildenafil | ESI^+^ | 461.00 | 50 | 283.00 | 40 | 311.00 | 32 |
|  | Simvastatin | ESI^+^ | 419.20 | 29 | 199.10 | 12 | 285.10 | 10 |
|  | Sitagliptin | ESI^+^ | 407.80 | 46 | 234.90 | 19 | 192.90 | 26 |
|  | Tamoxifen | ESI^+^ | 372.15 | 50 | 72.00 | 25 | 129.00 | 28 |
|  | Temazepam | ESI^+^ | 301.10 | 37 | 255.10 | 21 | 283.10 | 14 |
|  | Topiramate | ESI^-^ | 338.00 | 39 | 78.00 | 27 | 96.00 | 38 |
|  | Tramadol | ESI^+^ | 264.20 | 28 | 246.20 | 11 | 58.00 | 16 |
|  | O-desmethyl tramadol | ESI^+^ | 250.15 | 30 | 58.00 | 18 | 232.10 | 10 |
|  | N-desmethyl tramadol | ESI^+^ | 250.10 | 25 | 44.00 | 12 | 232.10 | 8 |
|  | Valsartan | ESI^-^ | 434.00 | 35 | 179.10 | 25 | 350.10 | 20 |
|  | 4-hydroxy valsartan | ESI^-^ | 450.52 | 37 | 350.00 | 22 | 179.00 | 22 |
|  | Venlafaxine | ESI^+^ | 278.15 | 27 | 58.10 | 40 | 260.10 | 12 |
|  | Desvenlafaxine | ESI^+^ | 264.00 | 25 | 107.10 | 24 | 246.30 | 20 |
|  | Verapamil | ESI^+^ | 455.20 | 55 | 165.00 | 31 | 303.10 | 26 |
|  | p-O-desmethyl verapamil | ESI^+^ | 441.58 | 37 | 151.00 | 33 | 291.00 | 26 |
|  | Zolpidem | ESI^+^ | 308.00 | 56 | 235.00 | 30 | 263.00 | 20 |
| Human markers | 1,4‑methylimidazoleacetic acid | ESI^+^ | 141.00 | 23 | 95.00 | 15 | 68.00 | 26 |
|  | 2’-deoxyguanosine | ESI^+^ | 268.20 | 13 | 152.00 | 9 | 135.00 | 33 |
|  | 2'-deoxyinosine | ESI^+^ | 253.20 | 17 | 137.00 | 9 | 43.00 | 29 |
|  | 3-nitro-L-tyrosine | ESI^+^ | 227.00 | 27 | 181.00 | 13 | 117.00 | 22 |
|  | 3-chloro-L-tyrosine | ESI^+^ | 216.00 | 26 | 170.00 | 13 | 199.00 | 10 |
|  | 5-Hydroxymethyl-2'-deoxyuridine | ESI^+^ | 259.20 | 15 | 125.00 | 17 | 143.00 | 7 |
|  | 5-hydroxyindole acetic acid | ESI^+^ | 192.00 | 31 | 146.00 | 25 | 118.50 | 28 |
|  | 5-methyl-2'-deoxycytidine | ESI^+^ | 242.20 | 19 | 126.00 | 11 | 109.00 | 35 |
|  | 8-hydroxyguanosine | ESI^+^ | 300.00 | 30 | 168.00 | 13 | - | - |
|  | 8-hydroxyguanine | ESI^+^ | 168.00 | 40 | 112.00 | 18 | 140.00 | 15 |
|  | Adenosine | ESI^+^ | 268.00 | 42 | 136.00 | 19 | 119.00 | 43 |
|  | Androstenedione | ESI^+^ | 287.00 | 41 | 97.00 | 21 | 109.00 | 25 |
|  | Asymmetric dimethyl arginine | ESI^+^ | 203.10 | 33 | 70.10 | 23 | 46.20 | 16 |
|  | Cortisol | ESI^+^ | 363.20 | 38 | 121.00 | 25 | 97.00 | 28 |
|  | Cortisone | ESI^+^ | 361.30 | 43 | 163.10 | 24 | 121.00 | 32 |
|  | Creatinine | ESI^+^ | 114.00 | 30 | 44.00 | 15 | 86.10 | 11 |
|  | Deoxyadenosine | ESI^+^ | 252.00 | 25 | 136.00 | 14 | 117.00 | 20 |
|  | Dihydrobiopterin | ESI^+^ | 240.00 | 33 | 196.00 | 13 | 165.00 | 19 |
|  | Dihydrotestosterone | ESI^+^ | 291.40 | 38 | 255.00 | 15 | 159.00 | 24 |
|  | Estradiol | ESI^-^ | 271.10 | 60 | 183.00 | 40 | 144.90 | 45 |
|  | Estrone | ESI^-^ | 269.00 | 55 | 145.00 | 40 | - | - |
|  | Formiminoglutamic acid | ESI^+^ | 175.00 | 30 | 157.00 | 10 | 83.60 | 20 |
|  | Hippuric acid | ESI^+^ | 180.00 | 20 | 105.00 | 11 | 77.00 | 28 |
|  | HNE-MA | ESI^-^ | 318.20 | 32 | 171.00 | 22 | 189.00 | 14 |
|  | Hydroxymethyl uracil | ESI^+^ | 143.00 | 18 | 124.80 | 8 | 82.00 | 13 |
|  | Indoxyl sulfate | ESI^-^ | 212.00 | 30 | 132.00 | 20 | 80.50 | 16 |
|  | Inosine | ESI^+^ | 269.20 | 17 | 137.20 | 9 | 110.00 | 40 |
|  | Nε-(1-Carboxyethyl)-L-lysine | ESI^+^ | 219.00 | 32 | 84.00 | 21 | 130.00 | 12 |
|  | Nε-(1-Carboxymethyl)-L-lysine | ESI^+^ | 205.00 | 32 | 84.00 | 19 | 130.00 | 11 |
|  | Neopterin | ESI^+^ | 254.10 | 35 | 190.00 | 20 | 206.00 | 16 |
|  | Phenylacetyl L-glutamine | ESI^+^ | 265.00 | 28 | 130.00 | 17 | 84.00 | 28 |
|  | Pterin | ESI^+^ | 164.10 | 37 | 118.90 | 19 | 147.00 | 15 |
|  | Progesterone | ESI^+^ | 315.00 | 37 | 97.00 | 22 | 109.00 | 24 |
|  | Pyroglutamic acid | ESI^+^ | 130.10 | 30 | 84.00 | 13 | 56.00 | 21 |
|  | Testosterone | ESI^+^ | 289.00 | 40 | 97.00 | 24 | 109.00 | 28 |
| Food | 1-methyl-2-pyridone-5-carboxamide | ESI^+^ | 153.00 | 44 | 108.00 | 20 | 122.00 | 17 |
|  | 1-methylhistidine | ESI^+^ | 170.00 | 22 | 124.00 | 14 | 109.00 | 14 |
|  | 3-carboxy-4-methyl-5-propyl-2-furanpropanoic acid | ESI^+^ | 241.10 | 14 | 223.00 | 7 | 181.00 | 16 |
|  | 3-methylhistidine | ESI^+^ | 170.00 | 28 | 96.00 | 19 | 126.00 | 11 |
|  | 4-Pyridoxic acid | ESI^+^ | 184.00 | 36 | 148.00 | 19 | 166.00 | 12 |
|  | 5-(3′,4′-Dihydroxyphenyl)-γ-valerolactone | ESI^+^ | 209.00 | 34 | 149.00 | 12 | 131.00 | 20 |
|  | Acesulfame K | ESI^-^ | 162.00 | 21 | 82.00 | 13 | 78.00 | 16 |
|  | a-CEHC | ESI^-^ | 276.80 | 40 | 233.00 | 14 | 164.00 | 17 |
|  | Aspartame | ESI^+^ | 295.00 | 27 | 120.00 | 27 | 180.00 | 14 |
|  | Carnitine | ESI^+^ | 162.00 | 33 | 102.80 | 16 | 60.00 | 15 |
|  | Daidzein | ESI^-^ | 253.00 | 51 | 91.00 | 33 | 224.00 | 26 |
|  | Enterodiol | ESI^-^ | 301.00 | 46 | 253.00 | 21 | 106.00 | 35 |
|  | Enterolactone | ESI^-^ | 297.00 | 49 | 107.00 | 24 | 253.00 | 20 |
|  | Epicatechin | ESI^-^ | 289.00 | 40 | 245.00 | 16 | 109.00 | 22 |
|  | Equol | ESI^-^ | 241.00 | 30 | 121.00 | 13 | 119.00 | 17 |
|  | Ferulic Acid | ESI^-^ | 193.00 | 21 | 134.00 | 12 | 149.00 | 10 |
|  | Genistein | ESI^-^ | 268.80 | 52 | 133.00 | 31 | 181.00 | 22 |
|  | Glycitein | ESI^-^ | 283.00 | 50 | 268.00 | 18 | 240.00 | 27 |
|  | Histidine | ESI^+^ | 156.00 | 26 | 110.00 | 13 | 82.80 | 24 |
|  | Lysine | ESI^+^ | 147.00 | 22 | 84.00 | 14 | 130.00 | 9 |
|  | Methionine | ESI^+^ | 150.00 | 25 | 103.90 | 10 | 61.00 | 19 |
|  | N-methylnicotinamide | ESI^+^ | 137.10 | 34 | 80.00 | 20 | 108.00 | 15 |
|  | Pantothenic acid | ESI^-^ | 218.00 | 35 | 88.00 | 13 | 146.00 | 14 |
|  | Phenylalanine | ESI^+^ | 166.00 | 25 | 120.00 | 13 | 103.00 | 25 |
|  | Phloretin | ESI^-^ | 273.10 | 41 | 167.00 | 18 | 119.00 | 24 |
|  | Resveratrol | ESI^-^ | 227.00 | 30 | 185.00 | 23 | 159.00 | 18 |
|  | Riboflavin | ESI^+^ | 377.00 | 40 | 243.00 | 25 | 172.00 | 39 |
|  | Saccharin | ESI^-^ | 181.90 | 41 | 105.90 | 18 | 42.00 | 22 |
|  | Stachydrine | ESI^+^ | 144.00 | 32 | 58.00 | 23 | 84.00 | 26 |
|  | Sucralose | ESI^-^ | 396.60 | 32 | 360.60 | 11 | 35.00 | 14 |
|  | D,L-Sulforaphane N-acetyl L-cysteine | ESI^+^ | 341.00 | 24 | 114.00 | 24 | 178.00 | 13 |
|  | Trimethylamine N-oxide | ESI^+^ | 76.00 | 36 | 59.00 | 10 | 58.00 | 15 |
|  | Tryptophan | ESI^+^ | 205.00 | 26 | 146.00 | 16 | 118.00 | 27 |
|  | Urolithin A | ESI^-^ | 227.00 | 32 | 198.00 | 29 | 182.00 | 34 |
|  | Valine | ESI^+^ | 118.00 | 20 | 72.00 | 9 | 55.00 | 18 |
| Personal care products | 3-hydroxypropyl mercapturic acid | ESI^+^ | 222.00 | 23 | 91.00 | 25 | 163.00 | 14 |
|  | Benzophenone-1 | ESI^-^ | 213.00 | 36 | 90.80 | 25 | 134.80 | 20 |
|  | Benzophenone-2 | ESI^-^ | 244.90 | 32 | 134.90 | 13 | 108.90 | 20 |
|  | Benzophenone-4 | ESI^-^ | 307.00 | 42 | 211.10 | 35 | 227.10 | 24 |
|  | Bisphenol A | ESI^-^ | 227.30 | 40 | 212.10 | 22 | 132.70 | 25 |
|  | Chloroxylenol | ESI^-^ | 155.00 | 33 | 35.00 | 14 | 119.00 | 16 |
|  | Butylparaben | ESI^-^ | 193.10 | 34 | 91.80 | 25 | 136.00 | 16 |
|  | Ethylparaben | ESI^-^ | 164.90 | 26 | 91.90 | 20 | 136.60 | 14 |
|  | Methylparaben | ESI^-^ | 150.80 | 34 | 91.80 | 20 | 135.80 | 14 |
|  | Propylparaben | ESI^-^ | 179.00 | 34 | 91.80 | 25 | 136.00 | 16 |

**Table S6.** MS parameters for all isotopically labelled analytes under study, used for quantification, within this manuscript, grouped by sub-class

| Analyte | Ionisation Mode | Precursor Ion (m/z) | Cone Voltage (V) | Quantification ion (m/z) | Collision Energy (eV) |
| --- | --- | --- | --- | --- | --- |
| Acetaminophen-d4 | ESI^+^ | 156.10 | 35 | 114.00 | 15 |
| Acetaminophen-d4 | ESI^-^ | 154.00 | 38 | 111.00 | 19 |
| Adenosine 13C5 | ESI^+^ | 273.00 | 36 | 136.00 | 18 |
| Amitriptyline-d3 | ESI^+^ | 281.50 | 40 | 90.90 | 25 |
| Amphetamine-d5 | ESI^+^ | 141.07 | 20 | 92.83 | 14 |
| Atenolol-d7 | ESI^+^ | 274.31 | 44 | 145.09 | 30 |
| Benzoylecgonine-d8 | ESI^+^ | 298.20 | 38 | 171.10 | 19 |
| Bezafibrate-d6 | ESI^-^ | 366.00 | 30 | 274.00 | 19 |
| Bisphenol A-d16 | ESI^-^ | 241.05 | 40 | 223.10 | 20 |
| Caffeine-d9 | ESI^+^ | 204.10 | 38 | 144.00 | 20 |
| Carbamazepine 13C6 | ESI^+^ | 243.10 | 40 | 200.05 | 20 |
| Citalopram-d6 | ESI^+^ | 331.20 | 47 | 109.00 | 28 |
| Cocaethylene-d3 | ESI^+^ | 321.20 | 40 | 199.07 | 22 |
| Cocaine-d3 | ESI^+^ | 307.30 | 41 | 185.10 | 20 |
| Codeine-d6 | ESI^+^ | 306.20 | 52 | 218.10 | 28 |
| Cortisol-d4 | ESI^+^ | 367.20 | 37 | 121.00 | 25 |
| Cotinine-d3 | ESI^+^ | 180.10 | 44 | 80.00 | 24 |
| Desmethyl diazepam-d5 | ESI^+^ | 276.00 | 36 | 140.00 | 30 |
| Diazepam-d5 | ESI^+^ | 289.90 | 56 | 198.00 | 34 |
| E1-d4 | ESI^-^ | 273.10 | 55 | 147.00 | 40 |
| E2-d4 | ESI^-^ | 275.10 | 60 | 147.00 | 40 |
| EDDP-d3 | ESI^+^ | 281.20 | 50 | 234.10 | 29 |
| Gabapentin-d4 | ESI^+^ | 176.00 | 33 | 158.10 | 16 |
| Ibuprofen-d3 | ESI^-^ | 208.00 | 20 | 164.00 | 6 |
| Ketamine-d4 | ESI^+^ | 242.10 | 31 | 129.10 | 27 |
| MDA-d5 | ESI^+^ | 185.10 | 21 | 168.10 | 11 |
| MDMA-d5 | ESI^+^ | 199.10 | 28 | 165.00 | 13 |
| Metformin-d6 | ESI^+^ | 136.05 | 30 | 77.00 | 19 |
| Methadone-d9 | ESI^+^ | 319.30 | 31 | 268.20 | 15 |
| Methamphetamine-d5 | ESI^+^ | 155.10 | 28 | 91.78 | 18 |
| Methylparaben-13C6 | ESI^-^ | 156.90 | 30 | 97.90 | 20 |
| Metoprolol-d7 | ESI^+^ | 275.39 | 44 | 123.12 | 20 |
| Mirtazapine-d3 | ESI^+^ | 269.00 | 35 | 194.90 | 25 |
| Morphine-d3 | ESI^+^ | 289.10 | 53 | 152.10 | 56 |
| Naproxen-d3 | ESI^-^ | 232.00 | 15 | 188.00 | 8 |
| Norketamine-d4 | ESI^+^ | 228.10 | 32 | 128.91 | 28 |
| Norsertraline 13C6 | ESI^+^ | 298.00 | 15 | 159.00 | 24 |
| Nortriptyline-d3 | ESI^+^ | 267.10 | 40 | 191.00 | 20 |
| Propranolol-d7 | ESI^+^ | 267.00 | 40 | 188.80 | 18 |
| Quetiapine-d8 | ESI^+^ | 392.10 | 50 | 258.10 | 23 |
| Sertraline-d3 | ESI^+^ | 309.05 | 23 | 159.00 | 27 |
| Sildenafil-d8 | ESI^+^ | 482.90 | 60 | 108.00 | 31 |
| Verapamil-d7 | ESI^+^ | 462.10 | 56 | 165.00 | 31 |

**Figure S2.** TIC scan of all analytes (100ng/mL) analysed via ESI+ where MPA: 80:20 water:methanol with 2mM ammonium formate and 0.1% (v/v) formic acid (pH 2.95) MPB: 100% methanol. The gradient is indicated by the red-dashed line: 0min (0%B), 3.5min(4%B), 21min(100%B), 28min(100%B), 28.1min(0%B), 35min(0%B). Total run time 34.5 minutes.

**Figure S3.** TIC scan of all analytes (200ng/mL) analysed via ESI- MPA: 80:20 water:methanol with 1mM Ammonium Fluoride MPB: 95:5 Methanol:Water with 1mM Ammonium Fluoride. The gradient is indicated by the red-dashed line: 0min (0%B), 0.5min(0%B), 2.5min(60%B), 8min(100%B), 14min(100%B), 14.1min(0%B), 22.5min(0%B). Total run time 22.5 minutes.

**Table S7.** Instrument performance of all analytes, quantified by UHPLC-MS/MS, following the sample procedure detailed in section 2. Where RT = retention time, RRT = relative retention time, IDL = instrument detection limit and IQL = instrument quantification limit. (MDMA-d5 = 3,4 – Methylenedioxymethamphetamine-d5, EDDP = 2-ethylidene-1,5-dimethyl-3,3-diphenylpyrrolidine)

| Class | Compound | ISTD | ESI mode | Instrumental Performance | | | | | | | | |
| --- | --- | --- | --- | --- | --- | --- | --- | --- | --- | --- | --- | --- |
|  |  |  |  | RT (min) | RRT | Concentration range / µgL^-1^ | Ion ratio | r^2^ | IDL / µgL^-1^ | IQL / µgL^-1^ | Accuracy (%) | Precision (%) |
| Illicit drugs | 4-methylmethcathinone | Amphetamine-d5 | ESI+ | 10.03 ± 0.08 | 1.19 ± 0.02 | IQL – 2000 | 1.84 ± 0.45 | 0.999 | 0.01 | 0.03 | 109.5 | 19.8 |
|  | Amphetamine | Amphetamine-d5 | ESI+ | 8.33 ± 0.16 | 1.01 ± 0.01 | IQL – 1000 | 1.20 ± 0.18 | 0.999 | 0.02 | 0.05 | 87.9 | 7.9 |
|  | Cocaine | Cocaine-d3 | ESI+ | 11.42 ± 0.07 | 1.00 ± 0.01 | IQL – 1000 | 2.40 ± 0.05 | 0.999 | 0.002 | 0.01 | 99.3 | 4.4 |
|  | Anhydroecgonine methylester | Cotinine-d3 | ESI+ | 3.29 ± 0.09 | 1.02 ± 0.03 | IQL – 750 | 1.22 ± 0.03 | 0.998 | 0.001 | 0.003 | 96.4 | 4.1 |
|  | Benzoylecgonine | Benzoylecgonine-d8 | ESI+ | 10.40 ± 0.02 | 1.01 ± 0.01 | IQL – 2000 | 1.83 ± 0.06 | 0.999 | 0.01 | 0.04 | 106.6 | 3.8 |
|  | Cocaethylene | Cocaethylene-d3 | ESI+ | 12.77 ± 0.05 | 1.00 ± 0.01 | IQL – 1000 | 2.04 ± 0.09 | 0.999 | 0.002 | 0.001 | 105.3 | 3.1 |
|  | Heroin | Codeine-d6 | ESI+ | 10.94 ± 0.07 | 2.30 ± 0.13 | IQL – 750 | 1.67 ± 1.23 | 0.999 | 0.1 | 0.4 | 30.0 | 7.6 |
|  | 6-monoacetylmorphine | Ketamine-d4 | ESI+ | 7.27 ± 0.13 | 0.70 ± 0.01 | IQL – 500 | 1.39 ± 0.03 | 1.000 | 0.01 | 0.02 | 100.2 | 7.5 |
|  | Ketamine | Ketamine-d4 | ESI+ | 10.35 ± 0.06 | 1.01 ± 0.12 | IQL – 1000 | 3.03 ± 0.03 | 1.000 | 0.002 | 0.01 | 97.8 | 3.2 |
|  | Norketamine | Norketamine-d4 | ESI+ | 10.39 ± 0.06 | 1.01 ± 0.12 | IQL – 750 | 0.97 ± 0.02 | 1.000 | 0.01 | 0.03 | 91.6 | 1.9 |
|  | Methamphetamine | Methamphetamine-d5 | ESI+ | 8.60 ± 0.15 | 1.01 ± 0.01 | IQL – 2000 | 2.13 ± 0.13 | 1.000 | 0.003 | 0.01 | 99.1 | 7.6 |
|  | 3,4 – Methylenedioxyamphetamine | MDMA-d5 | ESI+ | 8.86 ± 0.10 | 1.01 ± 0.01 | IQL – 500 | 1.95 ± 0.22 | 0.999 | 0.01 | 0.04 | 96.8 | 6.5 |
|  | MDMA | MDMA-d5 | ESI+ | 8.87 ± 0.06 | 1.01 ± 0.001 | IQL – 2000 | 1.76 ± 0.10 | 1.000 | 0.01 | 0.04 | 100.5 | 4.8 |
|  | Methylenedioxypyrovalerone | Benzoylecgonine-d8 | ESI+ | 12.06 ± 0.07 | 1.17 ± 0.01 | IQL – 750 | 1.42 ± 0.02 | 1.000 | 0.001 | 0.004 | 99.2 | 5.8 |
| Lifestyle chemicals | Caffeine | Caffeine-d9 | ESI+ | 9.06 ± 0.02 | 1.01 ± 0.05 | IQL – 1000 | 2.14 ± 0.18 | 0.999 | 0.09 | 0.3 | 107.3 | 3.2 |
|  | 7-methylxanthine | Caffeine-d9 | ESI+ | 3.57 ± 0.07 | 0.40 ± 0.01 | IQL – 750 | 4.84 ± 0.33 | 1.000 | 0.03 | 0.09 | 84.5 | 5.3 |
|  | Paraxanthine | Caffeine-d9 | ESI+ | 6.17 ± 0.04 | 0.60 ± 0.004 | IQL – 2000 | 10.34 ± 1.22 | 0.999 | 0.09 | 0.3 | 103.9 | 3.2 |
|  | Nicotine | Cotinine-d3 | ESI+ | 2.89 ± 0.16 | 0.89 ± 0.01 | IQL – 1500 | 1.15 ± 0.08 | 0.999 | 0.02 | 0.07 | 100.1 | 7.8 |
|  | Cotinine | Cotinine-d3 | ESI+ | 3.23 ± 0.01 | 1.00 ± 0.002 | IQL – 1000 | 2.62 ± 0.14 | 0.999 | 0.002 | 0.01 | 97.5 | 3.2 |
| Pharmaceuticals | 17α-Ethinylestradiol | Estradiol-d4 | ESI- | 9.55 ± 0.03 | 0.99 ± 0.002 | IQL – 1000 | 2.91 ± 1.86 | 0.999 | 0.2 | 0.6 | 91.8 | 4.0 |
|  | Acetaminophen | Acetaminophen-d4 | ESI+ | 5.06 ± 0.07 | 1.01 ± 0.004 | IQL – 1500 | 4.79 ± 0.16 | 1.000 | 0.04 | 0.1 | 91.6 | 2.4 |
|  | Amitriptyline | Amitriptyline-d3 | ESI+ | 17.03 ± 0.04 | 1.00 ± 0.001 | IQL – 500 | 1.72 ± 0.16 | 1.000 | 0.002 | 0.02 | 112.9 | 9.8 |
|  | Amlodipine | Sertraline-d3 | ESI+ | 17.44 ± 0.17 | 0.96 ± 0.002 | IQL – 1000 | 1.05 ± 0.06 | 0.999 | 0.001 | 0.003 | 85.5 | 4.0 |
|  | O-des[2-aminoethyl]O-carboxymethyl dehydroamlodipine | Carbamazepine 13C6 | ESI+ | 17.54 ± 0.17 | 1.09 ± 0.002 | IQL – 750 | 1.86 ± 0.05 | 0.998 | 0.04 | 0.1 | 85.7 | 8.3 |
|  | Atenolol | Atenolol-d7 | ESI+ | 3.77 ± 0.06 | 1.01 ± 0.01 | IQL – 2000 | 1.12 ± 0.10 | 0.999 | 0.02 | 0.04 | 109.4 | 2.7 |
|  | Atorvastatin | Ibuprofen-d3 | ESI- | 9.70 ± 0.01 | 0.92 ± 0.001 | IQL – 500 | 1.13 ± 0.09 | 0.999 | 0.1 | 0.1 | 107.0 | 3.1 |
|  | 2-OH Atorvastatin | Ibuprofen-d3 | ESI- | 9.67 ± 0.01 | 0.89 ± 0.001 | IQL – 500 | 2.15 ± 0.09 | 0.999 | 0.03 | 0.07 | 136.9 | 6.3 |
|  | Beclomethasone | Cortisol-d4 | ESI+ | 17.87 ± 0.15 | 1.08 ± 0.002 | IQL – 1000 | 4.55 ± 0.38 | 1.000 | 0.01 | 0.03 | 90.0 | 3.4 |
|  | Betamethasone / Dexamethasone | Cortisol-d4 | ESI+ | 17.61 ± 0.16 | 1.07 ± 0.002 | IQL – 1000 | 2.57 ± 0.23 | 1.000 | 0.01 | 0.02 | 114.8 | 5.4 |
|  | Bendroflumethiazide | Estrone-d4 | ESI- | 7.76 ± 0.02 | 0.8 ± 0.002 | IQL – 500 | 1.94 ± 0.16 | 0.999 | 0.1 | 0.3 | 87.6 | 12.9 |
|  | Bezafibrate | Bezafibrate-d6 | ESI- | 8.51 ± 0.01 | 1.00 ± 0.001 | IQL – 2000 | 2.29 ± 0.09 | 1.000 | 0.04 | 0.1 | 103.3 | 7.7 |
|  | Bisoprolol | Carbamazepine 13C6 | ESI+ | 13.48 ± 0.11 | 0.84 ± 0.01 | IQL – 600 | 68.51 ± 4.56 | 0.998 | 0.003 | 0.01 | 82.8 | 5.7 |
|  | Budesonide | Cortisol-d4 | ESI+ | 20.09 ± 0.13 | 1.22 ± 0.01 | IQL – 500 | 2.22 ± 0.14 | 0.998 | 0.01 | 0.02 | 99.7 | 9.2 |
|  | Buprenorphine | Cocaine-d3 | ESI+ | 14.73 ± 0.14 | 1.29 ± 0.01 | IQL – 1000 | 1.15 ± 0.06 | 0.999 | 0.01 | 0.03 | 118.4 | 8.2 |
|  | Capecitabine | Desmethyl Diazepam-d5 | ESI+ | 16.05 ± 0.01 | 0.87 ± 0.001 | IQL – 600 | 2.59 ± 0.29 | 1.000 | 0.01 | 0.02 | 87.4 | 7.8 |
|  | Carbamazepine | Carbamazepine 13C6 | ESI+ | 16.08 ± 0.01 | 1.00 ± 0.02 | IQL – 2000 | 10.36 ± 0.32 | 1.000 | 0.003 | 0.001 | 104.0 | 3.0 |
|  | Carbamazepine-10,11-epoxide | Carbamazepine 13C6 | ESI+ | 13.83 ± 0.10 | 1.53 ± 0.01 | IQL – 750 | 2.29 ± 0.13 | 0.999 | 0.07 | 0.2 | 145.2 | 18.6 |
|  | 10,11-dihydro-10-hydroxy carbamazepine | Codeine-d6 | ESI+ | 13.84 ± 0.10 | 2.88 ± 0.05 | IQL – 750 | 15.13 ± 2.78 | 0.998 | 0.1 | 0.4 | 102.6 | 10.7 |
|  | Cimetidine | Atenolol-d7 | ESI+ | 4.29 ± 0.08 | 1.13 ± 0.01 | IQL – 500 | 4.22 ± 0.41 | 0.998 | 0.02 | 0.05 | 93.2 | 15.0 |
|  | Citalopram | Citalopram-d6 | ESI+ | 14.53 ± 0.06 | 1.00 ± 0.12 | IQL – 2000 | 4.94 ± 0.06 | 1.000 | 0.001 | 0.004 | 92.8 | 4.5 |
|  | Desmethyl citalopram | Citalopram-d6 | ESI+ | 14.65 ± 0.07 | 1.01 ± 0.13 | IQL – 1000 | 3.77 ± 0.11 | 0.999 | 0.001 | 0.002 | 81.7 | 4.6 |
|  | Clobazam | Carbamazepine 13C6 | ESI+ | 16.95 ± 0.16 | 1.05 ± 0.001 | IQL – 750 | 3.33 ± 0.14 | 0.997 | 0.001 | 0.003 | 92.8 | 4.7 |
|  | Codeine | Codeine-d6 | ESI+ | 4.75 ± 0.08 | 1.02 ± 0.02 | IQL – 1000 | 1.74 ± 0.08 | 0.999 | 0.01 | 0.03 | 92.5 | 3.5 |
|  | Norcodeine | Codeine-d6 | ESI+ | 5.29 ± 0.09 | 1.10 ± 0.01 | IQL – 500 | 0.87 ± 0.06 | 0.998 | 0.06 | 0.2 | 88.9 | 3.2 |
|  | Dihydrocodeine | Codeine-d6 | ESI+ | 4.53 ± 0.09 | 0.97 ± 0.17 | IQL – 500 | 1.95 ± 0.19 | 0.999 | 0.01 | 0.03 | 94.7 | 3.7 |
|  | Diazepam | Diazepam-d5 | ESI+ | 18.87 ± 0.14 | 1.00 ± 0.001 | IQL – 750 | 2.02 ± 0.05 | 0.999 | 0.002 | 0.01 | 118.0 | 2.6 |
|  | Desmethyl diazepam | Diazepam-d5 | ESI+ | 18.44 ± 0.14 | 0.98 ± 0.001 | IQL – 500 | 2.71 ± 0.09 | 0.997 | 0.007 | 0.02 | 93.7 | 5.2 |
|  | Diclofenac | Naproxen-d3 | ESI- | 9.29 ± 0.03 | 1.10 ± 0.004 | IQL – 2000 | 21.27 ± 9.86 | 0.998 | 0.03 | 0.08 | 97.7 | 3.2 |
|  | 4-hydroxy diclofenac | Naproxen-d3 | ESI- | 8.13 ± 0.03 | 0.96 ± 0.003 | IQL – 500 | 5.77 ± 0.73 | 0.997 | 0.09 | 0.3 | 88.1 | 6.4 |
|  | Diltiazem | Cocaethylene-d3 | ESI+ | 15.49 ± 0.16 | 1.21 ± 0.01 | IQL – 750 | 28.11 ± 1.09 | 0.999 | 0.001 | 0.003 | 82.1 | 6.2 |
|  | N-desmethyl diltiazem | Quetiapine-d8 | ESI+ | 15.59 ± 0.05 | 1.04 ± 0.001 | IQL – 750 | 10.63 ± 0.46 | 1.000 | 0.003 | 0.01 | 110.3 | 4.0 |
|  | Donepezil | Methadone-d9 | ESI+ | 13.50 ± 0.10 | 0.81 ± 0.004 | IQL – 750 | 1.37 ± 0.08 | 0.999 | 0.01 | 0.03 | 102.9 | 4.5 |
|  | Duloxetine | EDDP-d3 | ESI+ | 16.90 ± 0.17 | 1.17 ± 0.01 | IQL – 750 | 1.34 ± 0.10 | 0.999 | 0.01 | 0.02 | 96.2 | 8.8 |
|  | Ephedrine | Cocaine-d3 | ESI+ | 6.25 ± 0.12 | 0.55 ± 0.01 | IQL – 1500 | 7.02 ± 0.26 | 1.000 | 0.01 | 0.03 | 103.8 | 15.3 |
|  | Fexofenadine | Ibuprofen-d3 | ESI- | 8.48 ± 0.11 | 0.81 ± 0.01 | IQL – 500 | 1.49 ± 0.09 | 0.998 | 0.2 | 0.6 | 93.6 | 2.6 |
|  | Fluoxetine | Sertraline-d3 | ESI+ | 17.40 ± 0.05 | 0.96 ± 0.001 | IQL – 500 | 23.90 ± 4.31 | 0.999 | 0.003 | 0.001 | 94.9 | 9.5 |
|  | Norfluoxetine | Sertraline-d3 | ESI+ | 17.54 ± 0.17 | 0.97 ± 0.001 | IQL – 500 | 4.64 ± 0.39 | 1.000 | 0.003 | 0.01 | 86.8 | 6.2 |
|  | Gabapentin | Gabapentin-d4 | ESI+ | 7.99 ± 0.19 | 1.01 ± 0.003 | IQL – 1000 | 2.83 ± 0.25 | 0.999 | 0.03 | 0.07 | 99.5 | 4.1 |
|  | Gemfibrozil | Cortisol-d4 | ESI+ | 22.53 ± 0.11 | 1.36 ± 0.01 | IQL – 800 | 2.85 ± 0.56 | 0.998 | 0.09 | 0.3 | 50.8 | 4.3 |
|  | Gliclazide | Diazepam-d5 | ESI+ | 17.89 ± 0.01 | 0.95 ± 0.001 | IQL – 600 | 1.19 ± 0.06 | 1.000 | 0.002 | 0.001 | 95.0 | 10.3 |
|  | OH-Gliclazide | Metoprolol-d7 | ESI+ | 14.61 ± 0.02 | 1.30 ± 0.01 | IQL – 500 | 1.07 ± 0.13 | 0.994 | 0.01 | 0.002 | 71.6 | 1.6 |
|  | Ibuprofen^‡^ | Ibuprofen-d3 | ESI- | 10.50 ± 0.02 | 1.00 ± 0.001 | IQL – 3000 | - | 1.000 | 0.02 | 0.1 | 107.3 | 3.2 |
|  | 2-OH ibuprofen^‡^ | Estrone-d4 | ESI- | 7.80 ± 0.06 | 0.79 ± 0.01 | IQL – 750 | - | 0.998 | 0.1 | 0.2 | 100.9 | 3.0 |
|  | Ifosfamide | Carbamazepine 13C6 | ESI+ | 13.17 ± 0.09 | 0.82 ± 0.01 | IQL – 2000 | 1.96 ± 0.05 | 1.000 | 0.001 | 0.002 | 104.2 | 6.3 |
|  | Imatinib | Amitriptyline-d3 | ESI+ | 13.97 ± 0.09 | 0.77 ± 0.19 | IQL – 2000 | 8.04 ± 0.44 | 0.999 | 0.1 | 0.3 | 120.3 | 34.2 |
|  | Irbesartan | Bezafibrate-d6 | ESI- | 8.84 ± 0.02 | 1.07 ± 0.001 | IQL – 500 | 13.66 ± 1.94 | 0.998 | 0.08 | 0.2 | 78.7 | 4.7 |
|  | Ketoprofen | Bezafibrate-d6 | ESI- | 8.12 ± 0.02 | 0.99 ± 0.002 | IQL – 500 | - | 0.999 | 0.1 | 0.4 | 94.0 | 6.4 |
|  | Dihydroketoprofen | Bezafibrate-d6 | ESI- | 7.87 ± 0.03 | 0.96 ± 0.003 | IQL – 800 | 53.75 ± 22.59 | 0.998 | 0.1 | 0.4 | 87.4 | 5.9 |
|  | Lansoprazole | Desmethyl Diazepam-d5 | ESI+ | 16.38 ± 0.13 | 0.87 ± 0.15 | IQL – 2000 | 2.74 ± 0.74 | 0.993 | 0.02 | 0.05 | 101.9 | 13.1 |
|  | 5-OH Lansoprazole | Propranolol-d7 | ESI+ | 13.64 ± 0.03 | 0.94 ± 0.001 | IQL – 600 | 4.99 ± 0.24 | 0.997 | 0.1 | 0.2 | 105.0 | 6.9 |
|  | Lansoprazole Sulfone | Diazepam-d5 | ESI+ | 16.59 ± 0.15 | 0.88 ± 0.003 | IQL – 500 | 9.75 ± 0.42 | 0.998 | 0.003 | 0.01 | 89.5 | 6.5 |
|  | Levetiracetam | Ketamine-d4 | ESI+ | 6.05 ± 0.02 | 0.59 ± 0.004 | IQL – 2000 | 1.44 ± 0.03 | 1.000 | 0.04 | 0.1 | 100.2 | 15.3 |
|  | Lisinopril | Quetiapine-d8 | ESI+ | 9.63 ± 0.10 | 0.85 ± 0.004 | IQL – 1000 | 9.68 ± 0.36 | 0.991 | 0.01 | 0.02 | 88.2 | 4.1 |
|  | Methadone | Methadone-d9 | ESI+ | 16.68 ± 0.04 | 1.00 ± 0.001 | IQL – 500 | 1.15 ± 0.02 | 1.000 | 0.01 | 0.003 | 107.7 | 2.9 |
|  | EDDP | EDDP-d3 | ESI+ | 14.40 ± 0.05 | 1.00 ± 0.001 | IQL – 500 | 2.20 ± 0.05 | 1.000 | 0.01 | 0.003 | 101.0 | 4.4 |
|  | Memantine | Amphetamine-d5 | ESI+ | 15.03 ± 0.09 | 1.83 ± 0.03 | IQL – 1000 | 1.62 ± 0.16 | 0.999 | 0.002 | 0.01 | 89.3 | 7.2 |
|  | Metformin | Methamphetamine-d5 | ESI+ | 2.42 ± 0.05 | 0.28 ± 0.002 | IQL – 800 | 1.79 ± 0.10 | 0.992 | 0.1 | 0.4 | 103.8 | 8.9 |
|  | Guanyl Urea | Amphetamine-d5 | ESI+ | 2.61 ± 0.08 | 0.77 ± 0.02 | IQL – 750 | 2.58 ± 0.36 | 0.997 | 0.01 | 0.03 | 124.3 | 13.6 |
|  | Methotrexate | Quetiapine-d8 | ESI+ | 9.31 ± 0.07 | 0.61 ± 0.01 | IQL – 1000 | 1.45 ± 0.10 | 0.998 | 0.03 | 0.08 | 94.4 | 3.9 |
|  | Methyl prednisolone | Carbamazepine 13C6 | ESI+ | 17.92 ± 0.16 | 1.11 ± 0.002 | IQL – 500 | 3.87 ± 0.55 | 0.999 | 0.03 | 0.08 | 72.8 | 5.9 |
|  | Metoprolol | Metoprolol-d7 | ESI+ | 11.33 ± 0.08 | 1.00 ± 0.001 | IQL – 1000 | 2.06 ± 0.06 | 1.000 | 0.0003 | 0.001 | 109.4 | 2.2 |
|  | O-desmethyl metoprolol | Metoprolol-d7 | ESI+ | 7.70 ± 0.15 | 0.68 ± 0.01 | IQL – 500 | 1.40 ± 0.05 | 0.999 | 0.01 | 0.03 | 109.5 | 6.8 |
|  | Mirtazapine | Mirtazapine-d3 | ESI+ | 11.32 ± 0.05 | 1.00 ± 0.001 | IQL – 600 | 1.04 ± 0.02 | 0.998 | 0.01 | 0.002 | 94.2 | 4.2 |
|  | Morphine | Morphine-d3 | ESI+ | 3.08 ± 0.14 | 1.00 ± 0.05 | IQL – 500 | 11.04 ± 2.25 | 0.998 | 0.06 | 0.2 | 99.4 | 15.2 |
|  | Dihydromorphine | Cotinine-d3 | ESI+ | 3.22 ± 0.12 | 1.00 ± 0.03 | IQL – 1000 | 2.21 ± 0.17 | 0.999 | 0.08 | 0.2 | 86.1 | 7.6 |
|  | Normorphine | Morphine-d3 | ESI+ | 3.27 ± 0.12 | 1.02 ± 0.04 | IQL – 500 | 1.14 ± 0.23 | 0.999 | 0.08 | 0.2 | 92.2 | 9.4 |
|  | Naproxen | Naproxen-d3 | ESI- | 8.92 ± 0.02 | 1.00 ± 0.001 | IQL – 1500 | 1.17 ± 0.04 | 1.000 | 0.2 | 0.5 | 112.5 | 5.5 |
|  | O-desmethyl naproxen | Estrone-d4 | ESI- | 7.56 ± 0.01 | 0.76 ± 0.002 | IQL – 750 | 1.99 ± 0.08 | 0.999 | 1.5 | 4.7 | 95.5 | 3.6 |
|  | Nicorandil | Ketamine-d4 | ESI+ | 7.10 ± 0.14 | 0.69 ± 0.014 | IQL – 1000 | 1.15 ± 0.07 | 1.000 | 0.1 | 0.3 | 94.6 | 7.6 |
|  | Nitrazepam | Methadone-d9 | ESI+ | 16.42 ± 0.15 | 0.98 ± 0.002 | IQL – 500 | 2.55 ± 0.13 | 0.998 | 0.01 | 0.02 | 87.0 | 4.8 |
|  | Norephedrine | Caffeine-d9 | ESI+ | 5.38 ± 0.08 | 0.59 ± 0.005 | IQL – 500 | 2.78 ± 0.21 | 0.998 | 0.002 | 0.007 | 91.1 | 17.4 |
|  | Nortriptyline | Nortriptyline-d3 | ESI+ | 17.32 ± 0.05 | 1.00 ± 0.001 | IQL – 1000 | 1.26 ± 0.06 | 1.000 | 0.01 | 0.003 | 80.8 | 4.3 |
|  | 10-OH nortriptyline | Citalopram-d6 | ESI+ | 14.74 ± 0.07 | 1.02 ± 0.001 | IQL – 500 | 2.73 ± 0.39 | 0.999 | 0.002 | 0.001 | 97.6 | 7.3 |
|  | Oseltamivir | Desmethyl Diazepam-d5 | ESI+ | 14.97 ± 0.15 | 0.81 ± 0.003 | IQL – 500 | 1.03 ± 0.18 | 0.999 | 0.001 | 0.004 | 88.6 | 16.2 |
|  | Oxazepam | Desmethyl Diazepam-d5 | ESI+ | 17.57 ± 0.16 | 0.96 ± 0.002 | IQL – 500 | 1.27 ± 0.17 | 0.999 | 0.01 | 0.03 | 104.0 | 4.2 |
|  | Prednisolone | Cortisol-d4 | ESI+ | 16.56 ± 0.16 | 1.00 ± 0.001 | IQL – 500 | 31.15 ± 9.11 | 1.000 | 0.01 | 0.03 | 88.8 | 8.6 |
|  | Pregabalin | Gabapentin-d4 | ESI+ | 7.82 ± 0.19 | 0.99 ± 0.004 | IQL – 2000 | 17.80 ± 3.32 | 1.000 | 0.1 | 0.3 | 124.5 | 6.9 |
|  | N-methyl pregabalin | Gabapentin-d4 | ESI+ | 8.10 ± 0.16 | 1.00 ± 0.003 | IQL – 500 | 1.13 ± 0.03 | 0.999 | 0.04 | 0.1 | 91.5 | 4.5 |
|  | Propranolol | Propranolol-d7 | ESI+ | 14.57 ± 0.08 | 1.01 ± 0.002 | IQL – 500 | 2.11 ± 0.06 | 0.999 | 0.002 | 0.001 | 93.1 | 7.9 |
|  | Quetiapine | Quetiapine-d8 | ESI+ | 15.14 ± 0.06 | 1.00 ± 0.11 | IQL – 500 | 2.03 ± 0.03 | 1.000 | 0.002 | 0.001 | 90.9 | 2.2 |
|  | 7-OH Quetiapine | Quetiapine-d8 | ESI+ | 9.55 ± 0.09 | 0.63 ± 0.009 | IQL – 600 | 3.22 ± 0.14 | 1.000 | 0.002 | 0.01 | 86.0 | 5.6 |
|  | Ranitidine | Cotinine-d3 | ESI+ | 3.92 ± 0.09 | 0.81 ± 0.02 | IQL – 1000 | 18.47 ± 41.68 | 0.998 | 0.05 | 0.1 | 89.4 | 5.8 |
|  | Ranitidine N-oxide | Cocaethylene-d3 | ESI+ | 4.39 ± 0.07 | 0.34 ± 0.004 | IQL – 1000 | 1.33 ± 0.08 | 0.998 | 0.01 | 0.04 | 85.8 | 3.3 |
|  | Risperidone | Methadone-d9 | ESI+ | 12.79 ± 0.06 | 0.77 ± 0.01 | IQL – 500 | 7.11 ± 0.25 | 0.999 | 0.002 | 0.01 | 129.7 | 7.8 |
|  | Salbutamol | Norketamine-d4 | ESI+ | 3.93 ± 0.06 | 0.38 ± 0.004 | IQL – 600 | 3.57 ± 0.11 | 0.998 | 0.03 | 0.08 | 92.1 | 8.8 |
|  | Sertraline | Sertraline-d3 | ESI+ | 18.11 ± 0.03 | 1.00 ± 0.001 | IQL – 800 | 1.12 ± 0.077 | 1.000 | 0.001 | 0.0003 | 92.7 | 10.9 |
|  | Norsertraline | Sertraline-d3 | ESI+ | 18.56 ± 0.20 | 1.02 ± 0.01 | IQL – 500 | 7.65 ± 13.19 | 0.998 | 0.2 | 0.7 | 124.3 | 11.1 |
|  | Sildenafil | Quetiapine-d8 | ESI+ | 15.64 ± 0.17 | 1.03 ± 0.003 | IQL – 750 | 10.43 ± 0.44 | 1.000 | 0.002 | 0.01 | 78.7 | 4.6 |
|  | N-desmethyl sildenafil | Quetiapine-d8 | ESI+ | 15.72 ± 0.17 | 1.04 ± 0.003 | IQL – 1000 | 1.80 ± 0.09 | 0.998 | 0.01 | 0.02 | 87.5 | 5.1 |
|  | Simvastatin | Carbamazepine 13C6 | ESI+ | 23.31 ± 0.10 | 1.45 ± 0.01 | IQL – 500 | 1.42 ± 0.07 | 0.998 | 0.002 | 0.01 | 134.9 | 8.2 |
|  | Sitagliptin | Quetiapine-d8 | ESI+ | 11.73 ± 0.06 | 0.78 ± 0.002 | IQL – 2000 | 1.31 ± 0.05 | 0.999 | 0.01 | 0.03 | 93.6 | 2.8 |
|  | Tamoxifen | Verapamil-d7 | ESI+ | 20.56 ± 0.11 | 1.34 ± 0.01 | IQL – 200 | 24.21 ± 1.54 | 0.998 | 0.004 | 0.01 | 82.9 | 15.7 |
|  | Temazepam | Carbamazepine 13C6 | ESI+ | 17.89 ± 0.16 | 1.11 ± 0.002 | IQL – 500 | 2.25 ± 0.10 | 0.997 | 0.002 | 0.01 | 81.4 | 5.0 |
|  | Topiramate | Naproxen-d3 | ESI- | 7.55 ± 0.02 | 0.89 ± 0.004 | IQL – 750 | 11.10 ± 1.30 | 0.998 | 0.03 | 0.09 | 84.9 | 11.9 |
|  | Tramadol | Ketamine-d4 | ESI+ | 10.90 ± 0.05 | 1.05 ± 0.003 | IQL – 1500 | 13.70 ± 3.51 | 0.998 | 0.001 | 0.000 | 97.9 | 5.5 |
|  | O-desmethyl tramadol | Benzoylecgonine-d8 | ESI+ | 8.59 ± 0.11 | 0.80 ± 0.01 | IQL – 500 | 135.20 ± 21.90 | 0.998 | 0.002 | 0.01 | 93.9 | 1.6 |
|  | N-desmethyl tramadol | Ketamine-d4 | ESI+ | 11.85 ± 0.06 | 1.14 ± 0.004 | IQL – 2000 | 37.10 ± 5.75 | 0.999 | 0.002 | 0.001 | 98.4 | 5.1 |
|  | Valsartan | Bezafibrate-d6 | ESI- | 8.49 ± 0.01 | 1.00 ± 0.001 | IQL – 500 | 1.09 ± 0.13 | 0.999 | 0.3 | 0.9 | 115.8 | 1.4 |
|  | 4-hydroxy valsartan | Acetaminophen-d4 | ESI- | 7.12 ± 0.03 | 1.42 ± 0.01 | IQL – 500 | 2.49 ± 1.04 | 0.997 | 0.1 | 0.4 | 96.3 | 13.6 |
|  | Venlafaxine | Mirtazapine-d3 | ESI+ | 13.88 ± 0.12 | 1.22 ± 0.01 | IQL – 800 | 1.83 ± 0.16 | 0.999 | 0.1 | 0.4 | 87.5 | 5.4 |
|  | Desvenlafaxine | Mirtazapine-d3 | ESI+ | 10.97 ± 0.07 | 0.96 ± 0.01 | IQL – 500 | 10.02 ± 0.99 | 0.997 | 0.05 | 0.2 | 87.9 | 8.1 |
|  | Verapamil | Verapamil-d7 | ESI+ | 15.42 ± 0.16 | 1.00 ± 0.001 | IQL – 750 | 5.12 ± 0.13 | 1.000 | 0.0003 | 0.001 | 105.7 | 3.1 |
|  | p-O-desmethyl verapamil | Verapamil-d7 | ESI+ | 14.69 ± 0.15 | 0.96 ± 0.002 | IQL – 750 | 4.85 ± 0.16 | 0.999 | 0.002 | 0.01 | 86.7 | 4.4 |
|  | Zolpidem | Cocaine-d3 | ESI+ | 11.91 ± 0.06 | 1.04 ± 0.002 | IQL – 500 | 4.26 ± 0.21 | 0.999 | 0.001 | 0.004 | 106.9 | 10.4 |
| Human markers | 1,4‑methylimidazoleacetic acid | Cotinine-d3 | ESI+ | 2.49 ± 0.05 | 0.77 ± 0.01 | IQL – 2000 | 22.97 ± 1.92 | 0.998 | 0.02 | 0.07 | 95.7 | 14.4 |
|  | 2’-deoxyguanosine^*^ | Adenosine 13C5 | ESI+ | 3.42 ± 0.06 | 1.02 ± 0.03 | IQL – 200 | 3.96 ± 1.22 | 0.999 | 0.01 | 0.04 | 119.8 | 12.1 |
|  | 2'-deoxyinosine | Codeine-d6 | ESI+ | 3.38 ± 0.05 | 0.7 ± 0.01 | IQL – 1500 | 11.37 ± 3.67 | 0.997 | 0.02 | 0.1 | 79.1 | 6.1 |
|  | 3-nitro-L-tyrosine | Gabapentin-d4 | ESI+ | 4.91 ± 0.08 | 0.61 ± 0.01 | IQL – 500 | 4.91 ± 0.51 | 0.999 | 0.03 | 0.1 | 82.7 | 7.7 |
|  | 3-chloro-L-tyrosine | Gabapentin-d4 | ESI+ | 4.31 ± 0.09 | 0.53 ± 0.01 | IQL – 500 | 2.31 ± 0.15 | 0.999 | 0.09 | 0.3 | 93.5 | 10.7 |
|  | 5-Hydroxymethyl-2'-deoxyuridine | Carbamazepine 13C6 | ESI+ | 3.16 ± 0.09 | 0.20 ± 0.01 | IQL – 200 | 1.38 ± 0.34 | 0.998 | 0.05 | 0.1 | 84.2 | 11.2 |
|  | 5-hydroxyindole acetic acid | Acetaminophen-d4 | ESI+ | 8.27 ± 0.14 | 1.64 ± 0.02 | IQL – 250 | 18.02 ± 19.79 | 0.992 | 0.08 | 0.3 | 94.4 | 11.4 |
|  | 5-methyl-2'-deoxycytidine | Codeine-d6 | ESI+ | 2.98 ± 0.06 | 0.62 ± 0.01 | IQL – 750 | 18.68 ± 3.71 | 0.997 | 0.09 | 0.3 | 94.4 | 7.6 |
|  | 8-hydroxyguanosine | Adenosine 13C5 | ESI+ | 3.35 ± 0.07 | 0.99 ± 0.01 | IQL – 500 | - | 0.997 | 0.03 | 0.08 | 93.0 | 6.3 |
|  | 8-oxoguanine | Acetaminophen-d4 | ESI+ | 3.37 ± 0.08 | 0.67 ± 0.01 | IQL – 1500 | 0.47 ± 0.05 | 0.998 | 0.1 | 0.3 | 95.0 | 8.6 |
|  | Adenosine | Adenosine 13C5 | ESI+ | 3.37 ± 0.04 | 1.00 ± 0.002 | IQL – 1000 | 9.99 ± 0.91 | 0.999 | 0.03 | 0.08 | 68.5 | 9.9 |
|  | Androstenedione | Cocaethylene-d3 | ESI+ | 18.61 ± 0.03 | 1.46 ± 0.004 | IQL – 500 | 1.51 ± 0.10 | 1.000 | 0.003 | 0.001 | 102.7 | 10.2 |
|  | Asymmetric dimethyl arginine | Cotinine-d3 | ESI+ | 2.40 ± 0.07 | 0.74 ± 0.02 | IQL – 500 | 1.43 ± 0.13 | 0.997 | 0.01 | 0.02 | 124.9 | 8.3 |
|  | Cortisol | Cortisol-d4 | ESI+ | 16.55 ± 0.16 | 1.00 ± 0.001 | IQL – 500 | 3.42 ± 0.20 | 1.000 | 0.01 | 0.02 | 92.9 | 5.2 |
|  | Cortisone | Cortisol-d4 | ESI+ | 15.81 ± 0.03 | 0.96 ± 0.001 | IQL – 600 | 2.41 ± 0.28 | 0.991 | 0.004 | 0.01 | 95.1 | 2.6 |
|  | Creatinine | Codeine-d6 | ESI+ | 2.47 ± 0.04 | 0.51 ± 0.01 | IQL – 2000 | 3.76 ± 0.19 | 0.999 | 0.002 | 0.01 | 97.0 | 13.6 |
|  | Deoxyadenosine | Cotinine-d3 | ESI+ | 3.39 ± 0.04 | 0.89 ± 0.01 | IQL – 500 | 10.81 ± 1.41 | 0.993 | 0.2 | 0.6 | 80.1 | 8.4 |
|  | Dihydrobiopterin | Cotinine-d3 | ESI+ | 3.08 ± 0.06 | 0.95 ± 0.01 | IQL – 250 | 1.46 ± 0.21 | 0.999 | 0.04 | 0.1 | 115.0 | 14.1 |
|  | Dihydrotestosterone | Cortisol-d4 | ESI+ | 20.55 ± 0.12 | 1.24 ± 0.01 | IQL – 750 | 3.19 ± 0.38 | 0.998 | 0.01 | 0.02 | 79.6 | 3.7 |
|  | Estradiol | Estradiol-d4 | ESI- | 9.63 ± 0.02 | 1.00 ± 0.002 | IQL – 750 | 1.23 ± 0.19 | 0.999 | 0.3 | 1.1 | 92.5 | 4.3 |
|  | Estrone | Estrone-d4 | ESI- | 9.70 ± 0.02 | 1.00 ± 0.002 | IQL – 1000 | - | 1.000 | 0.06 | 0.2 | 98.9 | 3.3 |
|  | Formiminoglutamic acid | Cotinine-d3 | ESI+ | 2.73 ± 0.09 | 0.84 ± 0.02 | IQL – 1500 | 0.72 ± 0.06 | 0.994 | 0.04 | 0.1 | 117.3 | 8.2 |
|  | Hippuric acid | Gabapentin-d4 | ESI+ | 8.81 ± 0.02 | 1.12 ± 0.03 | IQL – 750 | 2.12 ± 0.32 | 0.999 | 0.01 | 0.04 | 87.4 | 7.0 |
|  | 4-Hydroxy-2-nonenal mercapturic acid | Bezafibrate-d6 | ESI- | 7.50 ± 0.01 | 0.88 ± 0.001 | IQL – 800 | 1.29 ± 0.08 | 0.997 | 0.1 | 0.4 | 106.4 | 2.7 |
|  | Hydroxymethyl uracil | Cotinine-d3 | ESI+ | 3.06 ± 0.11 | 0.91 ± 0.03 | IQL – 200 | 1.80 ± 0.26 | 0.999 | 0.2 | 0.6 | 84.9 | 19.4 |
|  | Indoxyl sulfate | Acetaminophen-d4 | ESI- | 6.10 ± 0.03 | 1.18 ± 0.02 | IQL – 1000 | 3.93 ± 0.41 | 0.999 | 0.7 | 2.0 | 91.1 | 4.1 |
|  | Inosine | Codeine-d6 | ESI+ | 3.22 ± 0.06 | 0.67 ± 0.01 | IQL – 1000 | 14.10 ± 3.22 | 0.997 | 0.02 | 0.06 | 77.6 | 4.1 |
|  | Nε-(1-Carboxyethyl)-L-lysine | Cotinine-d3 | ESI+ | 2.61 ± 0.06 | 0.81 ± 0.02 | IQL – 600 | 1.70 ± 0.11 | 0.997 | 0.1 | 0.4 | 100.2 | 11.2 |
|  | Nε-(1-Carboxymethyl)-L-lysine | Cotinine-d3 | ESI+ | 2.56 ± 0.06 | 0.79 ± 0.01 | IQL – 600 | 1.77 ± 0.06 | 0.998 | 0.01 | 0.03 | 124.6 | 11.5 |
|  | Neopterin | Cotinine-d3 | ESI+ | 2.86 ± 0.07 | 0.88 ± 0.02 | IQL – 200 | 0.64 ± 0.07 | 0.998 | 0.1 | 0.3 | 47.3 | 9.1 |
|  | Phenyl acetyl glutamine | Caffeine-d9 | ESI+ | 9.07 ± 0.08 | 1.01 ± 0.002 | IQL – 1500 | 1.48 ± 0.06 | 0.995 | 0.03 | 0.08 | 62.1 | 4.2 |
|  | Pterin | Cotinine-d3 | ESI+ | 3.25 ± 0.05 | 1.01 ± 0.01 | IQL – 500 | 1.90 ± 0.22 | 0.998 | 0.04 | 0.1 | 99.2 | 7.9 |
|  | Progesterone | Cocaine-d3 | ESI+ | 21.10 ± 0.02 | 1.86 ± 0.01 | IQL – 2000 | 1.20 ± 0.06 | 0.999 | 0.01 | 0.002 | 100.0 | 4.6 |
|  | Pyroglutamic acid | Cotinine-d3 | ESI+ | 2.66 ± 0.20 | 0.70 ± 0.05 | IQL – 2000 | 8.59 ± 1.45 | 0.998 | 0.1 | 0.3 | 102.6 | 8.9 |
|  | Testosterone | Cortisol-d4 | ESI+ | 19.34 ± 0.03 | 1.18 ± 0.001 | IQL – 600 | 1.24 ± 0.08 | 1.000 | 0.01 | 0.002 | 94.3 | 6.5 |
| Food | 1-methyl-2-pyridone-5-carboxamide | Cotinine-d3 | ESI+ | 3.40 ± 0.05 | 1.05 ± 0.01 | IQL – 600 | 7.5 ± 0.58 | 0.998 | 0.04 | 0.1 | 70.5 | 4.3 |
|  | 1-methylhistidine | Amphetamine-d5 | ESI+ | 2.40 ± 0.06 | 0.28 ± 0.02 | IQL – 1000 | 1.73 ± 0.09 | 0.997 | 0.01 | 0.01 | 98.1 | 8.0 |
|  | 3-carboxy-4-methyl-5-propyl-2-furanpropanoic acid | Carbamazepine 13C6 | ESI+ | 18.83 ± 0.15 | 1.17 ± 0.003 | IQL – 500 | 2.98 ± 0.19 | 0.998 | 0.09 | 0.3 | 67.2 | 25.0 |
|  | 3-methylhistidine | Amphetamine-d5 | ESI+ | 2.43 ± 0.04 | 0.29 ± 0.01 | IQL – 1000 | 4.80 ± 0.24 | 0.997 | 0.01 | 0.03 | 107.5 | 6.9 |
|  | 4-Pyridoxic acid | Cotinine-d3 | ESI+ | 3.53 ± 0.03 | 1.09 ± 0.002 | IQL – 1000 | 0.90 ± 0.04 | 0.996 | 0.2 | 0.7 | 100.5 | 7.1 |
|  | 5-(3′,4′-Dihydroxyphenyl)-γ-valerolactone | Caffeine-d9 | ESI+ | 9.59 ± 0.08 | 1.06 ± 0.002 | IQL – 1000 | 1.60 ± 0.07 | 0.994 | 0.07 | 0.2 | 98.8 | 6.4 |
|  | Acesulfame K | Bezafibrate-d6 | ESI- | 3.41 ± 0.05 | 0.40 ± 0.06 | IQL – 750 | 24.78 ± 5.90 | 0.998 | 0.1 | 0.3 | 101.8 | 7.6 |
|  | a-CEHC | Acetaminophen-d4 | ESI- | 8.76 ± 0.02 | 1.69 ± 0.02 | IQL – 800 | 32.82 ± 6.15 | 0.999 | 0.1 | 0.20 | 103.0 | 5.2 |
|  | Aspartame | Cocaine-d3 | ESI+ | 11.09 ± 0.07 | 0.97 ± 0.002 | IQL – 500 | 1.91 ± 0.21 | 0.997 | 0.01 | 0.02 | 96.0 | 7.7 |
|  | Carnitine | Cotinine-d3 | ESI+ | 2.38 ± 0.04 | 0.63 ± 0.01 | IQL – 1000 | 1.07 ± 0.05 | 0.998 | 0.2 | 0.7 | 105.4 | 10.0 |
|  | Daidzein | Methylparaben 13C6 | ESI- | 7.90 ± 0.02 | 1.04 ± 0.002 | IQL – 500 | 1.07 ± 0.07 | 0.995 | 0.3 | 0.8 | 111.9 | 7.2 |
|  | Enterodiol | Naproxen-d3 | ESI- | 7.92 ± 0.02 | 0.89 ± 0.003 | IQL – 500 | 2.06 ± 0.08 | 0.999 | 0.4 | 1.3 | 118.1 | 6.8 |
|  | Enterolactone | Methylparaben 13C6 | ESI- | 8.32 ± 0.33 | 1.07 ± 0.04 | IQL – 750 | 1.01 ± 0.10 | 1.000 | 0.1 | 0.3 | 117.2 | 2.0 |
|  | Epicatechin | Estrone-d4 | ESI- | 6.56 ± 0.07 | 0.80 ± 0.01 | IQL – 800 | 1.31 ± 0.38 | 0.998 | 0.4 | 1.2 | 65.9 | 15.1 |
|  | Equol | Methylparaben 13C6 | ESI- | 8.16 ± 0.02 | 1.07 ± 0.002 | IQL – 500 | 1.36 ± 0.05 | 0.999 | 0.2 | 0.7 | 81.3 | 14.0 |
|  | Ferulic Acid | Ibuprofen-d3 | ESI- | 6.58 ± 0.02 | 0.63 ± 0.001 | IQL – 500 | 53.80 ± 10.00 | 0.999 | 0.1 | 0.4 | 108.5 | 5.0 |
|  | Genistein | Bezafibrate-d6 | ESI- | 8.39 ± 0.02 | 1.02 ± 0.002 | IQL – 2000 | 3.85 ± 0.47 | 0.997 | 0.3 | 0.9 | 105.5 | 5.7 |
|  | Glycitein | Methylparaben 13C6 | ESI- | 7.93 ± 0.02 | 1.04 ± 0.002 | IQL – 1000 | 3.34 ± 0.10 | 1.000 | 0.3 | 1.0 | 86.3 | 6.1 |
|  | Histidine | Codeine-d6 | ESI+ | 2.43 ± 0.04 | 0.51 ± 0.01 | IQL – 600 | 7.76 ± 0.59 | 0.995 | 0.04 | 0.1 | 98.1 | 14.3 |
|  | Lysine | Methamphetamine-d5 | ESI+ | 2.44 ± 0.04 | 0.28 ± 0.004 | IQL – 500 | 1.73 ± 0.18 | 0.999 | 0.3 | 0.8 | 110.2 | 10.8 |
|  | Methionine | Metoprolol-d7 | ESI+ | 2.95 ± 0.06 | 0.26 ± 0.01 | IQL – 1500 | 2.26 ± 0.17 | 0.999 | 0.1 | 0.36 | 83.0 | 16.9 |
|  | N-methylnicotinamide | Cotinine-d3 | ESI+ | 3.63 ± 0.04 | 0.99 ± 0.004 | IQL – 1000 | 1.17 ± 0.04 | 0.999 | 0.01 | 0.02 | 96.3 | 8.1 |
|  | Pantothenic acid | Acetaminophen-d4 | ESI- | 3.32 ± 0.03 | 0.66 ± 0.01 | IQL – 500 | 1.99 ± 0.11 | 0.999 | 0.09 | 0.3 | 117.5 | 12.0 |
|  | Phenylalanine | Gabapentin-d4 | ESI+ | 4.66 ± 0.09 | 0.58 ± 0.01 | IQL – 1000 | 4.87 ± 0.61 | 0.998 | 0.2 | 0.5 | 91.5 | 6.0 |
|  | Phloretin | Naproxen-d3 | ESI- | 8.23 ± 0.02 | 0.97 ± 0.01 | IQL – 500 | 5.47 ± 0.50 | 0.999 | 1.2 | 3.6 | 90.3 | 6.5 |
|  | Resveratrol | Estradiol-d4 | ESI- | 7.31 ± 0.13 | 0.76 ± 0.01 | IQL – 1000 | 3.24 ± 0.58 | 0.999 | 1.6 | 5.0 | 77.6 | 14.7 |
|  | Riboflavin | Caffeine-d9 | ESI+ | 10.47 ± 0.07 | 1.16 ± 0.004 | IQL – 1000 | 2.19 ± 0.10 | 0.993 | 0.02 | 0.05 | 85.0 | 3.1 |
|  | Saccharin | Bezafibrate-d6 | ESI- | 4.42 ± 0.10 | 0.52 ± 0.01 | IQL – 500 | 1.06 ± 0.03 | 0.999 | 1.6 | 5.0 | 100.6 | 4.1 |
|  | Stachydrine | Cotinine-d3 | ESI+ | 2.87 ± 0.07 | 0.89 ± 0.02 | IQL – 1000 | 2.45 ± 0.23 | 0.999 | 0.03 | 0.1 | 107.6 | 8.6 |
|  | Sucralose | Naproxen-d3 | ESI- | 6.80 ± 0.03 | 0.77 ± 0.01 | IQL – 5000 | 1.79 ± 0.32 | 0.999 | 2.4 | 7.3 | 146.1 | 12.6 |
|  | D,L-Sulforaphane N-acetyl L-cysteine | Diazepam-d5 | ESI+ | 9.16 ± 0.02 | 0.49 ± 0.001 | IQL – 1000 | 1.06 ± 0.02 | 0.998 | 0.01 | 0.003 | 97.6 | 6.9 |
|  | Trimethylamine N-oxide | Atenolol-d7 | ESI+ | 2.61 ± 0.07 | 0.69 ± 0.02 | IQL – 500 | 5.03 ± 1.79 | 0.999 | 0.02 | 0.06 | 105.5 | 30.9 |
|  | Tryptophan | Gabapentin-d4 | ESI+ | 7.07 ± 0.18 | 0.87 ± 0.01 | IQL – 1000 | 1.61 ± 0.16 | 0.997 | 0.01 | 0.02 | 120.8 | 25.6 |
|  | Urolithin A | Methylparaben 13C6 | ESI- | 7.95 ± 0.02 | 1.04 ± 0.004 | IQL – 2000 | 3.12 ± 0.88 | 1.000 | 0.7 | 2.0 | 88.1 | 12.1 |
|  | Valine | Amphetamine-d5 | ESI+ | 2.75 ± 0.04 | 0.31 ± 0.003 | IQL – 2000 | 7.92 ± 0.78 | 0.999 | 0.05 | 0.1 | 97.0 | 6.1 |
| Personal care products | 3-hydroxypropyl mercapturic acid | Codeine-d6 | ESI+ | 4.26 ± 0.10 | 0.88 ± 0.02 | IQL – 750 | 1.38 ± 0.26 | 0.998 | 0.06 | 0.2 | 107.9 | 9.3 |
|  | Benzophenone-1 | Methylparaben 13C6 | ESI- | 9.38 ± 0.02 | 1.23 ± 0.003 | IQL – 2000 | 1.15 ± 0.02 | 1.000 | 0.09 | 0.3 | 93.9 | 6.7 |
|  | Benzophenone-2 | Estrone-d4 | ESI- | 7.87 ± 0.02 | 0.81 ± 0.003 | IQL – 250 | 1.21 ± 0.05 | 0.998 | 1.2 | 3.7 | 93.3 | 7.0 |
|  | Benzophenone-4 | Naproxen-d3 | ESI- | 7.31 ± 0.08 | 0.82 ± 0.002 | IQL – 500 | 1.08 ± 0.04 | 0.999 | 0.7 | 0.2 | 102.7 | 3.3 |
|  | Bisphenol A | Ibuprofen-d3 | ESI- | 8.99 ± 0.03 | 0.9 ± 0.003 | IQL – 1000 | 2.36 ± 0.49 | 0.999 | 0.1 | 0.2 | 88.6 | 9.3 |
|  | Chloroxylenol | Bisphenol A-d16 | ESI- | 10.23 ± 0.02 | 1.12 ± 0.003 | IQL – 500 | 4.42 ± 0.50 | 0.997 | 0.6 | 0.2 | 89.8 | 3.6 |
|  | Butylparaben | Methylparaben 13C6 | ESI- | 9.81 ± 0.02 | 1.29 ± 0.004 | IQL – 1000 | 2.84 ± 0.06 | 1.000 | 0.01 | 0.02 | 97.3 | 8.3 |
|  | Ethylparaben | Methylparaben 13C6 | ESI- | 8.26 ± 0.02 | 1.08 ± 0.002 | IQL – 500 | 3.74 ± 0.27 | 0.999 | 0.05 | 0.1 | 91.7 | 7.0 |
|  | Methylparaben | Methylparaben 13C6 | ESI- | 7.78 ± 0.01 | 1.00 ± 0.001 | IQL – 2000 | 3.08 ± 0.09 | 1.000 | 0.09 | 0.03 | 108.5 | 5.7 |
|  | Propylparaben | Methylparaben 13C6 | ESI- | 9.02 ± 0.02 | 1.18 ± 0.003 | IQL – 500 | 3.56 ± 0.13 | 1.000 | 0.03 | 0.1 | 94.7 | 6.4 |

**Table S8.** Minimum, median, weekly average and maximum daily load for all analytes, reported in g/day

| Class | Compound | Minimum (g/day) | Median (g/day) | Average (g/day) | Maximum (g/day) |
| --- | --- | --- | --- | --- | --- |
|  |  |  |  |  |  |
| Illicit drugs | 4-methylmethcathinone | <LOD | <LOD | <LOD | <LOD |
|  | Amphetamine | 0.69 ± 0.20 | 1.7 ± 0.15 | 1.71 ± 0.81 | 3.22 ± 0.77 |
|  | Cocaine | 5.22 ± 0.03 | 13.41 ± 0.15 | 12.22 ± 4.49 | 18.95 ± 0.24 |
|  | Anhydroecgonine methylester | <LOD | <LOD | <LOD | <LOD |
|  | Benzoylecgonine | 9.81 ± 0.23 | 24.78 ± 0.72 | 23.45 ± 10.17 | 39.1 ± 0.83 |
|  | Cocaethylene | 0.17 ± 0.01 | 0.3 ± 0.03 | 0.38 ± 0.22 | 0.78 ± 0.14 |
|  | Heroin | <LOD | <LOD | <LOD | <LOD |
|  | 6-monoacetylmorphine | 0.07 ± 0.01 | 0.13 ± 0.01 | 0.13 ± 0.08 | 0.18 ± 0.01 |
|  | Ketamine | 10.99 ± 0.07 | 19.23 ± 0.28 | 19.62 ± 8.44 | 34.8 ± 0.86 |
|  | Norketamine | 0.6 ± 0.03 | 0.85 ± 0.06 | 0.89 ± 0.26 | 1.4 ± 0.05 |
|  | Methamphetamine | 0.05 ± 0.01 | 0.07 ± 0.01 | 0.09 ± 0.05 | 0.18 ± 0.03 |
|  | MDA | <LOD | <LOD | <LOD | <LOD |
|  | MDMA | 0.07 ± 0.01 | 0.28 ± 0.03 | 0.27 ± 0.24 | 0.73 ± 0 |
|  | MDPV | <LOD | <LOD | <LOD | <LOD |
| Lifestyle chemicals | Caffeine | 141.39 ± 2.42 | 337.84 ± 0.07 | 304.76 ± 108.29 | 431.75 ± 1.48 |
|  | 7-methylxanthine | 443.44 ± 38.92 | 949.85 ± 10.2 | 849.04 ± 249.02 | 1138.24 ± 177.21 |
|  | Paraxanthine | 66.14 ± 3.93 | 125.01 ± 0.19 | 121.81 ± 39.56 | 173.02 ± 35.78 |
|  | Nicotine | 2.67 ± 0.16 | 6.81 ± 1.79 | 6.83 ± 3.67 | 12.13 ± 4.96 |
|  | Cotinine | 4.76 ± 0.1 | 9.9 ± 0.01 | 9.29 ± 3.01 | 11.94 ± 0.05 |
| Pharmaceuticals | 17α-Ethinylestradiol (EE2) | <LOD | <LOD | <LOD | <LOD |
|  | Acetaminophen | 764.35 ± 10.39 | 1686.41 ± 41.22 | 1453.5 ± 505.31 | 2010.87 ± 8.96 |
|  | Amitriptyline | 0.55 ± 0.03 | 1.01 ± 0.08 | 1.03 ± 0.48 | 1.64 ± 0.09 |
|  | Amlodipine | <LOD | <LOD | <LOD | <LOD |
|  | O-des[2-aminoethyl]O-carboxymethyl dehydroamlodipine | 1.03 ± 0.05 | 2.08 ± 0 | 1.9 ± 0.46 | 2.4 ± 0.12 |
|  | Atenolol | 2.46 ± 0.07 | 6.2 ± 0.39 | 5.39 ± 1.91 | 7.22 ± 0.04 |
|  | Atorvastatin | 0.39 ± 0.03 | 0.97 ± 0.14 | 0.96 ± 0.53 | 1.68 ± 0.19 |
|  | 2-OH Atorvastatin | 1.35 ± 0.19 | 2.93 ± 0.55 | 3.02 ± 1.33 | 4.74 ± 0.41 |
|  | Beclomethasone | <LOD | <LOD | <LOD | <LOD |
|  | Betamethasone / Dexamethasone | <LOD | <LOD | <LOD | <LOD |
|  | Bendroflumethiazide | <LOD | <LOD | <LOD | <LOD |
|  | Bezafibrate | 0.75 ± 0.04 | 2.08 ± 0.25 | 1.82 ± 0.86 | 2.77 ± 0.45 |
|  | Bisoprolol | 0.19 ± 0.01 | 0.43 ± 0.05 | 0.46 ± 0.21 | 0.85 ± 0.22 |
|  | Budesonide | <LOD | <LOD | <LOD | <LOD |
|  | Buprenorphine | <LOD | <LOD | <LOD | <LOD |
|  | Capecitabine | <LOD | <LOD | <LOD | <LOD |
|  | Carbamazepine | 0.53 ± 0.01 | 0.71 ± 0.09 | 0.83 ± 0.32 | 1.48 ± 0.02 |
|  | Carbamazepine-10,11-epoxide | 0.88 ± 0.29 | 2.47 ± 0.17 | 2.44 ± 1.02 | 3.79 ± 0.03 |
|  | 10,11-dihydro-10-hydroxy carbamazepine | 0.02 ± 0.02 | 0.47 ± 0.35 | 0.35 ± 0.27 | 0.72 ± 0.01 |
|  | Cimetidine | 0.61 ± 0.06 | 0.66 ± 0.03 | 0.66 ± 0.04 | 0.72 ± 0.02 |
|  | Citalopram | 1.84 ± 0.04 | 3.73 ± 0.03 | 3.33 ± 0.96 | 4.29 ± 0.06 |
|  | Desmethyl citalopram | 0.69 ± 0.15 | 1.73 ± 0.21 | 1.5 ± 0.53 | 1.94 ± 0.41 |
|  | Clobazam | <LOD | <LOD | <LOD | <LOD |
|  | Codeine | 4.89 ± 0.45 | 12.92 ± 0.65 | 11.99 ± 4.92 | 17.41 ± 0.07 |
|  | Norcodeine | <LOD | <LOD | <LOD | <LOD |
|  | Dihydrocodeine | 0.69 ± 0.08 | 1.21 ± 0.02 | 1.15 ± 0.31 | 1.46 ± 0.06 |
|  | Diazepam | <LOD | <LOD | <LOD | <LOD |
|  | Desmethyl diazepam | <LOD | <LOD | <LOD | <LOD |
|  | Diclofenac | 0.44 ± 0.11 | 1.92 ± 0.31 | 1.74 ± 0.87 | 2.57 ± 0.02 |
|  | 4-hydroxy diclofenac | <LOD | <LOD | <LOD | <LOD |
|  | Diltiazem | 1.66 ± 0.06 | 2.53 ± 0.29 | 2.52 ± 0.66 | 3.53 ± 0.15 |
|  | N-desmethyl diltiazem | <LOD | <LOD | <LOD | <LOD |
|  | Donepezil | <LOD | <LOD | <LOD | <LOD |
|  | Duloxetine | <LOD | <LOD | <LOD | <LOD |
|  | Ephedrine | 0.72 ± 0.02 | 1.74 ± 0.09 | 1.62 ± 0.74 | 2.81 ± 0.01 |
|  | Fexofenadine | 4.43 ± 0.45 | 8.77 ± 0.47 | 8.67 ± 3.27 | 14.19 ± 10.39 |
|  | Fluoxetine | <LOD | 0.36 ± 0.05 | 0.36 ± 0.04 | 0.4 ± 0.04 |
|  | Norfluoxetine | <LOD | <LOD | <LOD | <LOD |
|  | Gabapentin | 41.26 ± 0.16 | 108.69 ± 1.27 | 90.4 ± 31.63 | 122.08 ± 0.31 |
|  | Gemfibrozil | <LOD | <LOD | <LOD | <LOD |
|  | Gliclazide | 0 ± 0 | 0.46 ± 0.13 | 0.46 ± 0.51 | 0.82 ± 0.09 |
|  | OH-Gliclazide | 0.79 ± 0.11 | 1.15 ± 0.11 | 1.31 ± 0.48 | 2.04 ± 0.33 |
|  | Ibuprofen^‡^ | 36.95 ± 0.09 | 86.28 ± 1.23 | 80.62 ± 31.2 | 115.74 ± 3.54 |
|  | 2-OH ibuprofen^‡^ | 81.49 ± 11.7 | 215.22 ± 1.46 | 203.03 ± 93.38 | 307.89 ± 202.77 |
|  | Ifosfamide | <LOD | <LOD | <LOD | <LOD |
|  | Imatinib | <LOD | <LOD | <LOD | <LOD |
|  | Irbesartan | 0.79 ± 0.03 | 0.91 ± 0.05 | 0.93 ± 0.11 | 1.11 ± 0.12 |
|  | Ketoprofen | 1.1 ± 0.01 | 1.62 ± 1.02 | 2 ± 1.08 | 3.74 ± 0.4 |
|  | Dihydroketoprofen | <LOD | <LOD | <LOD | <LOD |
|  | Lansoprazole | <LOD | <LOD | <LOD | <LOD |
|  | 5-OH Lansoprazole | <LOD | <LOD | <LOD | <LOD |
|  | Lansoprazole Sulfone | <LOD | <LOD | <LOD | <LOD |
|  | Levetiracetam | 26.04 ± 2.16 | 69.3 ± 35.23 | 63.03 ± 21.98 | 85.41 ± 8.07 |
|  | Lisinopril | <LOD | <LOD | <LOD | <LOD |
|  | Methadone | <LOD | 0.05 ± 0.03 | 0.06 ± 0.05 | 0.14 ± 0.16 |
|  | EDDP | 0.26 ± 0.2 | 0.46 ± 0.11 | 0.47 ± 0.17 | 0.71 ± 0.01 |
|  | Memantine | 0.4 ± 0.01 | 0.43 ± 0.02 | 0.44 ± 0.03 | 0.5 ± 0.03 |
|  | Metformin | 22.36 ± 0.68 | 39.2 ± 10.46 | 42.72 ± 18.47 | 70.38 ± 12.4 |
|  | Guanyl Urea | 60.71 ± 9.35 | 68.26 ± 0.88 | 76.97 ± 17.23 | 102.37 ± 26.67 |
|  | Methotrexate | 0.34 ± 0 | 0.39 ± 0 | 0.4 ± 0.04 | 0.45 ± 0.02 |
|  | Methyl prednisolone | <LOD | <LOD | <LOD | <LOD |
|  | Metoprolol | 0.07 ± 0.01 | 0.09 ± 0.01 | 0.10 ± 0.03 | 0.15 ± 0.02 |
|  | O-desmethyl metoprolol | <LOD | <LOD | <LOD | <LOD |
|  | Mirtazapine | 0.39 ± 0.01 | 0.65 ± 0.02 | 0.7 ± 0.26 | 1 .00 ± 0.01 |
|  | N-desmethyl mirtazapine | 0.41 ± 0.01 | 0.47 ± 0.02 | 0.48 ± 0.06 | 0.57 ± 0.03 |
|  | Morphine | 0.61 ± 0.2 | 2.47 ± 0.03 | 2.1 ± 1.08 | 3.33 ± 0.39 |
|  | Dihydromorphine | <LOD | <LOD | <LOD | <LOD |
|  | Normorphine | 0.79 ± 0.08 | 1.37 ± 0.17 | 1.36 ± 0.49 | 2.21 ± 0.75 |
|  | Naproxen | 24.23 ± 0.53 | 55.44 ± 1.06 | 50.21 ± 16.95 | 68.79 ± 0.37 |
|  | O-desmethyl naproxen | 9.83 ± 1.29 | 17.42 ± 0.12 | 18.75 ± 7.1 | 29.46 ± 19.19 |
|  | Nicorandil | <LOD | <LOD | <LOD | <LOD |
|  | Nitrazepam | <LOD | <LOD | <LOD | <LOD |
|  | Norephedrine | <LOD | <LOD | <LOD | <LOD |
|  | Nortriptyline | <LOD | <LOD | <LOD | <LOD |
|  | 10-OH nortriptyline | <LOD | <LOD | <LOD | <LOD |
|  | Oseltamivir | <LOD | <LOD | <LOD | <LOD |
|  | Oxazepam | <LOD | <LOD | <LOD | <LOD |
|  | Prednisolone | <LOD | <LOD | <LOD | <LOD |
|  | Pregabalin | 10.85 ± 0.26 | 22.41 ± 0.04 | 20.58 ± 6.99 | 29.21 ± 0.12 |
|  | N-methyl pregabalin | 0.2 ± 0.05 | 0.31 ± 0.21 | 0.33 ± 0.09 | 0.46 ± 0.02 |
|  | Propranolol | 0.97 ± 0.03 | 1.47 ± 0.01 | 1.69 ± 0.94 | 3.71 ± 1.16 |
|  | Quetiapine | <LOD | 0.03 ± 0.08 | 0.04 ± 0.05 | 0.11 ± 0.05 |
|  | 7-OH Quetiapine | 0.22 ± 0.05 | 1.16 ± 0.05 | 1.12 ± 0.87 | 2.71 ± 0.12 |
|  | Ranitidine | <LOD | <LOD | <LOD | <LOD |
|  | Ranitidine N-oxide | <LOD | <LOD | <LOD | <LOD |
|  | Risperidone | <LOD | <LOD | <LOD | <LOD |
|  | Salbutamol | 0 ± 0 | 0.04 ± 0.01 | 0.04 ± 0.02 | 0.07 ± 0.01 |
|  | Sertraline | 1.91 ± 0.06 | 3.04 ± 0.87 | 4.28 ± 3.07 | 10.73 ± 2.43 |
|  | Norsertraline | <LOD | <LOD | <LOD | <LOD |
|  | Sildenafil | <LOD | <LOD | <LOD | <LOD |
|  | N-desmethyl sildenafil | <LOD | <LOD | <LOD | <LOD |
|  | Simvastatin | <LOD | <LOD | <LOD | <LOD |
|  | Sitagliptin | <LOD | <LOD | <LOD | <LOD |
|  | Tamoxifen | <LOD | <LOD | <LOD | <LOD |
|  | Temazepam | <LOD | <LOD | <LOD | <LOD |
|  | Topiramate | 0.17 ± 0.04 | 0.37 ± 0.16 | 0.4 ± 0.18 | 0.64 ± 0.09 |
|  | Tramadol | 3.63 ± 0.06 | 9.47 ± 1.09 | 9.1 ± 4.61 | 16.65 ± 5.3 |
|  | O-desmethyl tramadol | 2.04 ± 0.03 | 6.26 ± 0.6 | 7.01 ± 4.15 | 13.83 ± 2.5 |
|  | N-desmethyl tramadol | 0.22 ± 0.02 | 0.41 ± 0.09 | 0.49 ± 0.25 | 0.92 ± 0.34 |
|  | Valsartan | 1.42 ± 0.22 | 2.59 ± 0.32 | 2.66 ± 0.89 | 3.98 ± 1.01 |
|  | 4-hydroxy valsartan | <LOD | <LOD | <LOD | <LOD |
|  | Venlafaxine | 1.7 ± 0.06 | 7.08 ± 0.47 | 6.14 ± 3.04 | 9.9 ± 3.42 |
|  | Desvenlafaxine | 9.18 ± 0.43 | 9.93 ± 0.25 | 10.35 ± 1.17 | 12.58 ± 0.24 |
|  | Verapamil | <LOD | 0.04 ± 0 | 0.04 ± 0.05 | 0.06 ± 0.02 |
|  | p-O-desmethyl verapamil | <LOD | <LOD | <LOD | <LOD |
|  | Zolpidem | <LOD | <LOD | <LOD | <LOD |
| Human markers | 1,4‑methylimidazoleacetic acid | 70.88 ± 3.22 | 156.54 ± 3.46 | 144.32 ± 48.35 | 192.74 ± 8.36 |
|  | 2’-deoxyguanosine^*^ | <LOD | <LOD | <LOD | <LOD |
|  | 2'-deoxyinosine | 3.37 ± 0.23 | 7.97 ± 0.38 | 13.42 ± 12.56 | 34.72 ± 0.93 |
|  | 3-nitro-L-tyrosine | <LOD | <LOD | <LOD | <LOD |
|  | 3-chloro-L-tyrosine | 3.58 ± 0.07 | 5.15 ± 0.21 | 5.51 ± 1.72 | 8.55 ± 0.19 |
|  | 5-Hydroxymethyl-2'-deoxyuridine | <LOD | <LOD | <LOD | <LOD |
|  | 5-hydroxyindole acetic acid | <LOD | <LOD | <LOD | <LOD |
|  | 5-methyl-2'-deoxycytidine | <LOD | <LOD | <LOD | <LOD |
|  | 8-hydroxyguanosine | 1.88 ± 0.02 | 2.46 ± 0.11 | 2.43 ± 0.35 | 2.84 ± 0.13 |
|  | 8-oxoguanine | <LOD | <LOD | <LOD | <LOD |
|  | Adenosine | 140.11 ± 5.8 | 412.8 ± 13.87 | 391.01 ± 185.57 | 591.8 ± 11.87 |
|  | Androstenedione | 1 ± 0.04 | 1.09 ± 0.02 | 1.15 ± 0.2 | 1.57 ± 0.03 |
|  | Asymmetric dimethyl arginine | <LOD | <LOD | <LOD | <LOD |
|  | Cortisol | 0 ± 0 | 0.9 ± 0.32 | 0.78 ± 0.43 | 0.99 ± 0.75 |
|  | Cortisone | 1.54 ± 0.05 | 2 ± 0.27 | 2.2 ± 0.64 | 3.28 ± 0.64 |
|  | Creatinine | 3615.29 ± 152.32 | 6158.14 ± 157.63 | 5836.47 ± 1697.14 | 7658.76 ± 227.42 |
|  | Deoxyadenosine | 0.93 ± 0 | 1.55 ± 0.11 | 8.06 ± 10.83 | 26.41 ± 0.02 |
|  | Dihydrobiopterin | <LOD | <LOD | <LOD | <LOD |
|  | Dihydrotestosterone | <LOD | <LOD | <LOD | <LOD |
|  | Estradiol (E2) | <LOD | <LOD | <LOD | <LOD |
|  | Estrone (E1) | 0.25 ± 0.01 | 0.4 ± 0.11 | 0.48 ± 0.18 | 0.78 ± 0.35 |
|  | Formiminoglutamic acid | <LOD | <LOD | <LOD | <LOD |
|  | Hippuric acid | 68.13 ± 0.05 | 190.64 ± 55.81 | 244.55 ± 199.44 | 651.8 ± 135.19 |
|  | HNE-MA | 1.72 ± 0.09 | 3.87 ± 0.88 | 3.44 ± 1.11 | 4.73 ± 0.11 |
|  | Hydroxymethyl uracil | <LOD | <LOD | <LOD | <LOD |
|  | Indoxyl sulfate | 415.88 ± 27.97 | 907.87 ± 90 | 835.4 ± 329.27 | 1281.24 ± 357.69 |
|  | Inosine | <LOD | <LOD | <LOD | <LOD |
|  | Nε-(1-Carboxyethyl)-L-lysine | 56.28 ± 1.64 | 103.55 ± 5.18 | 103.33 ± 39.22 | 161.8 ± 20.38 |
|  | Nε-(1-Carboxymthyl)-L-lysine | 51.26 ± 1.55 | 92.82 ± 2.83 | 94.62 ± 38.93 | 158.02 ± 8.21 |
|  | Neopterin | <LOD | <LOD | <LOD | <LOD |
|  | Phenyl acetyl glutamine | 77.96 ± 2 | 137.66 ± 1.36 | 172.12 ± 91.58 | 287.5 ± 17.86 |
|  | Pterin | 4.87 ± 0.57 | 10.89 ± 1.06 | 9.8 ± 3.28 | 12.83 ± 0.08 |
|  | Progesterone | 1.03 ± 0.01 | 1.16 ± 0.08 | 1.2 ± 0.18 | 1.5 ± 0.2 |
|  | Pyroglutamic acid | 344.53 ± 2.41 | 657.46 ± 22.52 | 746.33 ± 341.04 | 1272.04 ± 17.9 |
|  | Testosterone | <LOD | <LOD | <LOD | <LOD |
| Food | 1-methyl-2-pyridone-5-carboxamide | 460 ± 4.69 | 866.42 ± 53.28 | 771.19 ± 211.12 | 1033.95 ± 188.97 |
|  | 1-methylhistidine | <LOD | <LOD | <LOD | <LOD |
|  | 3-carboxy-4-methyl-5-propyl-2-furanpropanoic acid (CMPF) | <LOD | <LOD | <LOD | <LOD |
|  | 3-methylhistidine | 744.77 ± 65.62 | 1718.13 ± 46.26 | 1614.05 ± 693.62 | 2457.9 ± 9.54 |
|  | 4-Pyridoxic acid | 59.56 ± 0.36 | 108.7 ± 10.42 | 101.73 ± 27.33 | 134.61 ± 13.49 |
|  | 5-(3′,4′-Dihydroxyphenyl)-γ-valerolactone | 0.58 ± 0.04 | 0.71 ± 0.09 | 0.74 ± 0.14 | 0.96 ± 0.03 |
|  | Acesulfame K | 50.77 ± 3.93 | 111.69 ± 0.51 | 111.14 ± 43.78 | 165.85 ± 5.04 |
|  | a-CEHC | 3.85 ± 1.07 | 9.48 ± 1.85 | 8.28 ± 3.45 | 12.76 ± 0.21 |
|  | Aspartame | 2.51 ± 0.17 | 5.4 ± 0.01 | 5.88 ± 3.4 | 12.99 ± 0.45 |
|  | Carnitine | 181.73 ± 3.81 | 376.12 ± 0.37 | 389.55 ± 167.25 | 629.79 ± 26.65 |
|  | Daidzein | 3.65 ± 1.42 | 7.58 ± 0.01 | 7.16 ± 2.37 | 10.45 ± 0.75 |
|  | Enterodiol | 3.03 ± 0.1 | 6.74 ± 0.42 | 6.02 ± 1.94 | 8.07 ± 0.52 |
|  | Enterolactone | 17.51 ± 0.14 | 48.96 ± 4.89 | 42.24 ± 12.93 | 52.96 ± 1.36 |
|  | Epicatechin | <LOD | <LOD | <LOD | <LOD |
|  | Equol | 6.41 ± 0.07 | 15.43 ± 0.04 | 13.82 ± 5.51 | 21.5 ± 0.52 |
|  | Ferulic Acid | 7.9 ± 1.81 | 23.97 ± 3.23 | 26.45 ± 14.27 | 45.43 ± 1.63 |
|  | Genistein | 2.81 ± 0.52 | 4.13 ± 0.9 | 4.59 ± 1.32 | 6.22 ± 3.19 |
|  | Glycitein | 0.09 ± 0.2 | 0.96 ± 0.22 | 0.81 ± 0.37 | 1.16 ± 0.09 |
|  | Histidine | 84.42 ± 7.66 | 204.79 ± 6.83 | 304.26 ± 248.27 | 754.98 ± 63.28 |
|  | Lysine | 428.98 ± 50.68 | 1713.36 ± 248.56 | 1713.02 ± 1040.01 | 3141.26 ± 775.9 |
|  | Methionine | 31.19 ± 0.17 | 682.53 ± 13.14 | 647.28 ± 582.38 | 1718.18 ± 86.67 |
|  | N-methylnicotinamide | <LOD | <LOD | <LOD | <LOD |
|  | Pantothenic acid | <LOD | <LOD | <LOD | <LOD |
|  | Phenylalanine | 42.59 ± 0.68 | 346.41 ± 10.4 | 388.55 ± 358.32 | 1082.89 ± 42 |
|  | Phloretin | <LOD | <LOD | <LOD | <LOD |
|  | Resveratrol | 5.8 ± 1.03 | 9.31 ± 2.64 | 9.05 ± 2.52 | 12.49 ± 5.72 |
|  | Riboflavin | 16.07 ± 0.29 | 28.71 ± 0.15 | 31.6 ± 14.95 | 60.52 ± 3.00 |
|  | Saccharin | 32.01 ± 3.18 | 76.32 ± 6.44 | 65.45 ± 26.35 | 99.11 ± 4.71 |
|  | Stachydrine | 465.44 ± 32.78 | 813.88 ± 23.98 | 822.22 ± 335.94 | 1488.36 ± 2.88 |
|  | Sucralose | 95.13 ± 7.79 | 130.28 ± 35.32 | 138.04 ± 29.61 | 182.05 ± 3.36 |
|  | D,L-Sulforaphane N-acetyl L-cysteine | 1.07 ± 0.08 | 1.66 ± 0.08 | 1.79 ± 0.6 | 2.65 ± 0.03 |
|  | Trimethylamine N-oxide | 806.61 ± 84.14 | 1312.62 ± 2.02 | 1207.36 ± 212.47 | 1394.70 ± 62.92 |
|  | Tryptophan | 24.01 ± 1.23 | 128.77 ± 4.61 | 137.76 ± 108.88 | 338.78 ± 7.46 |
|  | Urolithin A | 8.36 ± 1.76 | 16.72 ± 1.42 | 15.46 ± 4.93 | 23 ± 3.75 |
|  | Valine | 506.5 ± 73.89 | 2186.71 ± 599.44 | 2051.5 ± 1188.31 | 4176.8 ± 303.32 |
| Personal care products | 3-hydroxypropyl mercapturic acid | 13.79 ± 4.24 | 22.6 ± 4.34 | 23.18 ± 7.45 | 35.27 ± 9 |
|  | Benzophenone-1 | 0.5 ± 0.07 | 0.91 ± 0.04 | 0.79 ± 0.25 | 1.05 ± 0.07 |
|  | Benzophenone-2 | <LOD | <LOD | <LOD | <LOD |
|  | Benzophenone-4 | 9.88 ± 0.7 | 20.47 ± 1.2 | 17.3 ± 5.26 | 22.67 ± 0.68 |
|  | Bisphenol A | <LOD | <LOD | <LOD | <LOD |
|  | Chloroxylenol | 6.7 ± 2.21 | 29.94 ± 2.13 | 30.07 ± 20.76 | 67.68 ± 20.85 |
|  | Butylparaben | 0.1 ± 0.01 | 0.14 ± 0.04 | 0.14 ± 0.03 | 0.17 ± 0.01 |
|  | Ethylparaben | 0.2 ± 0.02 | 1.41 ± 0.23 | 1.26 ± 0.62 | 1.78 ± 0.59 |
|  | Methylparaben | 2.78 ± 0.22 | 5.97 ± 0.56 | 5.75 ± 1.95 | 8.14 ± 0.06 |
|  | Propylparaben | 1.21 ± 0.05 | 3.14 ± 0.24 | 3.01 ± 1.12 | 4.43 ± 0.08 |

**Figure S4.** 7-day temporal study on the ‘lifestyle chemical’ BCIs, quantified in influent wastewater. Figure A details the wastewater concentration, LOQ is determined via the MQL (Table 1). Figure B is the daily load, for the same samples. Here, daily load LOQ was calculated by multiplying the MQL by the average daily flow rate (L/day) across the 7-day sampling week. Abbreviations are defined within Table S1.


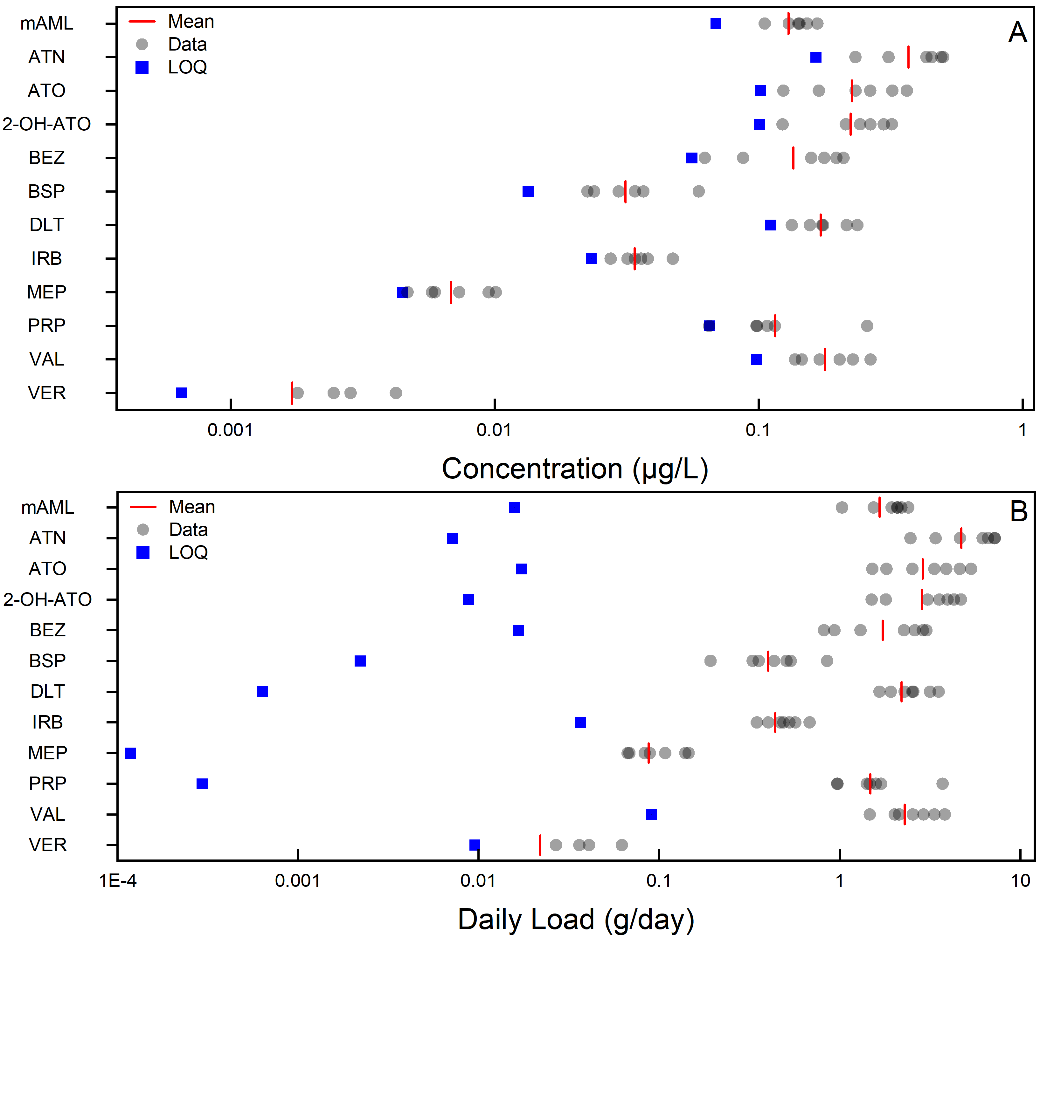


**Figure S5**. 7-day temporal study on the pharmaceutical sub-class ‘cardiovascular drug’ BCIs, quantified in influent wastewater. Figure A details the wastewater concentration, LOQ is determined via the MQL (Table 1). Figure B is the daily load, for the same samples. Here, daily load LOQ was calculated by multiplying the MQL by the average daily flow rate (L/day) across the 7-day sampling week. Abbreviations are defined within Table S1.


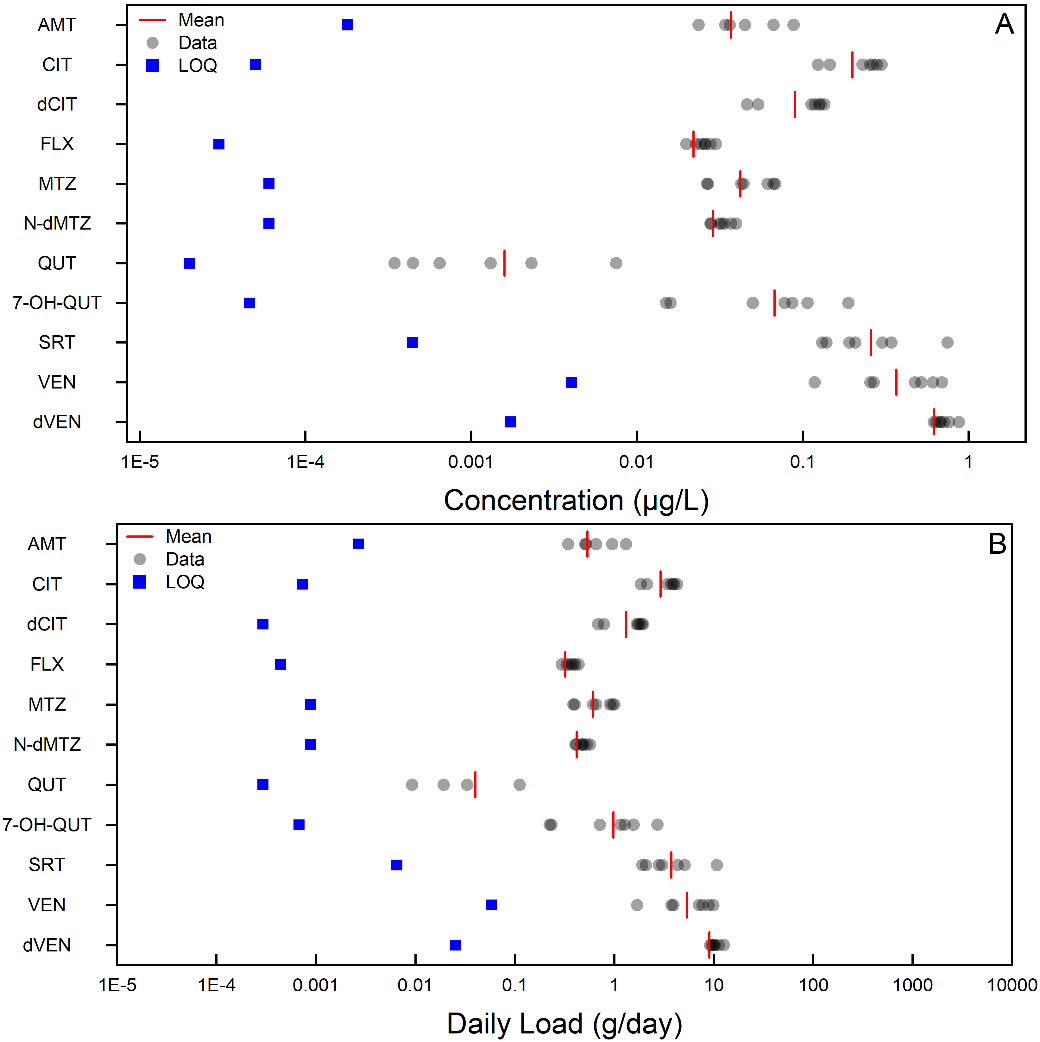


**Figure S6**. 7-day temporal study on the pharmaceutical sub-class ‘anxiety’ BCIs, quantified in influent wastewater. Figure A details the wastewater concentration, LOQ is determined via the MQL (Table 1). Figure B is the daily load, for the same samples. Here, daily load LOQ was calculated by multiplying the MQL by the average daily flow rate (L/day) across the 7-day sampling week. Abbreviations are defined within Table S1.


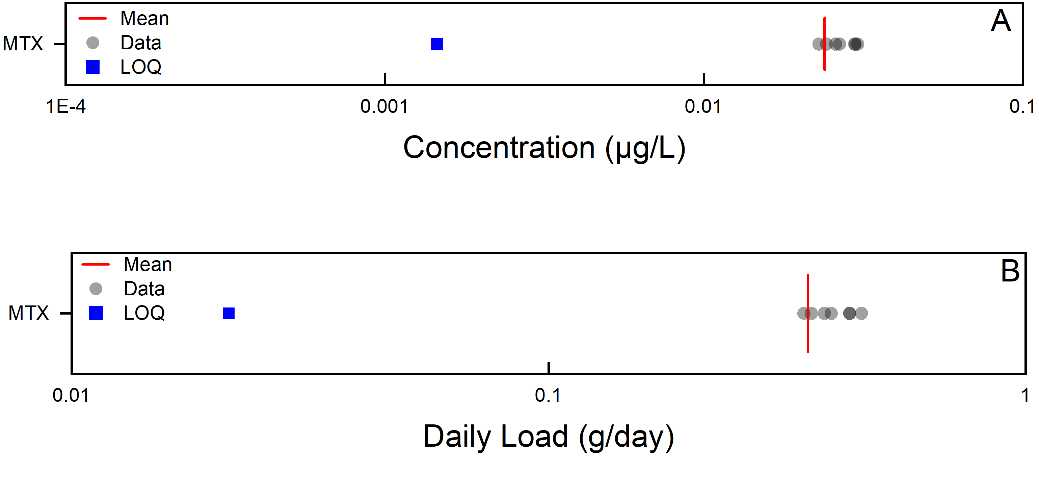


**Figure S7**. 7-day temporal study on the pharmaceutical sub-class ‘cancer’ BCIs, quantified in influent wastewater. Figure A details the wastewater concentration, LOQ is determined via the MQL (Table 1). Figure B is the daily load, for the same samples. Here, daily load LOQ was calculated by multiplying the MQL by the average daily flow rate (L/day) across the 7-day sampling week. Abbreviations are defined within Table S1.


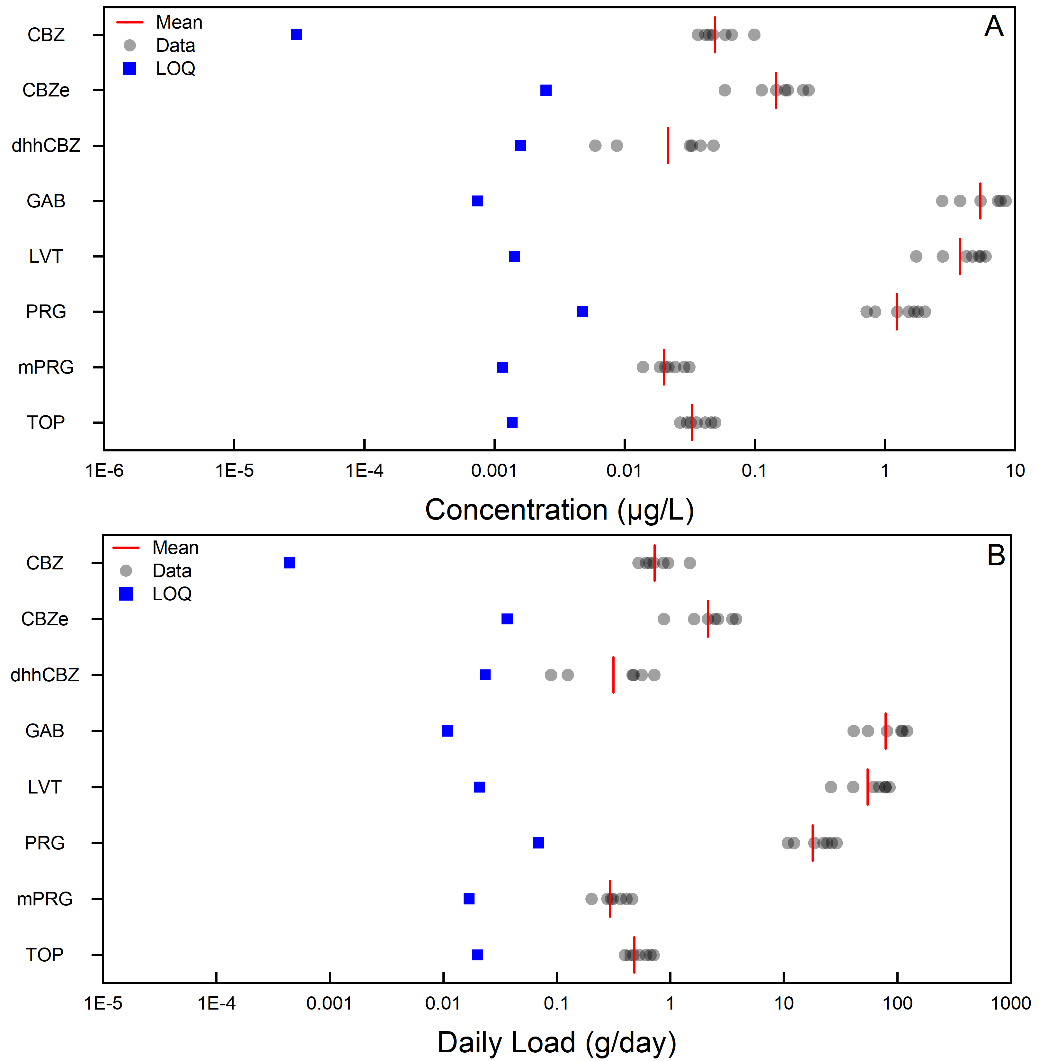


**Figure S8**. 7-day temporal study on the pharmaceutical sub-class ‘anti-epileptic’ BCIs, quantified in influent wastewater. Figure A details the wastewater concentration, LOQ is determined via the MQL (Table 1). Figure B is the daily load, for the same samples. Here, daily load LOQ was calculated by multiplying the MQL by the average daily flow rate (L/day) across the 7-day sampling week. Abbreviations are defined within Table S1.


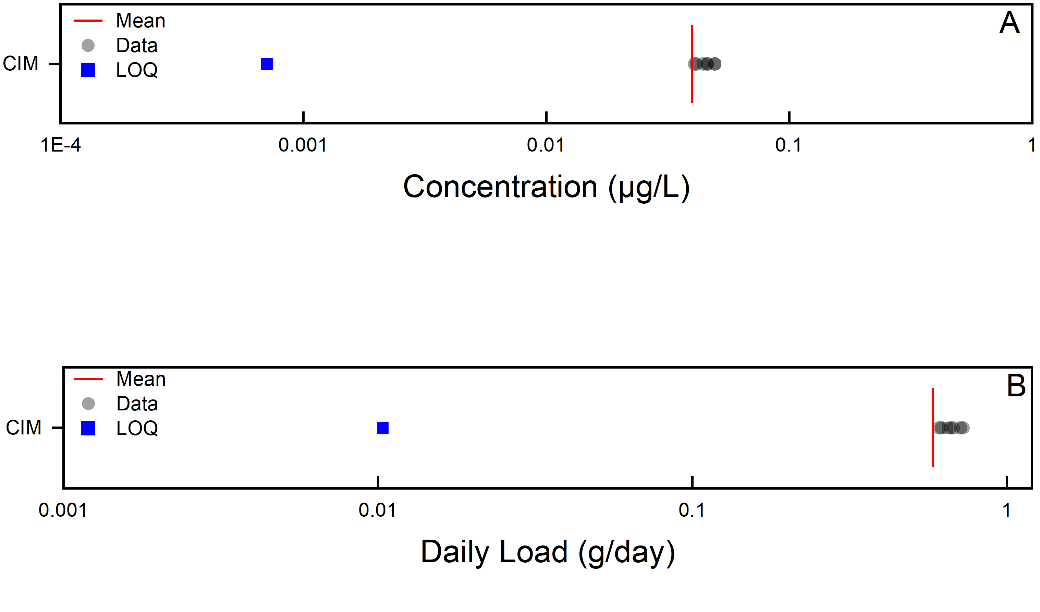


**Figure S9**. 7-day temporal study on the pharmaceutical sub-class ‘ulcer’ BCIs, quantified in influent wastewater. Figure A details the wastewater concentration, LOQ is determined via the MQL (Table 1). Figure B is the daily load, for the same samples. Here, daily load LOQ was calculated by multiplying the MQL by the average daily flow rate (L/day) across the 7-day sampling week. Abbreviations are defined within Table S1.


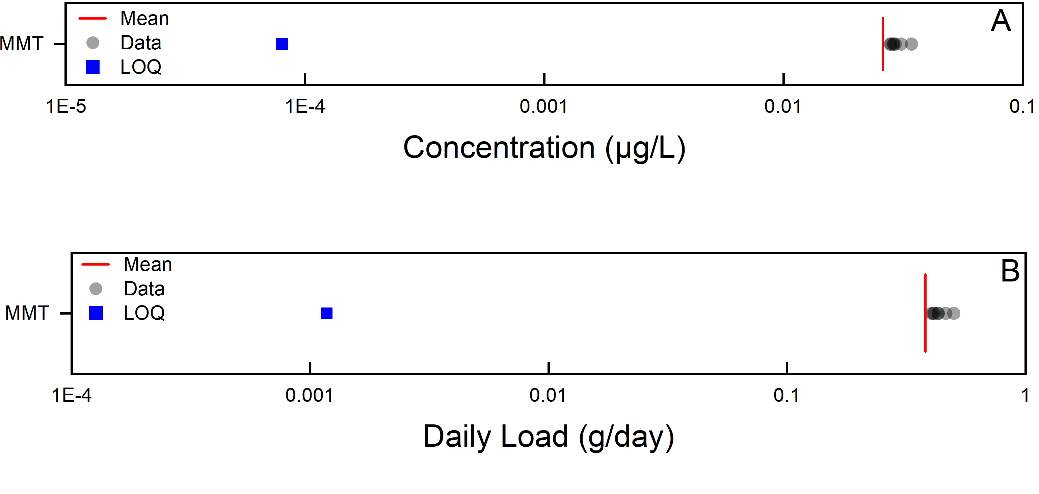


**Figure S10**. 7-day temporal study on the pharmaceutical sub-class ‘dementia’ BCIs, quantified in influent wastewater. Figure A details the wastewater concentration, LOQ is determined via the MQL (Table 1). Figure B is the daily load, for the same samples. Here, daily load LOQ was calculated by multiplying the MQL by the average daily flow rate (L/day) across the 7-day sampling week. Abbreviations are defined within Table S1.


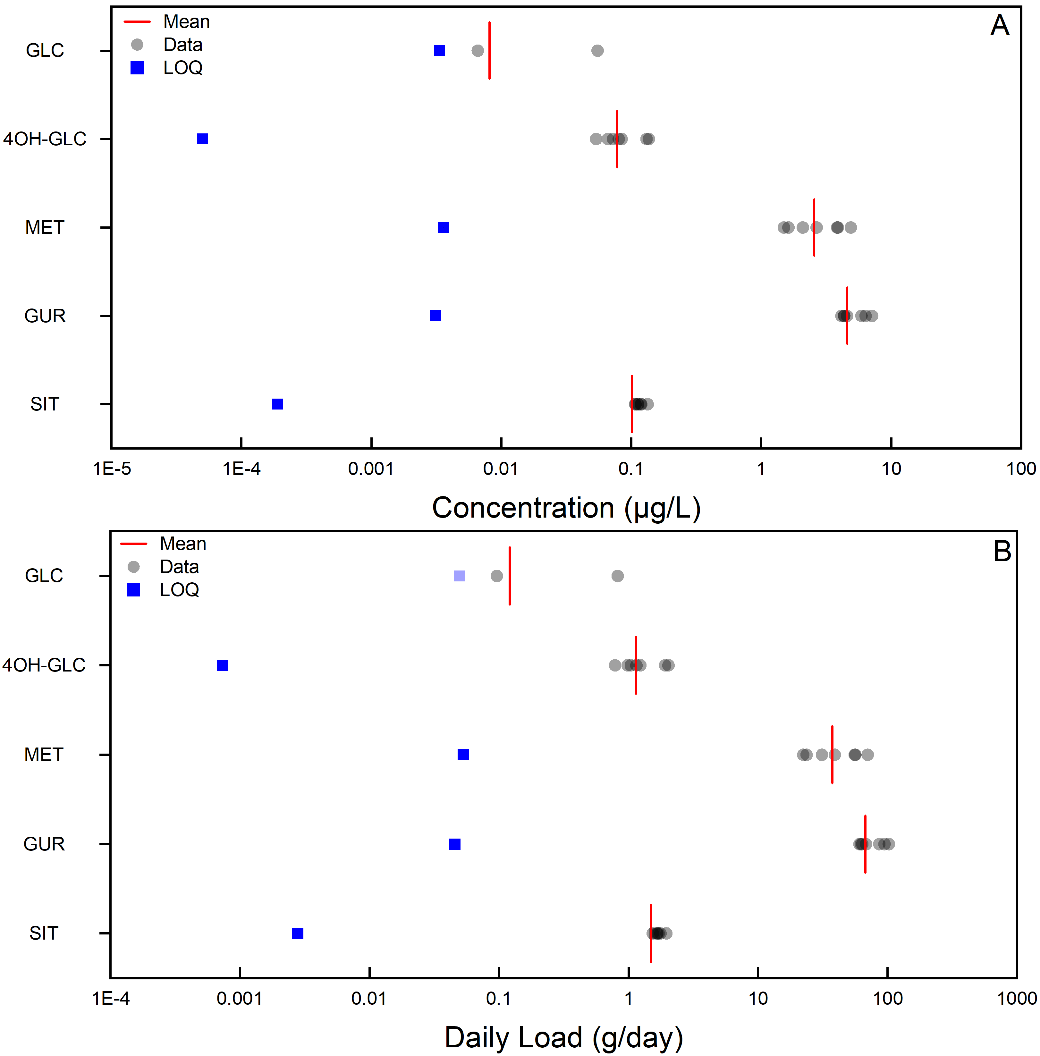


**Figure S11**. 7-day temporal study on the pharmaceutical sub-class ‘diabetes’ BCIs, quantified in influent wastewater. Figure A details the wastewater concentration, LOQ is determined via the MQL (Table 1). Figure B is the daily load, for the same samples. Here, daily load LOQ was calculated by multiplying the MQL by the average daily flow rate (L/day) across the 7-day sampling week. Abbreviations are defined within Table S1.


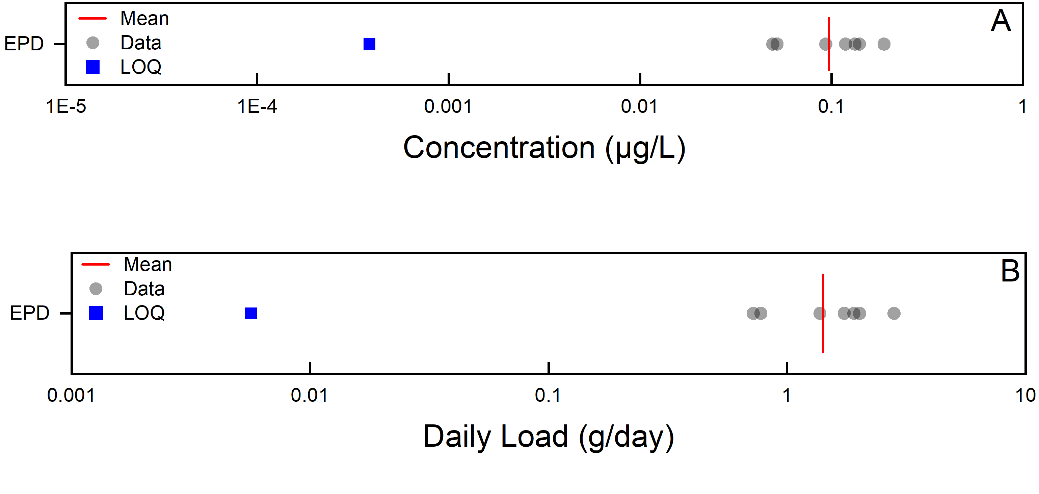


**Figure S12**. 7-day temporal study on the pharmaceutical sub-class ‘stimulant’ BCIs, quantified in influent wastewater. Figure A details the wastewater concentration, LOQ is determined via the MQL (Table 1). Figure B is the daily load, for the same samples. Here, daily load LOQ was calculated by multiplying the MQL by the average daily flow rate (L/day) across the 7-day sampling week. Abbreviations are defined within Table S1.


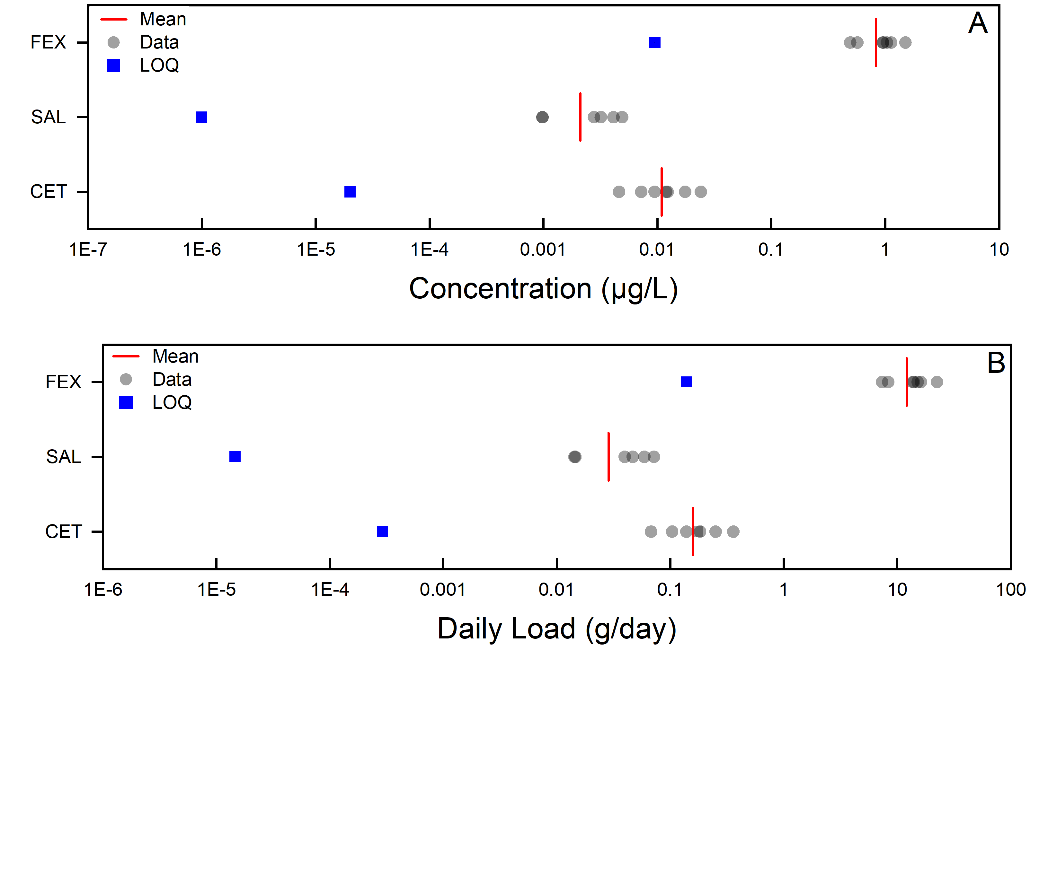


**Figure S13**. 7-day temporal study on the pharmaceutical sub-class ‘anti-histamine/allergy’ BCIs, quantified in influent wastewater. Figure A details the wastewater concentration, LOQ is determined via the MQL (Table 1). Figure B is the daily load, for the same samples. Here, daily load LOQ was calculated by multiplying the MQL by the average daily flow rate (L/day) across the 7-day sampling week. Abbreviations are defined within Table S1.


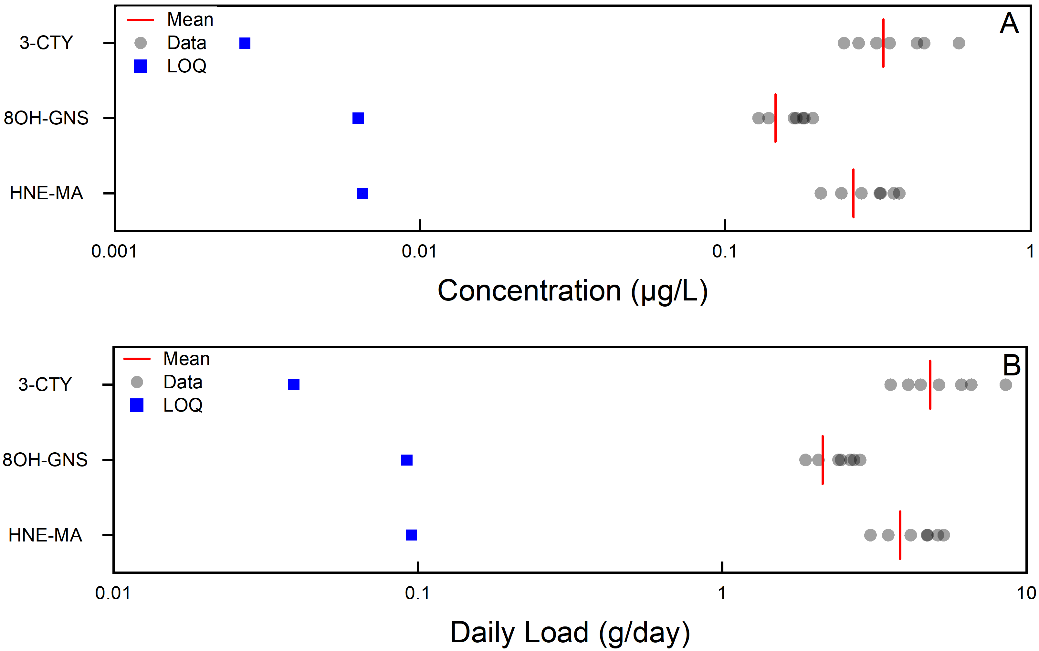


**Figure S14.** 7-day temporal study on the health markers sub-class ‘stress’ BCIs, quantified in influent wastewater. Figure A details the wastewater concentration, LOQ is determined via the MQL (Table 1). Figure B is the daily load, for the same samples. Here, daily load LOQ was calculated by multiplying the MQL by the average daily flow rate (L/day) across the 7-day sampling week. Abbreviations are defined within Table S1.


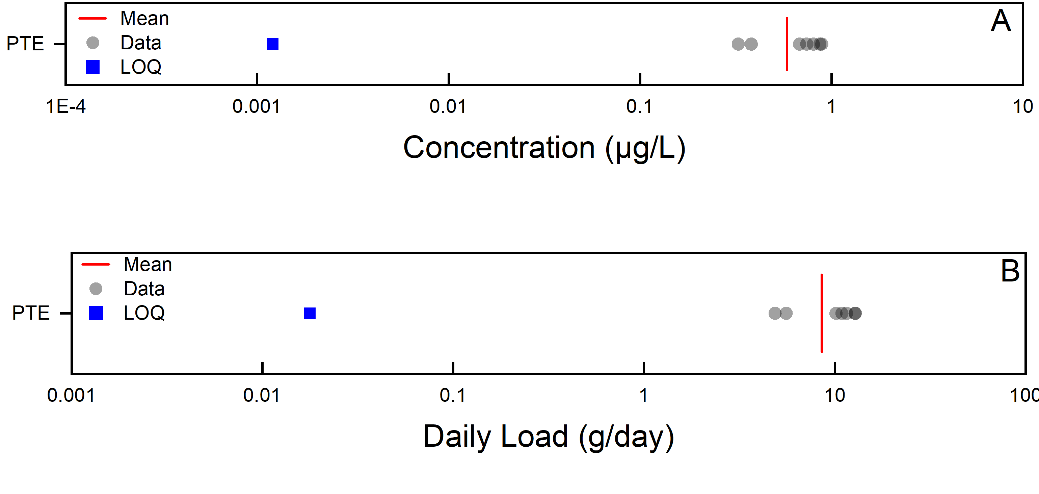


**Figure S15.** 7-day temporal study on the health markers sub-class ‘pteridine’ BCIs, quantified in influent wastewater. Figure A details the wastewater concentration, LOQ is determined via the MQL (Table 1). Figure B is the daily load, for the same samples. Here, daily load LOQ was calculated by multiplying the MQL by the average daily flow rate (L/day) across the 7-day sampling week. Abbreviations are defined within Table S1.


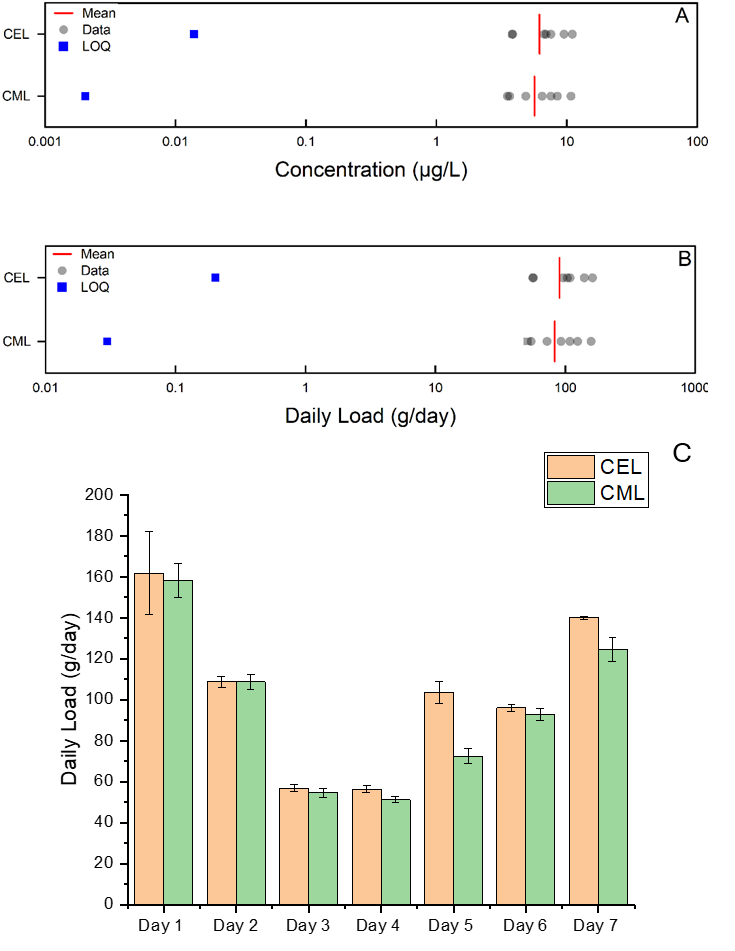


**Figure S16.** 7-day temporal study on the health markers sub-class ‘AGEs’ BCIs, quantified in influent wastewater. Figure A details the wastewater concentration, LOQ is determined via the MQL (Table 1). Figure B is the daily load, for the same samples. Here, daily load LOQ was calculated by multiplying the MQL by the average daily flow rate (L/day) across the 7-day sampling week. Abbreviations are defined within Table S1.


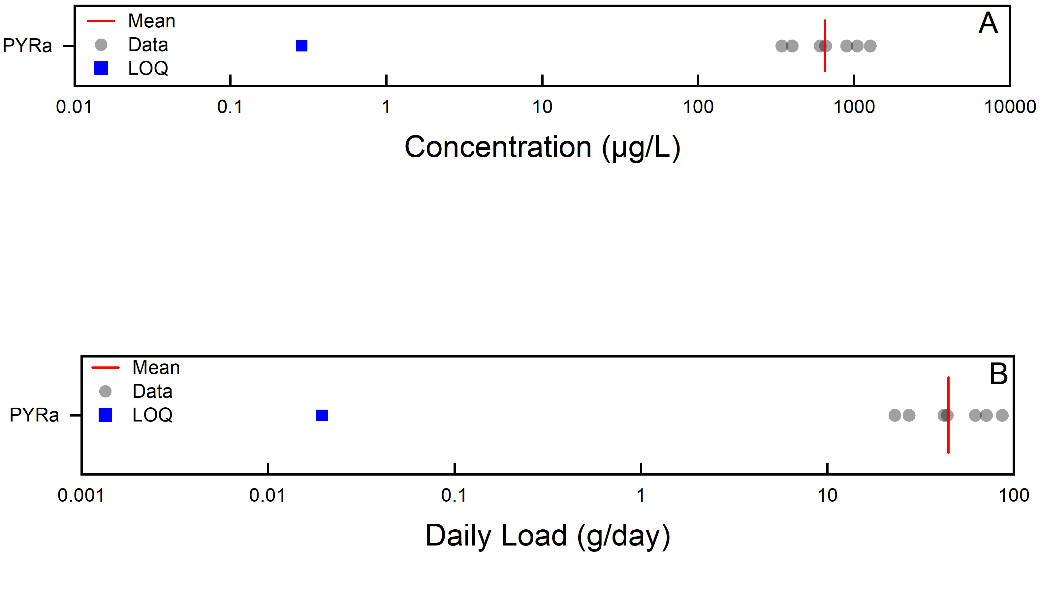


**Figure S17.** 7-day temporal study on the health markers sub-class ‘deficiency’ BCIs, quantified in influent wastewater. Figure A details the wastewater concentration, LOQ is determined via the MQL (Table 1). Figure B is the daily load, for the same samples. Here, daily load LOQ was calculated by multiplying the MQL by the average daily flow rate (L/day) across the 7-day sampling week. Abbreviations are defined within Table S1.


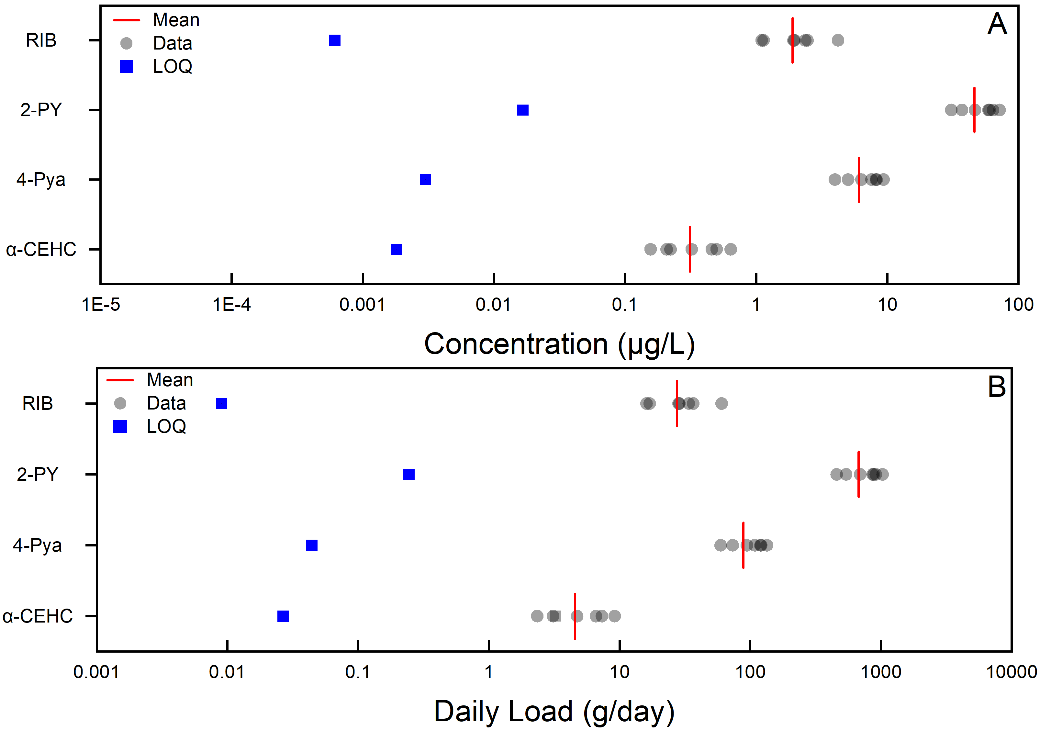


**Figure S18**. 7-day temporal study on the food sub-class ‘vitamins’ BCIs, quantified in influent wastewater. Figure A details the wastewater concentration, LOQ is determined via the MQL (Table 1). Figure B is the daily load, for the same samples. Here, daily load LOQ was calculated by multiplying the MQL by the average daily flow rate (L/day) across the 7-day sampling week. Abbreviations are defined within Table S1.


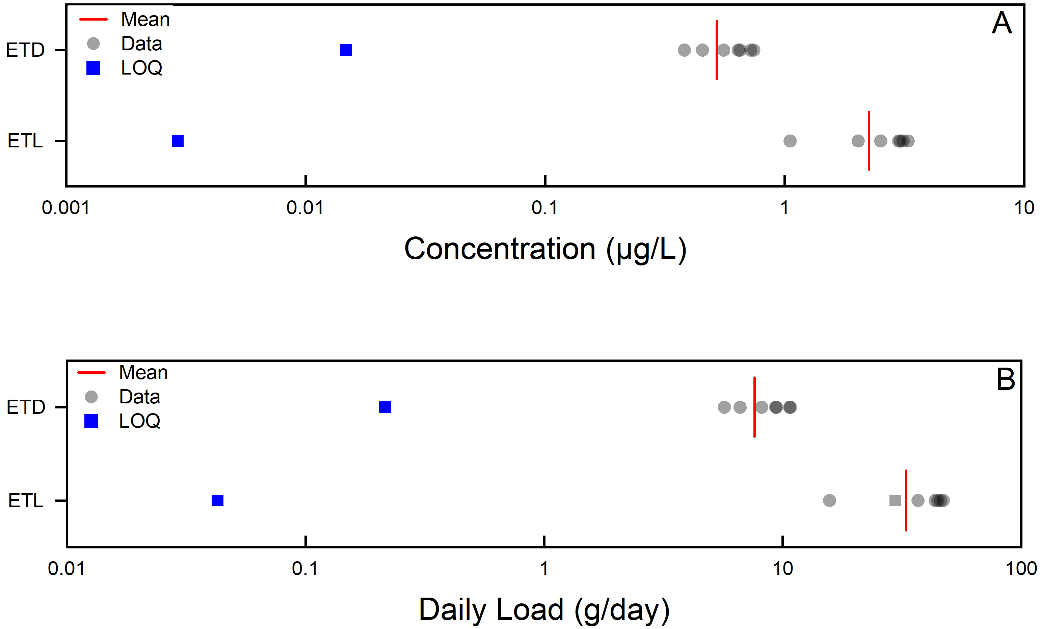


**Figure S19**. 7-day temporal study on the food sub-class ‘lignan’ BCIs, quantified in influent wastewater. Figure A details the wastewater concentration, LOQ is determined via the MQL (Table 1). Figure B is the daily load, for the same samples. Here, daily load LOQ was calculated by multiplying the MQL by the average daily flow rate (L/day) across the 7-day sampling week. Abbreviations are defined within Table S1.


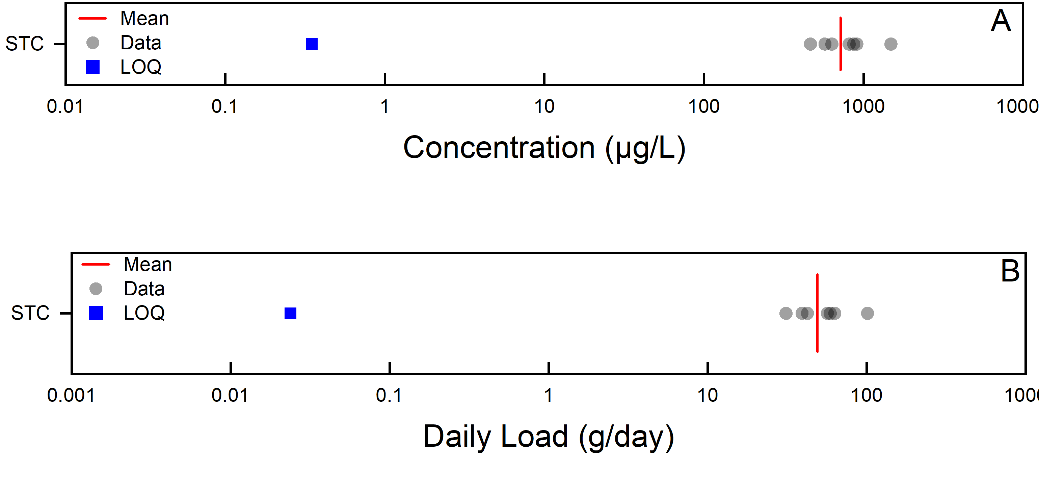


**Figure S20**. 7-day temporal study on the food sub-class ‘alkaloid’ BCIs, quantified in influent wastewater. Figure A details the wastewater concentration, LOQ is determined via the MQL (Table 1). Figure B is the daily load, for the same samples. Here, daily load LOQ was calculated by multiplying the MQL by the average daily flow rate (L/day) across the 7-day sampling week. Abbreviations are defined within Table S1.


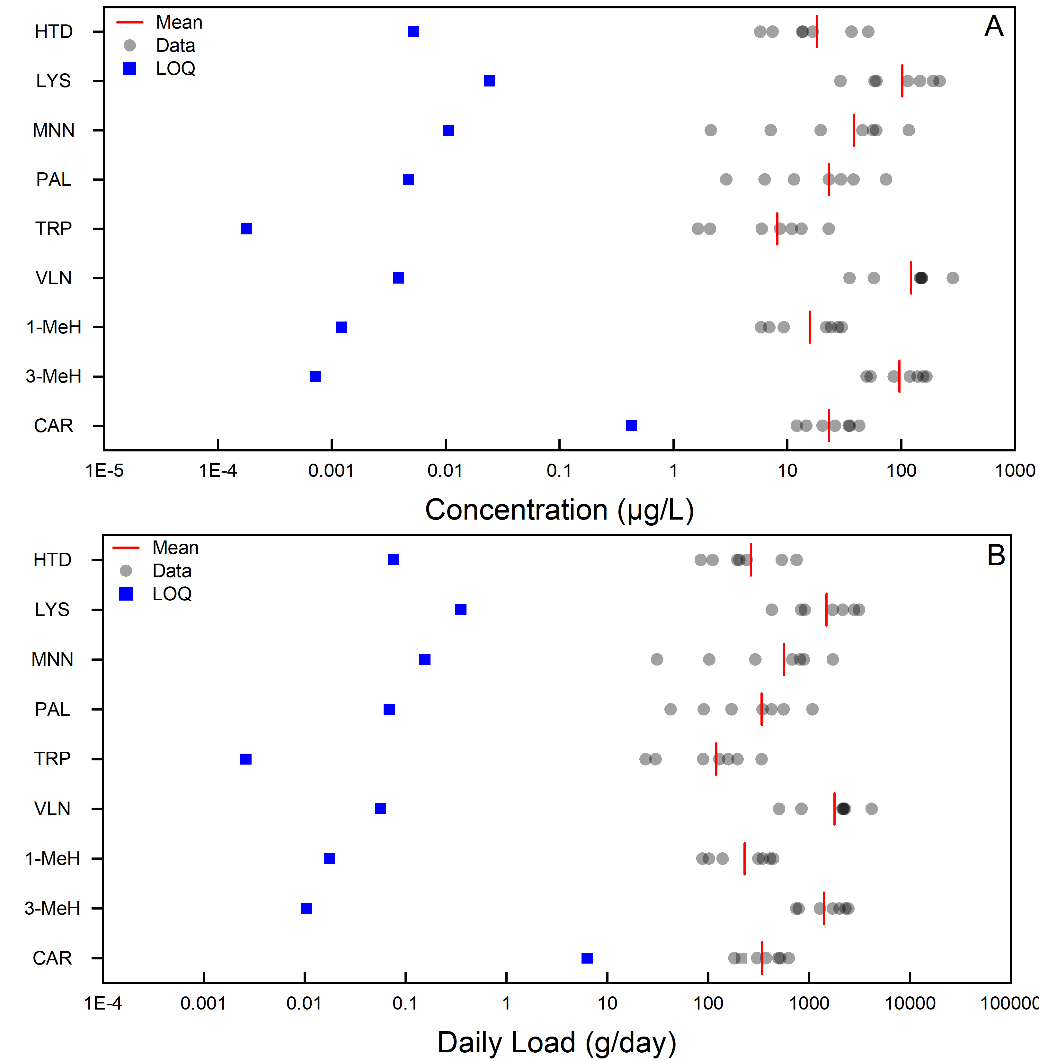


**Figure S21**. 7-day temporal study on the food sub-class ‘amino acid’ BCIs, quantified in influent wastewater. Figure A details the wastewater concentration, LOQ is determined via the MQL (Table 1). Figure B is the daily load, for the same samples. Here, daily load LOQ was calculated by multiplying the MQL by the average daily flow rate (L/day) across the 7-day sampling week. Abbreviations are defined within Table S1.

**Figure S22**. 7-day temporal study on the food sub-class ‘fish’ BCIs, quantified in influent wastewater. Figure A details the wastewater concentration, LOQ is determined via the MQL (Table 1). Figure B is the daily load, for the same samples. Here, daily load LOQ was calculated by multiplying the MQL by the average daily flow rate (L/day) across the 7-day sampling week. Abbreviations are defined within Table S1.


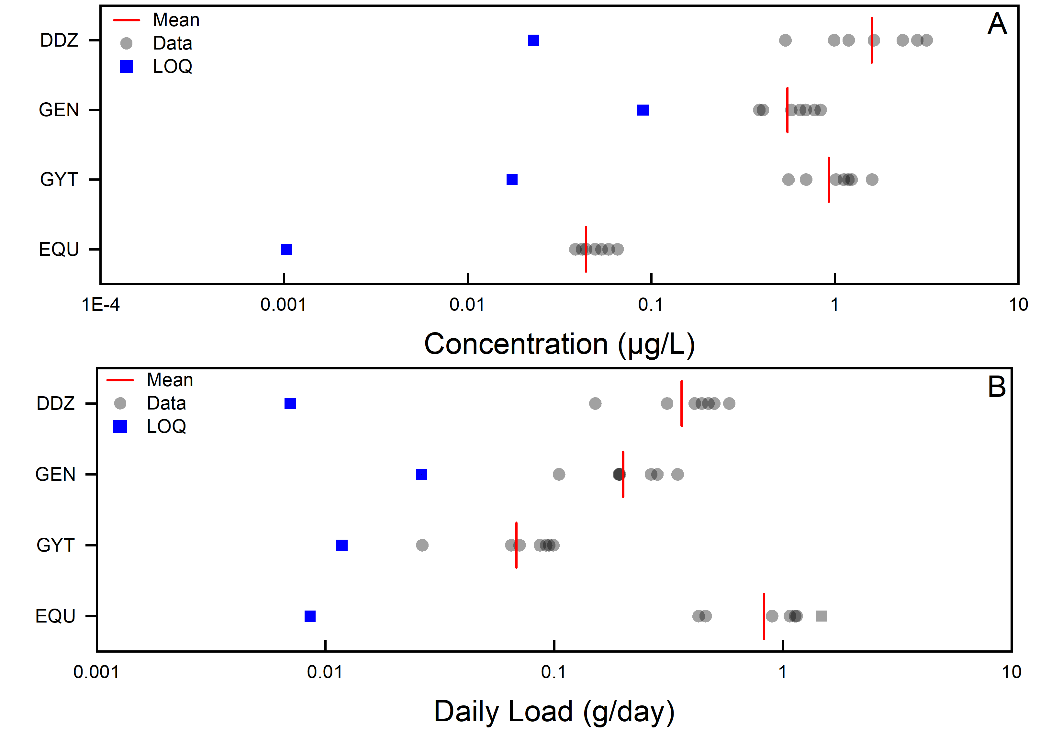


**Figure S23**. 7-day temporal study on the food sub-class ‘isoflavone’ BCIs, quantified in influent wastewater. Figure A details the wastewater concentration, LOQ is determined via the MQL (Table 1). Figure B is the daily load, for the same samples. Here, daily load LOQ was calculated by multiplying the MQL by the average daily flow rate (L/day) across the 7-day sampling week. Abbreviations are defined within Table S1.


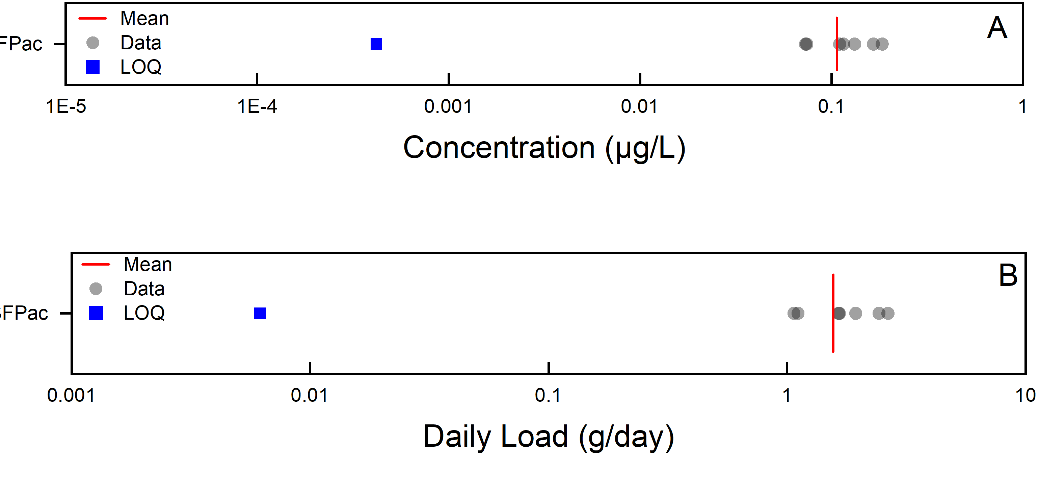


**Figure S24**. 7-day temporal study on the food sub-class ‘cruciferous vegetables’ BCIs, quantified in influent wastewater. Figure A details the wastewater concentration, LOQ is determined via the MQL (Table 1). Figure B is the daily load, for the same samples. Here, daily load LOQ was calculated by multiplying the MQL by the average daily flow rate (L/day) across the 7-day sampling week. Abbreviations are defined within Table S1.


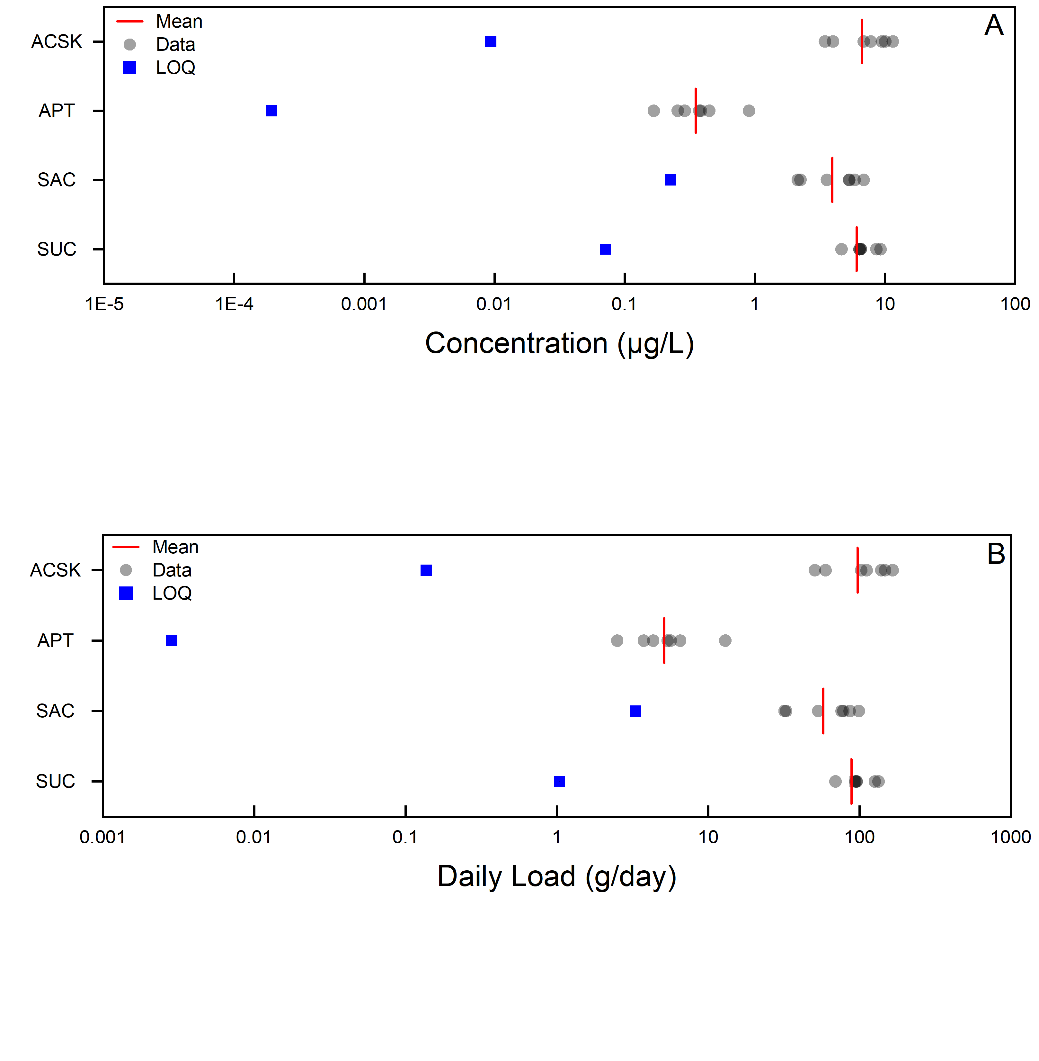


**Figure S25**. 7-day temporal study on the food sub-class ‘artificial sweetener’ BCIs, quantified in influent wastewater. Figure A details the wastewater concentration, LOQ is determined via the MQL (Table 1). Figure B is the daily load, for the same samples. Here, daily load LOQ was calculated by multiplying the MQL by the average daily flow rate (L/day) across the 7-day sampling week. Abbreviations are defined within Table S1.
